# A Synopsis of Western Botany

**Published:** 1835

**Authors:** John L. Riddell


### The Western Journal
OF THE
### MEDICAL AND PHYSICAL SCIENCES.

JANUARY, FEBRUARY, AND MARCH, 1835.

### ESSAYS AND CASES.

### ART. I.—WESTERN FLORA.

*Synopsis of the Flora of the Western States.*—By JOHN L. RIDDELL.

(Continued from page 374.)

### CLXXXVI.—*COMPOSITÆ.*

SUB-ORDER I.—CICHORACEÆ.—Generally lactescent.

*Tribe* 1. *Hieraceæ.*—Hawkweed like.

788. Hieracium venosum, *Linn.* Veiny hawkweed. Bloodwort. Jul.–Aug. *y.* 1 to 2 f. ♃. sw. W. This and other plants of the genus are doubtless possessed of bitter, astringent, and narcotic qualities.

789. H. paniculatum, *Willd.* Jul.–Sept. *y.* 1 to 2 f. *Bk.* 2 to 4 f. *Eat.* ♃. Woods. Ky. *St.* West Va. *Paddock.* O.

790. H. gronovii, *Linn.* Jul.–Aug. *y.* 2 to 3 f. ♃. Dry woods. Ky. *St.*

791. H. marianum, *Willd.* Rough hawkweed. Jul.–Aug. *y.* 2 f. ♃. Woods. Ark., &c. *Jas.*

792. H. runcinatum, *Jas.* 1 f. Plt. *Jas.*

793. Prenanthes alba, *Linn.* White lettuce. Aug. *y–w.* 5 f. ♃. Woods. sr. O. Ky.

794. P. serpentaria, *Pursh.* Lion's foot. Aug. *p.* 2 to 4 f. ♃. sw. O. Ky. It is said to cure the bite of the rattlesnake.

795. P. illinoiensis, *Pers.* Sept. Flowers sulphur colour. 2 to 4 f. ♃. dp. Brushy Prairie, Dayton, O. Prairies of Illinois. *Michx.*

796. P. racemosa, *Michx.* Sept. *y.* 2 to 4 f. ♃. wp. dp. Duncan's Plains, O.

797. P. altissima, *Linn.* Sept. *y.* 4 to 8 f. ♃. Mich. *Eat.*

798. P. juncea, *Pursh.* ♃. Plt. *Jas.*

799. P. crepidinea, *Michx.* Sept. Pale *y.* 4 to 6 f. ♃. New fields. Thickets. Wor. O. Ky. *St.*
800. P. deltoidea, *Ell.* Sept. *y.* 2 f. ♃. Ky. *St.*
801. P, miamiensis, *mihi. Leaves* of the *involucre* (calyx) 8, inclosing 15 florets: *flowers* nodding, pale purple, in bundles of 5 to 8, panicled: *lower leaves* on long petioles, (3 to 6 i.) deltoid, acuminate, irregularly dentate, near 7 inches long by 6 in width: *upper leaves* ovate, attenuate at the base, with one or two lateral teeth: *stem* branching above. 5 to 8 f. high. Whole plant glabrous. Sept. ♃. Thickets near Dayton, O.
802. P. ovata, *mihi. Involucre* purple, near 7-leaved, 10 to 12-flowered: *flowers* nodding, glabrous, in a terminal raceme, with 2 to 4 in a bunch: *petals* white: *lower leaves* on long petioles, (2 inches.) ovate, acute attenuate at the base, entire, glabrous above, slightly pubescent beneath: *upper leaves* lance-ovate, nearly sessile: *stem* simple, glabrous, 1½ to 2 f. ♃. dp. Brushy Prairie, Dayton, O. Flowers in Sept.
803. P. parviflora, *mihi. Involucre* green, 5-leaved, 5-flowered: *flowers* very small, in axillary bundles of 2 to 5, and in a small, terminal panicle, nodding, glabrous: *leaves* generally decurrent into long petioles, denticulate, acuminate, sub-pubescent beneath: *radical leaves* large, (6 inches long by 5 in width,) deltoid, rather coarsely dentate: *lower cauline leaves* hastate-sagitate: *middle* ones irregularly cordate; *upper* ones ovate: *stem* simple, 2 to 4 f. ♃. Slaty ravines, Worthington, O. Flowers in Aug.
804. P. proteophylla, *mihi.* Protean-leaved prenanthes. *Involucre* greenish purple, 8 or 9-leaved, 10 or 11-flowered: *flowers* nodding, in a racemose, terminal panicle; and in axillary bundles of 2 to 4: *leaves* petioled, glabrous, acute, 2 to 4 inches long: general outline mostly ovate; varying indefinitely from anomalous projections, and irregular gashes: *stem* simple, 2 to 3 f. ♃. Calcareous ravine on the Scioto, near Dublin, O. Rare. Flowers in Sept.

*Tribe* 2. *Taraxaceæ.*—Dandelion like.

805. Leontodon taraxacum, *Linn.* Dandelion. Ap.–Nov. *y.* 6 to 12 i. ♃. Pastures, &c. Intr. W. Tonic, diuretic, aperient, hepatic deobstruent. Wood and Bache, 635. British Med. Fl.
806. Krigia amplexicaulis, *Nutt.* Clasping dandelion. June. *y.* 1 f. ♃. Wet woods. Mich. *Eat.* Ohio. *Eberle.* West Va. *Paddock.*

807. K. virginica, *Linn.* Dwarf dandelion. May–Aug. Deep *y.* ⊙. 2 to 8 i. Arid soils. Louisville· *M'Murt.*
808. Troximon glaucum, *Pursh.* Jul. *y.* ♂. Mo. Ter. *Nutt.* Rare.
809. T. cuspidatum, *Pursh.* May. *y.* Grassy plains on the Missouri. *Nutt.*

*Tribe* 3. *Lactuceæ.*—Lettuce like.

810. Lactuca elongata, *Muhl.* Wild lettuce. Milkweed. Fire-weed. Aug.–Sept. *y.* 3 to 6 f. ♂. Newly cleared lands. Thickets. Ky. O. Sedative, laxative, diuretic, diaphoretic. Wood and Bache, 381.
811. L. integrifolia, *Big.* Jul. *y.* 3 to 4 f. ♂. rd, &c. Mo. Ter. *Nutt.* Near Cin. O. Properties like the last?
812. L. hirsuta, *Nutt.* Aug. *y* & *p.* ♃. O?
813. L. canadensis, *Linn.* Sonchus pallidus, *Pursh.* Canadian milkweed. Jul.–Sept. *y.* 2 to 3 f. ♃. Woods. rd. Miami country. Medicinal? like 810.
814. L. villosa, *Jacq.* Sonchus acuminatus, *Willd.* Aug.–Sept. *p.* 3 to 4 f. ♂. sw. Miami country. Medicinal?
815. L. ludoviciana, *mihi.* Sonchus ludovicianus, *Nutt.* June. *y.* 3 to 5 f. ♃. Humid places. Mo. Ter. *Nutt.* O. Ky. I have not the means of ascertaining where this species is placed by Don.
816. Agathyrsus leucophæus, *Don.* Jul.–Sept. Bluish *w.* 2 f. ♂. rd. ow. Louisville. *M'Murt.* Miami country.
817. A. floridanus, *Don.* Gall of the earth. Jul.–Sept. *b.* 3 to 5 f. ♂. ow. rd. O. A reputed cure for the bite of the rattlesnake.
818. A. macrophyllus, *Don.* Aug.–Sept. *b.* 4 to 7 f. ♃. Humid situations. Miami country.
819. Sonchus oleraceus, *Linn.* Sow thistle. Jul.–Sept. *y.* 2 to 4 f. ⊙. Waste grounds. cf. Intr. W.
820. S. arvensis, *Linn.* Jul. *y.* 2 f. ♃. cf., &c. Intr. O.
821. S. spinulosus, *Big.* Native sow thistle. Aug. *y.* 2 f. ⊙. Salt marshes. *Bk.* Woods. rd. &c. Ky. St. Miami country.

Sub-order II. Carduaceæ.—The thistle and its allies.

822. Arctium lappa, *Linn.* Burdock. Jul.–Aug. *p.* 3 to 4 f. ♃. Waste grounds. Intr. W. Aperient, diaphoretic, alterative. Wood and Bache, 105. Eberle. Beach.
823. Carduus lanceolatus, *Linn.* Common thistle. Sept. *p.* 2 to 4 f. ♃. ♂. rd. &c. W.
824. C. altissimus, *Linn.* Tall thistle. Sept. *p.* 3 to 8 f.

(12 to 18 f. on the Missouri. *Nutt.*) ♃. ow. Old fields, W.

825. C. virginanus, *Willd.* Virginan thistle. Jul.–Sept. *p.* 2 to 3 f. ♃. Woods. O. Ky.

826. C. glutinosus, *Bk.* Balsam thistle. Aug.–Sept. *p.* 4 to 6 f. ♂. wp. dp. O.

827. C. discolor, *Nutt.* Jul.–Sept. *p.* 3 to 8 f. ♂. (Margins of swamps. 3 to 6 f. Jul.–Aug. *Bk.*) dp. Middletown, O.

828. C. muticus, *Nutt.* Aug.–Sept. *p.* 2 to 4 f. ♂. Mts. and low grounds. *Bk.* Mich. *Eat.*

829. C. pitcheri, *Torr.* 18 i. Near Lakes Huron and Michigan. *Houghton. James.* On sand banks.

830. C. undulatus, *Nutt.* *r–p.* 1 to 2 f. Near Lake Huron. *Nutt.*

831. Elephantopus carolinianus, *Willd.* Elephant's foot. Sept. *p.* 2 f. ♃. Dry soils. Near Dayton, O. *Eberle.*

832. Vernonia noveboracensis, *Willd.* Iron-weed. Flat-top. Aug.- Sept. *p.* 4 to 6 f. ♃. bt. Wet grounds. W. Medicinal?

833. V. præalta, *Willd.* Also called iron-weed. Aug.–Oct. *p.* 5 to 7 f. ♃. md. bt. O. Dr. Stephens of Monroe, O., says that the root of the iron-weed is reputed to be a very active purgative.

834. V. corymbosa, *Schweinitz.* *r.* 1 f. *Eat.* (Aug. *r–p.* 1 to 3 f. ♃. wp. Darby Plains, O)?

835. V. altissima, *Nutt.* Aug. *p.* 6 to 12 f. ♃. bt. Banks of streams. O. Ky. *Nutt.* Ark. *Jas.*

836. V. baldwinii, *Torr.* Above St. L. *Bk.*

837. V. fasciculata, *Michx.* Aug. ♃. Prairies of Illinois. *Michx.*

838. Liatris spicata, *Willd.* Ohio devil's bit. Gay feather. Aug.–Sept. *p.* 3 to 6 f. ♃. dp. O. Ky. Stimulant, carminiative, diaphoretic. Thompsonians and others.

839. L. squarrosa, *Willd.* Button snakeroot. Aug.–Oct. *p.* 1 to 2½ f. ♃. Sandy woods. *Bk.* dp. O. Ky. W. Medicinal. See 838.

840. L. scariosa, *Willd.* Aug.–Oct. *p.* 3 to 4 f. ♃. Sandy woods, &c. O.

841. L. graminifolia, *Walt.* Sept. *p.* 2 to 4 f. ♃. Wet pine barrens. *Ell.* Ky. *St.*

842. L. gracilis, *Pursh.* Sept. *p.* 2 to 3 f. ♃. Pine barrens. *Ell.* Oak barrens, near Mar. O.

843. L. cylindracea, *Michx.* Aug. *p.* 1 to 2 f. ♃. Woods and prairies of Ill. *Pursh.* Mich. *Eat.*

844. L. pycnostachya, *Michx.* Sept. 2 to 4 f. ♃. Ill. &c. *Pursh.*

845. L. aspera, *Michx.* Sept. ♃. Ill. &c. *Michx.*
846. Conyza camphorata, *Pursh.* Marsh fleabane. Aug.–Sept. *p.* 1 to 2 f. ♃. Salt marshes. *Bk.* rb. Ky. *St.* Cin. O. Medicinal?
847. Inula helenium, *Linn.* Elecampane. Jul.–Aug. *y.* 3 to 4 f. ♃. rd. &c. W. Tonic, stimulant, diaphoretic, diuretic, emenagogue. Wood and Bache, 355.
848. Arnica fulgens, *Pursh.* Variety of A. montana, *Nutt.* Leopard's bane. Jul. *y.* 1 f. ♃. Margins of marshy springs. Mo. Ter. *Nutt.* Stimulant, diaphoretic, emenagogue, &c. Wood and Bache, 108.
849. Chrysopsis mariana, *Nutt.* Aug.–Oct. *y.* 1 to 2 f. ♃. Sandy woods. Ky. *St.*
850. C. villosa, *Pursh.* *y.* ♃. Mo. Riv. *Nutt.*
851. Zinnia multiflora, *Linn.* Blood marygold. Sept. ⊙. Banks of the Miss. *Pursh.*
852. Gnaphalium polycephalum, *Michx.* Sweet balsam. Fragrant life everlasting. Jul.–Sept. Pale *r.* 1 to 2 f. ⊙. Fields. W. Astringent, pectoral, corroborant.
853. G. americanum, *Linn.* American cudweed. Jul.–Sept. Pale *y.* 6 to 10 i. ⊙. ow. rs. Mar. O.
854. G. purpureum, *Linn.* Jul.–Oct. Pale *p.* 8 to 12 i. ♃. Barren soil. Ky. *St.*
855. G. uliginosum, *Linn.* Marsh cudweed. Aug.–Sept. Yellowish brown flowers. 3 to 6 i. ⊙. rd. mh. Wor. O. Ky.
856. G. plantagineum, *Linn.* Plantain cudweed. May June. *r–w.* 8 to 10 i. ♃. Woods, &c. wp. O. Ky.

SUB-ORDER III. ASTEREÆ.—Star-flower like.

*Aster.* Star-wort, or star-flower.

§1. *Leaves entire.*

857. Aster hyssopifolius, *Linn.* Aug.–Oct. *w–p.* 1 to 2 f. ♃. Sandy fields. Louisville. *M'Murt.*
858. A. solidaginoides, *Michx.* Aug.–Oct. *w.* 2 f. ♃. Woods. Louisville. *M'Murt.*
859. A. tenuifolius, *Linn.* Aug.–Nov. *w–p.* 2 to 3 f. ♃. rd. Fields. O. Louisville.
860. A. linarifolius, *Linn.* Sept.–Oct. *w–p*, & *y.* 1 to 2 f. ♃. dp. rs. Duncan's Plains, O. Ky. *St.*
861. A. linifolius, *Linn.* Sept.–Oct. *w*, or pale *p.* 18 i. to 2 f. ♃. sw. Louisville. *M'Murt.*
862. A. ericoides, *Willd.* Aug. Pale *p.* 2 to 3 f. ♃. Barren soils. Ky. *St.*
863. A. paludosus, *Ait.* Aug.–Nov. *b.* 1 to 2 f. ♃. Borders of swamps. Mo. Ter. *Jas.*

864. A. multiflorus, *Ait.* Aug. *w* & *y.* 2 to 3 f. ♃. rd. Fields. Ky. *St.*

865. A. concolor, *Linn.* Aug.–Nov. *b.* 2 to 3 f. ♃. Pine woods. Fox Riv. N. W. Ter. *Houghton.*

866. A. cornifolius, *Muhl.* Aug. *w.* 2 to 3 f. ♃. ow. &c. O. Ky.

867. A. humilis, *Willd.* Aug. *w.* 1 f. ♃. Ky. *St.*

868. A. amygdalinus, *Lam.* Aug.–Sept. *w.* 2 f. ♃. Low grounds. O. Ky.

869. A. novæ-angliæ, *Linn.* Sept.–Nov. *p* or *b.* 3 to 6 f. ♃. md. wp. dp. O. Ky.

870. A. cyaneus, *Pursh.* Sept.–Nov. *p* & *b.* 3 to 4 f. ♃. md. dp. Duncan's Plains, O.

871. A. phlogifolius, *Willd.* Aug.–Nov. *p.* 1 to 2 f. ♃. Moist grounds. *Bk.* ow. Ky. *St.* Near Dayton, O.

872. A. carolinianus, *Walt.* Oct. *p.* 10 to 12 f. ♃. Swamps. Ky. *St.*

873. A. canescens, *Pursh.* Aug.–Oct. *b.* 1 f. ♂. Mo. Ter. *Nutt.*

874. A. pauciflorus. *Nutt.* Aug. *w.* 10 i. ♃. Margins of salt springs. Mo. Ter. *Nutt.*

875. A. sericeus, *Nutt.* ii. 155 *Vent?* Spreng. iii. 525. *p.* 12 to 18 i. ♃. Ill. *Nutt.* Mo. Ter. *Houghton.*

876. A. flexuosus, *Nutt.* Sept.–Oct. Pale *p.* 6 to 18 i. ♃. Salt marshes usually. Ky. *St.*

877. A. montanus, *Nutt. p.* 1 to 2 f. Mts. Tenn. *Nutt.*

878. A. oblongifolius, *Nutt. p.* 1 f. ♃. Banks of the Missouri. *Nutt.*

879. A. albus, *Nutt.* Aug. *w.* 1 f. ♃. Mo. Ter. *Nutt.*

§2. *Leaves lanceolate and ovate, the lower ones serrate.*

880. A. puniceus, *Linn.* Sept.–Nov. *p.* 6 to 8 f. ♃. (Salt swamps, *Bk.*) dp. wp. md. bt. O.

881. A. novi-belgii, *Linn.* Aug.–Oct. Pale *p.* 3 f. ♃. Fields. Ky. *St.*

882. A. gracilis, *Nutt.* Pale *b.* 1 f. ♃. Savannahs of Ky. and Tenn. *Nutt.*

883. A. prenanthoides, *Willd.* Aug.–Oct. *b.* 3 to 4 f. ♃. cr. bt. O. Ky.

884. A. lævis, *Linn.* Sept.–Nov. *p.* 2 f. ♃. Woods and sides of ponds. Ky. *St.* N. W. Ter. *Houghton.*

885. A. conyzoides, *Willd.* Jul.–Aug. *w.* 1 to 2 f. ♃. Copses, &c. O. Ky.

886. A. miser, *Linn.* Aug.–Nov. *w.* 1 to 3 f. ♃. dp. O. Ky.

887. A. divergens, *Ait.* Var. of miser, *Torr.* Sept. *w–r.* 3 to 5 f. ♃. Ky. *St.*

888. A. diffusus, *Ait.* Var. of miser, *Torr.* Sept.–Nov. *w.* ♃. Ky. *St.*

§ 3. *Leaves cordate and ovate, serrate.*

889. A. cordifolius, *Linn.* Sept.–Nov. *w.* or pale *p.* 2 to 3 f. ♃. Woods, in hilly districts. O. Ky.

890. A. macrophyllus, *Linn.* Sept.–Oct. *w* or pale *b.* 1 to 2 f. ♃. rs. sr. sw. Wor. O. Louisville. *M'Murt.*

891. A. undulatus, *Linn.* divesifolius, *Michx.* Sept. Pale *b.* 2 to 3 f. ♃. Old fields, &c. O. Ky.

892. A. Shortii. *Flowers* in a spreading panicle: *leaves* nearly glabrous above, scabrous and sparingly pilose beneath: *radical leaves* heart-oblong, pubescent when young, remotely and deeply crenate, almost obtuse: *cauline leaves* mostly on long ciliate petioles, entire, cordate-lanceolate, acute: *floral leaves* (bracts) ovate and lanceolate, sessile, minute, entire: *stem* branching above. Oct. *b.* 2 f. ♃. On hill-sides and in ravines. Wor. Ciu. O. Ky. The specific name was proposed by Dr. Boot, of London, in honour of one of our most distinguished botanists, Dr. C. W. Short, of Lexington, Ky. Whether the species has been published or not by Dr. Boot, I do not know. The above brief description was taken from dried specimens.

893. A. oolentangiensis, *mihi.* *Panicle* terminal, elongated, few flowered: *leaves* acuminate, scabrous above, rough and pubescent beneath: *radical leaves* long petioled, ovate, obliquely cordate, remotely serrate: *lower cauline leaves* lanceolate, (2 to 4 inches in length,) remotely serrate, on long winged petioles : *upper leaves* lanceolate, half clasping, entire: *stem* simple, strigose. Sept.–Oct. Pale *b.* 2 f. ♃. sw. Wor. O. Forests on the Oolentangy River. I first discovered this plant in 1832, on the large, forest clad mound, near Worthington, O.

894. Erigeron purpureum, *Linn.* Purple erigeron. Jul.–Aug. *p.* 12 to 18 i. ♃. ow. &c. O. Ky.

895. E. philadelphicum, *Linn.* Aug.–Sept. Pale *p.* 2 to 3 f. ♃. ow. &c. W. Diuretic, antilithic. Bart. Med. Bot. 227. Wood and Bache, 284.

896. E. heterophyllum, *Linn.* Scabius. Flea-bane. June.–Aug, *w.* 2 to 3 f. ♂. Waste grounds, O. Diuretic, antilithic. Wood and Bache, 284. Bart. i. 231.

897. E. strigosum, *Linn.* Jul.–Aug. *w.* 2 to 3 f. ♂. md. ow. O. Ky.

898. E. bellidifolium, *Linn.* Robin's plantain. June–Aug. Pale *p.* 12 to 18 i. ♃. sw. Ky. *St.* O.

899. E. pumilum, *Nutt.* Low erigeron. May. *w.* 4 i. ♃. Plains of the Missouri. *Nutt.*

900. E asperum, *Nutt.* Rough erigeron. Aug. *w.* 12 i. Plains of the Missouri. *Nutt.*

901. E. glabellum, *Nutt.* Aug. Pale *b.* 12 to 18 i. ♃. Plains of the Missouri. *Nutt.*

902. E. divaricatum, *Michx.* Aug. 6 i. Spreading. 1 to 2 f. ☉. Ky. St. L. *Nutt.*

903. E. canadense, *Linn.* Flea-bane. Jul.–Sept. 2 to 6 f. ☉. Dry fields. ow. W. Diuretic, tonic, astringent. Wood and Bache, 283.

*Solidago.*—Golden rod. Flowers yellow.

§ 1. *Racemes secund; leaves with three combined nerves.*

904. Solidago canadensis. *Linn.* Aug.–Sept. 2 to 5 f. ♃. Fields. Ky. *St.*

905. S. procera, *Ait.* Jul.–Sept. 4 to 7 f. ♃. Swamps and low grounds. Louisville *M'Murt.*

906. S. serotina, *Ait.* Sept.–Oct. 4 f. ♃. Woods. Ky. *St.*

§ 2. *Racemes secund; leaves veined.*

907. S. nemoralis, *Ait.* Aug.–Oct. 1 to 2 f. ♃. Sandy fields. Louisville. *M'Murt.*
908. S. ulmifolia, *Willd.* Aug.–Oct. 3 to 4 f. ♃. sw. Louisville. *M'Murt.*
909. S. recurvata, *Willd.* Sept.–Nov. 3 to 4 f. ♃. sw. O.
910. S. odora, *Ait.* Golden rod tea plant. Aug.–Oct. 3 f. ♃. ow. &c. O. Ky. Aromatic, stimulant, carminiative. Big. Med. Bot. i. 187. Wood and Bache, 610.

§ 3. *Racemes erect.*

911. S. bicolor, *Linn.* Aug.–Oct. 1 to 2 f. ♃. Dry hills. Ky. *St.*
912. S. stricta, *Ait.* Aug.–Oct. 2 f. ♃. Sandy woods, *Bk.* Prairies, O.
913. S. cæsia, *Ait.* Aug.–Oct. 2 f. ♃. Sandy woods. Ky. *St.*
914. S. latifolia, *Muhl.* Aug. 2 f. ♃. rs. sw. O. Ky.
915. S. flexicaulis, *Pursh.* Aug. 2 to 3 f. ♃. Woods. Ky. *St.*
916. S. axillaris, *Pursh.* Aug.–Oct. 2 to 3 f. ♃. Woods. O. Ky.
917. S. cordata, *Short* and *Peter.* Aug.–Oct. 2 to 4 f. ♃. Steep, wooded hill-sides, on the banks of the Kentucky river. *St.* Described in Transyl. Med. Jour. for 1834, page 599.
918. S. tenuifolia, *Pursh.* Sept.–Oct. 12 to 28 i. ♃. Pine barrens, *Bk.* dp. O.
919. S. rigida, *Linn.* Bone's styptic. Aug.–Oct. 3 to 4 f. ♃.

Mts. *Bk.* dp. Duncan's plains, O. Said to be a valuable styptic, for suppressing hæmorrhage from recent wounds. The leaves are used.

920. S. ohioensis, *mihi*. *Root* perennial, creeping?: *stem* simple: *radical leaves* (when they precede the stem) on long equitant petioles, oblong, retuse, acute and entire at the base, serrate towards the summit, margin scabrous, (near 2 inches wide, by 4 to 6 in length): *cauline leaves* entire, half clasping, lanceolate, acute: *corymb* fastigate. Whole plant glabrous, the margin of the leaves excepted. Seeds 16 to 20, scales of the involucre 8 to 13, oblong, obtuse. Oct. *y*. 2 to 3 f. Wet grassy prairies, Ohio. Van Cleve's prairie, Dayton. Two miles S. from Columbus, O. Pretty closely allied to S. rigida.

921. S. Riddellii, *Frank*.* *Root* perennial, creeping: *stem* simple: *leaves* lance-linear, glabrous, acute, entire, margin scabrous: *radical leaves* on carinate, equitant petioles, (10 to 12 inches long), often falcate, 5-nerved, with 2 additional nerves towards the summit, (6 to 8 lines in width. 8 to 10 inches long): *cauline leaves* carinate, sheathing, upper ones lance-oblong: *flowers* in a corymbose fascicle: *involucre* imbricate; scales 8 to 12, appressed, oblong-obovate, obtuse: *rays* 7 to 9, narrow, scarcely exceeding the disk: *disk florets* 10 to 12. Sept.–Oct. *y*. 2 f. Grassy prairies, Ohio. Scott's plains, 12 miles E. from Worthington. Hoffman's prairie, 8 miles E. from Dayton. Allied to the last, more closely than to any other species of my acquaintance. The leaves have a grass-like aspect.

922. Chrysocoma graveolens, *Nutt*. Oct. *y*. 6 to 8 f. ♄. Denuded soils, Missouri banks. *Nutt*.

923. C. nouseosa, *Pursh*. Oct. *y*. ♃. Banks of the Missouri. *Nutt*.

924. Bellis integrifolia, *Michx*. Kentucky daisy. (Sept. *w–r*.)? 8 to 12 i. Ky. *St*. Kentucky river. *Peter*.

Sub-order IV. Eupatorineæ.—Boneset like.

925. Eupatorium sessilifolium, *Linn*. Upland boneset. Aug.–

* S. radice perenni; foliis integerrimis, glabris, margine scabris: inferioribus radicalibusque longissime petiolatis, subfalcato-lanceolatis, subacutis, distincte 5-nerviis, superioribus vaginiantibus, oblongo-lanceolatis, acutiusculis, obsolete nervosis; petiolis carinatis, equitantibus; caule glabro, striato, corymboso-paniculato; racemis vel fasciculis 3-floris; calycis squamis obtuse ovatis ligulis duplo brevioribus; pedicellis bracteatis, villosiusculis.—*Frank*.

Sept. *w.* 2 to 4 f. ♃. Hills about Marietta, O. Ky. Tonic, &c. Raf. Med. Fl.

926. E. purpureum, *Linn.* Purple thoroughwort. Improperly called queen of the meadow. Aug.–Oct. *p.* 5 to 8 f. ♃. Wet woods. bt. W. Bitter, aromatic, astringent, diuretic. Wood and Bache, 287. Also hydragogue, antilithic. Beach.

927. E. verticillatum, *Muhl.* Joe-pye's weed. Aug.–Oct. Pale *p.* 4 to 6 f. ♃. Low grounds. bt. sw. O. Ky. Mich. Properties doubtless like the plant, very similar to the last.

928. E. maculatum, *Linn.* Aug.–Oct. Pale *p.* 4 to 5 f. ♃. Low grounds. Mar. O.

929. E. punctatum, *Willd.* Aug.–Oct. *p.* 3 to 4 f. ♃. Mts. *Pursh.* Mich. *Eat.*

930. E. perfoliatum, *Linn.* Boneset. Thoroughwort. Aug.–Sept. *w.* 2 to 4 f. ♃. wp. mh. &c. W. Tonic, diaphoretic, emetic, aperient. Bart. ii. 125. Big. i. 33. Wood and Bache, 286.

931. E. ageratoides, *Linn.* White snakeroot. Aug.–Sept. *w.* 2 f. ♃. Woods. W. Antispasmodic, diuretic, diaphoretic. Used in nervous diseases. Dose ℈i, in infusion. See Howard's works, wherein this plant is figured.

932. E. ovatum, *Big.* Jul.–Aug. *w.* 3 to 4 f. ♃. Low grounds. Ky. *St.*

933. E. rupestre, *Rafinisque.* Ky. *St.*

934. E. falcatum, *Michx.* Banks of the Ohio and Scioto rivers. *Michx.* Probably identical with 927.

935. E. altissimum, *Willd.* Aug. *w.* 3 to 7 f. ♃. Banks of the Mississippi and Missouri. *Pursh.*

936. Cælestina cærulea, *Cassin.* Mist flower. Aug.–Oct. *b.* 1 to 2 f. (2 to 3 f. *Bk.*) ♃. Woods. bt. Near Mar. O. Ky. W.

937. Kuhnia critonia, *Linn.* (Aug.–Sept. Pale *y.* Mts. *Bk.*) Sept. Pale *y.* 2 to 3 f. ♃. dp. Miami country.

938. Mikania scandens, *Willd.* Climbing thoroughwort. Jul.–Sept. *b–w.* Climbing. ♃. Low grounds. Ky. *St.*

939. Donia squarrosa, *Pursh.* Aug. *y.* 3 to 4 f. ♂. Calcareous hills, St. L. *Nutt.*

Sub-order V. Jacobeæ.—Fire-weed like.

940. Cacalia suaveolens, *Linn.* Aug.–Sept. *w.* 3 to 4 f. ♃. wp. md. Banks of streams. O. Ky.

941. C. atriplicifolia, *Linn.* Aug.–Sept. *w.* 3 to 6 f. ♃. dp. O. Ky.

942. C. reniformis, *Willd.* Aug.–Sept. *w.* 5 to 8 f. Low grounds. ow. Miami country.
943. C. tuberosa, *Nutt.* *w.* 4 to 6 f. Shady hills, around St. Louis. *Nutt.*
944. Tussilago palmata, *Ait.* Ap.–May. ♃. Islands of Lake Huron. *Nutt.* Lake Mich. *Hought.* (Demulcent, expecterant.)?
945. Senecio vulgaris, *Linn.* Groundsel. May–Oct. *y.* 18 i. ♃. cf. Intr. Louisville. *M'Murt.* Refrigerant, antiscorbutic. Hoop. Dic.
946. S. hieracifolius, *Linn.* Fire-weed. Jul.–Aug. *w.* 2 to 6 f. ⊙. New fields. O.
947. S. elongatus, *Pursh.* Jul.–Aug. ♃. rs. Banks of streams. (*y.* 1 to 2 f. Cin. O.)?
948. S. obovatus, *Willd.* May–June. *y.* 1 to 2 f. ♃. Rocky hills. sr. Sides of clayey hills. O. Ky.
949. S. balsamitæ, *Willd.* June–Jul. *y.* 1 to 2 f. ♃. Damp grounds. O. Ky.
950. S. gracilis, *Pursh.* May–Aug. *y.* 1 f. ♃. Rocky banks, *Bk.* (wp. Damp grounds, mh. Wor. O. 7 miles N. from Cin.) The Indian name for this and the preceding species, is *nut-quaw.* The root of either, given in infusion two or three times a day, is said to be a most excellent emenagogue.
951. S. aureus, *Linn.* Rag-wort. False valerian. June–Jul. *y.* 2 f. ♃. sw. md. Ky. St. Medicinal?
952. Cineraria canadensis, *Willd.* Canadian ashwort. May. *y.* 8 i. ♃. With 948. Worthington, O. Rare.
953. C. integrifolia, *Willd.* Aug. *y.* ♃. Mo. Ter. *Jas.*
954. Bœrbera glandulosa, *Nutt.* ii. 166. Denudated soil, banks of the Miss. and Mo. *Nutt.*

Sub-order VI. Helianthea.—Sunflower like.

*Helianthus.* Sunflower. Flowers yellow. There is much confusion in this genus. The prairies of Ohio produce several species which are as yet unpublished. I have drawn some descriptions from living plants, which I shall be happy to communicate, accompanied with specimens, to any botanist who will attempt a complete monograph. As it is inconsistent with the design of this Synopsis to insert long and minute descriptions, I prefer to omit all but such as have heretofore been determined.

§ 1. *Leaves opposite.*

955. Helianthus atrorubens, *Linn.* Aug.–Sept. Disk *p.* 3 to 4 f. ♃. Gravelly soil. W. *Bk.*

956. H. frondosus, *Willd.* Aug.–Sept. 4 f. ♃. Woods. Ky. *St.*

957. H. trachelifolius, *Willd.* Aug.–Oct. 3 to 4 f. ♃. Woods. dp. O. Ky.

958. Hispidulus, *Ell.* Sept. 3 to 4 f. ♃. Mich. *Eat.* Pine barrens, *Ell.*

959. H. truncatus, *Schw.* Sept. 2 f. ♃. ow. Hills, Mar. O.

960. H. scaberrimus, *Ell.* Sept. 4 to 6 f. ♃. (dp. Darby Plains, O.)?

961. H. tubæformis, *Willd.* ⊙. Banks of the Missouri. *Nutt.* Seed ground into meal by the Indians, for food.

962. H. pubescens, *Willd.* Aug. 2 to 3 f. ♃. Ky. *St.*

§ 2. *Upper leaves alternate.*

963. H. giganteus, *Linn.* Aug.–Sept. 5 to 8 f. ♃. dp. &c. O.

964. H. altissimus, *Linn.* Jul.–Sept. 5 to 8 f. ♃. Mountain meadows, *Bk.* Ky. *St.* Plt. *Jas.*

965. H. strumosus, *Linn.* Aug.–Oct. 3 to 5 f. ♃. ow. dp. O.

966. H. decapetalus, *Linn.* Aug.–Oct. 3 to 4 f. ♃. Rocky woods, *Bk.* O. Ky. N.W. Ter.

967. Rudbeckia purpurea, *Willd.* Purple cone-flower. July–Sept. *p.* 3 to 4 f. ♃. Barrens. dp. O. Ky. Root aromatic, carminiative. Figured, Bart. Fl. N. A. ii. 84.
968. R. hirta, *Linn.* Hairy cone-flower. Aug.–Sept. *y.* 2 to 3 f. ♃. dp. &c. O. Ky.
969. R. triloba, *Linn.* Aug.–Sept. *y.* 4 to 5 f. ♃. dp. ow. O. Ky. Figured, Bart. Fl. i. 88.
970. R. fulgida, *Ait.* Jul.–Oct. *y.* 2 to 3 f. ♃. Mts. *Bk.* dp. O. Mo. Ter. *Jas.* Figured, Bart. Fl. i. 16.
971. R. laciniata, *Linn.* Cone-flower. Aug.–Sept. *y.* 4 to 6 f. ♃. Borders of swamps. bt. &c. O. Ky. Figured, Bart. Fl. i. 54.
972. R. pinnata, *Michx.* Thimble-flower. Jul.–Oct. *y.* 2 to 3 f. ♃. dp. O. Ky.
973. R. columnaris, *Pursh.* Jul. *r.* 1 f. ♃. On the Missouri. *Pursh.*
974. R. digitata, *Ait.* Aug.–Oct. *y.* 5 to 6 f. ♃. Mountains. *Bk.* Mo. Ter. *Jas.*
975. R. amplexifolia, *Willd.* Aug. *y.* ⊙. Louisville. Rare *M'Murt.*
976. Actinella acaulis, *Linn.* June. *y.* 6 i. *Plt. Jas.*

977. Eclipta procumbens, *Michx.* June. *w.* Decumbent, 1 to 3 f. ⊙? O. Ky. Mo. Ter.

978. E. brachypoda, *Michx.* Aug. Prostrate, 1 to 2 f. ⊙? Near Cincinnati. *Dr. Locke.*

979. Galardia bicolor, *Lam.* June. *p.* 2 f. ♃. Grassy hills, *Plt. Jas.* Ky. *St.*

980. Starkea pinnata, *Nutt.* Aug. *y.* 1 to 2 f. ♃. Plains of the Missouri. Common. *Nutt.* Plt. *Jas.*

981. Trichophyllum oppositifolium, *Nutt.* Jul. 6 to 12 i. ♃? Plt. *Nutt. Jas.*

982. Marshallia angustifolia, *Michx.* Jul. *p.* 2 f. ♃. Tenn. *Michx.*

983. Hymenopappus tenuifolius, *Pursh. g–w.* ♂. Mo. Ter. *Nutt.*

984. Actinomeris squarrosa, *Nutt.* Aug. *y.* 4 to 7 f. ♃. Fence corners, &c. O. Ky. (*w* & *y.* 3 to 4 f. *Bk. Eat. w.* without rays. *Nutt.*) There is misunderstanding here.

985. A. helianthoides, *Eat.* Jul. *y.* 2 f. ♃? ow. dp. Miami Country. W. *Nutt.*

986. Helenium autumnale, *Linn.* American sneezewort. Sept.–Oct. *y.* 2 to 3 f. ♃. Low grounds. W. Tonic errhine. *Eberle,* Mat. Med. ii. 248. Big. Med. Bot.

987. Heliopsis lævis, *Pers.* Ox-eye. Aug.–Sept. *y.* 3 to 5 f. Banks of streams. bt. W.

988. Coreopsis tripteris, *Willd.* Jul.–Sept. *y.* 3 to 6 f. ♃. Borders of prairies. O. Ky.

989. C. trichosperma, *Michx.* Tickseed sunflower. Aug.–Sept. *y.* 2 to 4 f. ♂. Swamps. wp. O. Ky. Mich.

990. C. auriculata, *Willd.* Sept. *y.* 3 to 4 f. ♃. Ky. *St.*

991. C. senifolia, *Willd.* Aug. *y.* 2 to 3 f. ♃. Ky. *St.*

992. C. crassifolia, *Ait.* June. *y.* ♃. St. L. *Jas.*

993. C. palmata, *Nutt. y.* 12 i. Ill. Mich. *Nutt.*

994. C. tinctoria, *Nutt.* Jul. *y* & *p.* 1 to 4 f. Mo. Ter. *Nutt.* About gardens, O. Intr.

995. C. rosea, *Nutt.* Tickweed. May. *r.* 1 f. Grassy swamps. Louisville. *M'Murt.*

996. C. aurea, *Willd. y.* ♃. Louisville. *M'Murt.*

997. C. angustifolia, *Willd. y.* Louisville. *M'Murt.*

998. Bidens frondosa, *Linn.* Burr marygold. Beggar-ticks. Aug.–Sept. *y.* 3 to 4 f. ⊙. Woods and fields. O. Ky. The seeds are said to be alterative.

999. B. bipinnata, *Linn.* Spanish needles. Jul.–Sept. *y.* 1 to 2 f. ⊙. Near cf. O. Ky.

1000. B. chrysanthemoides, *Ell.* Bot. ii. 430. *Michx.* Daisy beggar-ticks. Aug.–Sept. *y.* 1 to 5 f. ⊙. mh. pl. wp. O. Ky.

1001. B. cernua, *Linn.* connata, *Willd?* Aug.–Sept. *y.* 1 to 2 f. ⊙. Near ponds, &c. With 999. O. Ky.
1002. B. comasa, *Hooker.* Ky. *St.*
1003. Verbesina virginica, *Linn.* Virginian crownbeard. Jul. *w.* 3 to 6 f. Louisville, *M'Murt.* Mo. Ter. *Jas.*
1004. Polymnia canadensis, *Linn.* June–Jul. *y.* 2 to 4 f. ♃. sr. sw. &c. O. Ky.
1005. P. uvedalia, *Linn.* Jul.–Sept. *y.* 3 to 5 f. ♃. Mts. *Bk.* Ky. *St.* West Va. *Paddock.*
1006. Silphium ternatum, *Linn.* Jul.–Aug. *y.* 3 to 6 f. ♃. Borders of prairies. dp. O.
1007. S. atropurpureum, *Retz.* Aug.–Sept. *y.* 4 f. Fields. Mar. O. Central O. Rare.
1008. S. perfoliatum, *Linn.* Indian cup-plant. Aug.–Sept. *y.* 4 to 6 f. ♃. Fields and prairies. O. Ky. I believe this was one of the Indian remedies. An account of its virtues and the mode and object of its administration, may be found in Howard's work.
1009. S. terebinthinaceum, *Linn.* Rosin plant. Jul.–Aug. *y.* 4 to 6 f. ♃. dp. O. It is probable that this, and several other species of Silphium will hereafter be found to possess valuable medicinal qualities.
1010. S. gumniferum, *Ell.* Aug. *y.* 3 to 5 f. ♃. Fox Riv. N. W. Ter. *Houghton.* dp. O.
1011. S. laciniatum, *Linn.* Giant prairie dock. Aug.–Sept. *y.* 5 to 10 f. ♃. dp. Darby Plains, O.
1012. S. pinnatifidum, *Ell.* Prairie dock. Aug.–Sept. *y.* 4 to 6 f. ♃. dp. Darby Plains, O. The root is large, and fusiform.—The same is true of several other prairie species, and perhaps of all. The last four species are indiscriminately called *prairie dock*, by the inhabitants of Darby Plains.
1013. Chrysanthemum leucanthemum, *Linn.* Ox-eye daisy. June–Aug. *w* & *y.* 1 to 2 f. ♃. Fields. &c. Intr. O. Asthmatic, &c. Hoop. Dic.

Sub-order VII. Ambrosiaceæ.—Bitter-weed like.

1014. Ambrosia trifida, *Willd.* Bitter-weed. Jul.–Sept. *g* & *y.* 5 to 10 f. ⊙. bt. &c. O. Ky. &c. Every where very abundant.
1015. A. integrifolia, *Willd.* It seems to be an accidental, rather than a permanent variety of the last. Miami country, &c.
1016. A. elatior, *Linn.* Roman wormwood. Ragweed. Hogweed. Aug.–Sept. *g* & *y.* 1 to 5 f. ⊙. Fields. W.

1017. A. artemisifolia, *Linn.* Aug.–Sept. 4 to 6 f. ☉. Fields. Penn. to Mississippi. *Bk.*
1018. A. tomentosa, *Nutt.* 18 i. ♃. Mo. Ter. *Nutt.*
1019. A. bidendata, *Michx.* Jul.–Sept. ☉. Alleghany Mts. W. to Ill. *Bk.*
1020. Xanthium strumarium, *Linn.* Clot-weed. Clot-burr. Aug.–Sept. *g.* 2 to 6 f. ☉. cf. rd. W.
1021. Iva xanthifolia, *Nutt.* Aug. 5 to 6 f. ☉. Arid soils, Fort Mandan, Mo. Ter. *Nutt.*
1022. I. axillaris, *Pursh.* Marsh elder. May. *y* & *g.* 6 to 8 i. ♃. Saline soils, Mo. Riv. *Nutt.*
1023. I. ciliata, *Pursh.* Jul. 2 f. ☉. Natural meadows of Kentucky and Illinois. *Pursh.*
1024. Parthenium integrifolium, *Linn.* Nephritic plant. Cutting almond. Aug.–Sept. *w.* 1 to 2 f. ♃. Near St. L. *Nutt.* The root is by some regarded as a most valuable diuretic in ischuria. Figured and described in the Eclectic Journal of Science, ii. 279.

Sub-order VIII. Anthemideæ.—May-weed like.

1025. Anthemis arvensis, *Linn.* Common chamomile. Aug.–Sept, *w* & *g.* ☉. Waste grounds. Intr. Ky. *St.*
1026. A. cotula, *Linn.* May-weed. Dog fennel. May.–Sept. *w* & *g.* 1 f. ☉. rd. &c. Intr. W. Emetic diaphoretic, antispasmodic. Wood and Bache. Raf. Med. Fl.
1027. Achillea millefolium, *Linn.* Yarrow. Milfoil. Aug.–Sept. *w.* 1 f. ♃. rd. ow. &c. W, Aromatic, bitter, astringent. Hoop. Dic.
1028. Tanacetum huronense, *Nutt.* Huron tansey. *y.* 4 f. ♃. Sandy shores of Lake Huron.
1029. T. vulgare, *Linn.* Tansey. Jul. *y.* 3 f. rd. Intr. O. Aromatic, bitter, anthelmintic. Wood and Bache.
1030. Artemisia canadensis, *Michx.* Wild wormwood. Jul.–Aug. *w* & *y.* 3 to 4 f. ♃. Sandy shores of Lake Erie. Plt. *Jas.* Many species of Artemisia are tonic, anthelmintic, &c. Wood and Bache, 109.
1031. A. longifolia, *Nutt.* ♃ & ♄. rs. Mo. Riv. *Nutt.*
1032. A. serrata, *Nutt.* ♃. 5 f. Banks of Miss. and Mo. Prairie du Chein. *Nutt.*
1033. A. columbiensis, *Nutt.* 6 to 12 f. ♄. Arid saline hills, Mo. Ter. *Nutt.*
1034. A. gnaphalioides, *Nutt.* Sept. Flowers brown. 1 to 2 f. Dry savannahs about Green Bay. *Nutt.*
1035. A. ludoviciana, *Nutt.* 2 f. ♃. St. L. *Nutt.*

1036. A. cernua, *Nutt.* 5 to 8 f. ♃. Shrubby savannahs around St. L. &c. *Nutt.*

1037. A. sericea, *Willd.* A. frigida, *Pursh.* Oct. ♃. Gravelly hills of the Missouri. *Nutt.*

1038. A. biennis, *Willd.* ♂. St. L. Isl. Michill. *Nutt.*

1039. A. cana, *Willd.* Sept. ♃. On the Missouri. *Lewis.*

1040. A. santonica, *Willd.* Tartarian southernwood. Sept. ♃. Plains of the Missouri. *Lewis.* Vermifuge. Wood and Bache, 110.

1041. A. caudata, *Linn.* Sept. 2 f. ♃. Gravelly banks of the Missouri. *Michx.* Nuttall doubts the locality.

### CLXXXIX. *STELLATÆ.*—The madder tribe.

Most of the species seem to possess diuretic qualities.

1042. Galium trifidum, *Linn.* Small cleavers. June–Jul. *w.* Assurgent. ♃. Wet fields, Ky. *St.*

1043. G. tinctorium, *Linn.* Wild madder. Dyer's cleavers. June–Aug. *w.* 1 f. ♃. sw. Wet woods, O. Ky. The root contains a red colouring matter; used in dying.

1044. G. obtusum, *Big.* Jul. *w.* 12 to 18 i. ♃. mh. wp. Wet woods. O. Ky.

1045. G. asprellum, *Michx.* Rough bed-straw. June–Sept. *w.* 1 to 4 f. ♃. O. Ky. Used as a diuretic.

1046. G. triflorum, *Michx.* Jul–Aug. *w.* Procumbent, 3 to 5 f. long. ♃. Moist woods. Ky. *St.*

1047. G. aparine, *Linn.* Goose-grass. Cleaver weed. May–June. *w.* 3 to 4 f. ⊙. Woods, &c. W. Diuretic. Eberle. West. Med. Gaz. Vol. i. Hoop. Dic.

1048. G. micranthum, *Pursh.* Jul. *w.* 10 i. ♃. Mountain swamps. *Pursh.* Woods, O.

1049. G. circæzans, *Michx.* Wild liquorice. June–Jul. *p.* 1 f. ♃. rs. sw. O. Ky. Demulcent, expectorant, diuretic. The root tastes like liquorice.

1050. G. lanceolatum, *Torr.* Jul. *p.* 12 to 18 i. ♃. rs. sw. West Va. *Paddock.*

1051. G. puncticulosum, *Michx.* G. bermudianum, *Pursh.* June. *p.* ♃. Wet places. St. L. *Bk.*

1052. G. boreale, *Pursh.* Aug. *w.* 12 to 18 i. ♃. Sandy woods. Ky. *St.*

1053. G. pilosum, *Ait.* Jul.–Aug. *p.* 1 f. ♃. Woods. Mar. O.

### CXC. *CINCHONACEÆ.*—The cinchona tribe.

Generally possessed of powerfully febrifugal or emetic qualities. Lind. Nat. Syst. 202.

1054. Cephalanthus occidentalis, *Linn.* Button bush. Jul.–

Aug. *w.* 4 to 10 f. ♄. wp. Borders of ponds. W. Medicinal. Raf. Med. Fl. i. Tonic, expectorant. Elliott i. 187.

1055. Spermacoce glabra, *Michx.* June. *w.* ⊙. Ky. *St.*

1056. Mitchella repens, *Linn.* Partridge berry. June–Jul. *w.* Creeping evergreen, 4 to 8 i. long. ♃. rs. sw. W. Berries red, edible. By some it is regarded as expectorant and emenagogue. It is said the Indians used it as a preparatory parturient.

CXCI. *CAPRIFOLIACEÆ.*—The dog-wood tribe.

1057. Lonicera flava, *Sims.* Yellow honey-suckle. June–Jul. *y.* Twining shrub. Cattskill Mts. *Bk.* Darby Plains, O?

1058. L. parviflora, *Lam.* Woodbine. June–Jul. *y.* Twining shrub. rs. Ky. *St.* Mo. Ter. *Nutt. Jas.*

1059. Diervilla canadensis, *Muhl.* Bush honey-suckle. June. *y.* 2 to 3 f. ♄. rs. Diuretic in gonorrhœa. See Hoop. Dic. Art. Lonicera diervilla, *Linn.*, a synonym.

1060. Linnæa borealis, *Gron.* Twin-flower. Jul. *w* or pale *r.* Creeping evergreen. 9 i. high. Woods and hills. Near Lake Huron. *Nutt.*

1061. Triosteum perfoliatum, *Linn.* Fever-wort. Horse gentian. June. *p.* 2 to 3 f. ♃. rs. dp. &c. W. Cathartic, emetic, diuretic. Bart. i. 59. Big. i. 90.

1062. T. angustifolium, *Willd.* Jul. *y.* 2 to 3 f. ♃. Ky. *St.*

1063. Symphoria racemosa, *Pursh.* Snow-berry. Jul. *w–r.* 2 to 3 f. ♄. Rocky hills. Ky. *St.* Mo. *Nutt.* Near Cin. O.

1064. S. glomerata, *Pursh.* Jul.–Aug. *r* & *y.* 3 to 4 f. ♄. Sandy fields. Ky. *St.* Tenn. *Nutt.* Mo. *Jas.*

1065. Viburnum prunifolium, *Linn.* Black haw. June. *w.* 8 to 15 f. ♄. Borders of prairies. Woods. W. Fruit in Oct., sweet and pleasant, dark blue. Bark tonic and astringent.

1066. V. lentago, *Linn.* Sheep berry. June. *w.* 10 to 15 f. ♄. Fruit black. rs. Banks of streams. O. Ky.

1067. V. dentatum, *Linn.* Arrow-wood. June. *w.* 8 f. ♄. Fruit blue. Moist woods. Ky. *St.*

1068. V. pubescens, *Pursh.* June. 6 f. ♄. dp. &c. O. Ky.

1069. V. acerifolium, *Linn.* Dockmackie. May.–June. *w.* 4 to 6 f. ♄. Fruit black. sw. O. Ky. The leaves were used by the Indians as an external application to inflammatory tumours. Eat. Man. Bot.

1070. V. oxycoccus, *Pursh.* High cranberry. May–June. *r–w.* 5 to 8 f. ♄. Fruit red. Banks of streams, &c. Near Lake Sup. *Houghton.*

1071. V. molle, *Michx.* June. ♃. Berries red. Hedges near Danville, Ky. *Michx.* Tenn. &c. *Pursh.*

1072. V. lantanoides, *Michx.* Hobble bush. May–June. *w.* 4 to 8 f. ♄. Fruit black, edible. Mountain woods. Said to grow on the high lands of West. Penn.

1073. Sambucus canadensis, *Linn.* Elder. Sweet elder. June–Jul. 5 to 10 f. ♄. Fruit deep purple. bt. &c. W. Flowers sudorific, berries diaphoretic, aperient, alterative; bark cathartic, emetic. Wood & Bache, 560.

1074. S. pubens, *Michx.* Red elder. May–June. *w.* 6 to 8 f. ♄. Fruit red. Fields, &c. Miami country.

1075. Cornus florida, *Linn.* Dogwood. May–June. *w.* 15 to 20 f. ♄. Fruit scarlet. Woods. W. Excellent tonic. The small twigs make the best of tooth-brushes. The bark of the fibrous roots was used by the Indians in dying scarlet. Bart. Med. Bot. i. 51, 120. Big. ii. 73.

1076. C. sericea, *L'Herit.* Red osier. June. *w.* 5 to 10 f. ♄. Fruit blue. Banks of streams. W. Tonic, astringent. Bart. i. 115. Obviates vomiting. Beach, Mat. Med.

1077. C. circinata, *L'Herit.* June–Jul. *w.* 6 to 8 f. ♄. Fruit blue. Banks of streams. Wor. O. Tonic, astringent. Wood and Bache, 225.

1078. C. paniculata, *L'Herit.* Prairie cornel. Jul. *w.* 6 to 10 f. ♄. Fruit white. wp. Barrens, O.

1079. C. alba, *L'Herit.* Green osier. May–Jul. *w.* 6 to 10 f. ♄. Fruit white. Wet woods. O. Ky. An infusion of the bark of the young twigs, will allay vomiting.

1080. C. alternifolia, *Linn.* June. *w.* 15 to 20 f. ♄. Fruit purple. sw. Germantown, O. *Frank.*

1081. C. asperifolia, *Michx.* Rough-leaved cornel. June. *w.* 4 to 10 f. ♄. Sandy soils. Wheeling, Va.(?)

1082. C. canadensis, *Linn.* Low cornel. May–June. *r–w.* 6 i. ♃. Fruit red. Louisville. *M'Murt.*

1083. Hydrangea vulgaris, *Pursh.* Hydrangea. Jul.–Aug. *w.* 5 f. ♄. Hill sides. rs. O. Ky.

1084. H. cordata, *Pursh.* Heart-leaved hydrangea. June–Jul. *w–r.* 4 to 6 f. ♄. rs. In ravines, &c. O. Ky.

CXCII. *LORANTHEÆ.*—The misletoe tribe.

1085. Viscum verticillatum, *Nutt.* Misletoe. Ap.–May. (*Ell.*) Nov.? *g–w.* 1 to 2 f. ♄. An evergreen parasite, on the elm, the oak, the honey locust, &c., near large rivers. W. Ohio river as far as Marietta. Bark astringent. Lind. Nat. Syst. 206.

CXCV. *ASCLEPIADEÆ.*—The milkweed tribe.

1086. Asclepias syriaca, *Linn.* Silgrass. Milkweed. Jul.–Aug. *w–p.* 2 to 4 f. ♃. rd. bt. W. Medicinal, like 1094. Wood and Bache, 114.

1087. A. obtusifolia, *Michx.* June. Pale *p.* 2 to 3 f. ♃. Sandy fields. Plt. *Jas.* Woods near St. L. *Bk.*

1088. A. phytolaccoides, *Pursh.* Pokeweed silgrass. June.–Jul. *g–p.* 3 to 4 f. ♃. Wet rocky grounds. O. Ky. Medicinal, like 1094.

1089. A. amœna, *Willd.* Jul. 2 to 3 f. ♃. wp, &c. Wor. O. Ky.

1090. A. incarnata, *Linn.* Jul.–Aug. *r–p.* 2 to 4 f. ♃. wp. Banks of streams, O. Ky. Medicinal. See 1094. Wood and Bache, 114.

1091. A. variegata, *Linn.* Jul.–Aug. *w.* 3 to 4 f. ♃. Woods. Ky. *St.* Louisville. *M'Murt.*

1092. A. verticillata, *Linn.* June–Jul. *y–w.* 2 to 3 f. ♃. Dry hills. dp. O. Ky.

1093. A. angustifolia, *Ell.* May. *g–w.* 8 to 18 i. ♃. wp. Scott's Plains, Franklin Co. O.

1094. A. tuberosa, *Linn.* Pleurisy root. Jul.–Aug. *Orange.* 2 to 3 f. ♃. Sandy fields. dp. W. Diaphoretic, expectorant, mild aperient. Big. Med. Bot. ii. 59. Bart. i. 239. Antidysenteric. Eberle's Practice, i. 216.

1095. A. quadrifolia, *Jacq.* June. *w* and *r–w.* 1 to 2 f. ♃. rs. sw. O. Ky. Medicinal, like the last.

1096. Acerates viridiflora, *Raf.* Green milkweed. July. *g.* 2 f. ♃. Sandy fields. Ky. *St.* W. *Bk.*

1097. Podostigma viridis, *Walt.* May. *g* and *p.* ♃. Siliceous hills, Missouri. *Bk.*

1098. Enslenia albida, *Nutt.* Jul. *y–w.* Twining. ♃. rb. bt. &c. O. Ky. The leaves resemble those of a convolvulus.

1099. Gonolobus hirsutus, *Michx.* June–Jul. *p.* Trailing and climbing, 3 to 4 f. ♃. Hedges near streams. (*Bk.*) Ky. *St.*

1100. G. viridiflorus, *Nutt.* *g.* Twining. Banks of the Miss. near St. L. *Nutt.* *Jas.*

1101. G. macrophyllus, *Michx.* June–Aug. *y.* Twining. Light soils. Ky? *St.* The root acts on the bowels in a manner similar to colocynth. Ell. Bot. i. 328.

CXCVI. *APOCYNEÆ.*—The Indian hemp tribe.

1102. Apocynum cannabinum, *Linn.* Indian hemp. June–

Jul. Greenish *w.* 2 to 3 f. ♃. O. Ky. Emetic, cathartic, diuretic, diaphoretic, expectorant. *Knapp*, in Am. Med. Rev. 197. Am. Jour. Med. Sci. for 1833.

1103. A. androsæmifolium, *Linn.* Dog-bane. Wandering milkweed. June–July. Pale *r.* 2 to 4 f. ♃. Fields, &c. O. Emetic, &c. Big. ii. 148. Raf. Med. Fl. i.

1104. A. hypericifolium, *Ait.* St. John's dog-bane. Ju–Jul. Greenish *w.* 1 to 2 f. ♃. rb. Gravelly banks, &c. O. Ky. Medicinal.

1105. A. pubescens, *Brown.* June–Jul. Greenish *w.* 2 to 3 f. ♃. dp. Fields. Wor. O. Medicinal, like 1102, to which it is closely allied.

1106. Amsonia latifolia, *Pursh.* April–May. *b.* 2 f. ♃. Damp soils. Ky. *St.*

### CXCVII. *GENTIANEÆ.*—The gentian tribe.

A bitter quality resides in the stems and roots of these plants, which renders them tonic, stomachic and febrifugal. Lind. Nat. Syst. 214.

1107. Gentiana saponaria, *Linn.* Soapwort gentian. Sept.–Oct. *b.* 1 to 2 f. ♃. wp. O. Ky. Dr. Beck says this is identical with the G. catesbæi of Walter. Tonic. Big. Med. Bot. ii. 127. Figured also, Bart. Fl. N. A. iii. 23.

1108. G. ochroleuca, *Willd.* Aug.–Sept. Yellowish *w.* 1 f. ♃. Sandy fields. Ky. *St.* Brushy Prairie, O. *Van Cleve.*

1109. G. quinqueflora, *Willd.* Five-flowered gentian. Aug.–Sept. *b.* 1 f. ♂. Woods. O. Ky. Tonic. See 1107. Raf. Med. Fl. i.

1110. G. crinata, *Willd.* Fringed gentian. Oct. *b.* 18 i. ♂. wp. O. Tonic. Figured, Bart. Fl. N. A, iii. 27.

1111. G. acuta, *Michx.* Nutt. i. 172. Aug.–Sept. *g–p.* 1 f. ⊙. Plains, Mo. Ter. near Fort Mandan. *Nutt.*

1112. Houstonia cœrulea, *Linn.* Venus' pride. Ap.–June. *b–w.* 4 to 8 i. ♃. ow. &c. O. Ky.

1113. H. longifolia, *Willd.* June. *p.* 6 to 10 i. ♃. dp. Near Middletown, O. Ky. Wet prairies near St. L. *Bk.*

1114. H. purpurea, *Willd.* June–Aug. *p.* 4 to 8 i. ♃. Dry woods. Mar. O. Ky. *St.* Ill. *Bk.*

1115. H. ciliolata, *Torr.* June–Jul. *r–b.* and *w–b.* 2 to 4 i. ♃. rs. sr. Wor. O. St. L. *Bk.* Ky. *St.* Mich.

1116. H. pubescens, *Raf.* May–June, Pale *b.* or *w.* 4 to 8 i. seldom 1 f. ♃. Argillaceous soils, Miami country. West Va.

1117. H. tenuifolia, *Nutt.* 6 i. Dry gravelly hills, Pidgeon river, Tenn. *Nutt.*

1118. H. minima, *Bk.* March. *b.* 1 to 2 i. ⊙? St. L. *Bk.*

1119. Menyanthes trifoliata, *Linn.* Buck-bean. May. *w–r.* 8 to 12 i. ♃. mh. Louisville. *M'Murt.* Tonic, cathartic. Big. iii. 55. Wood & Bache, 415.

1120. Obolaria virginica, *Linn.* Penny-wort. Ap.–May. *w,* or *w–r.* 4 to 6 i. ♃? Woods. Ky. *St.* West Va.

1121. Sabbatia angularis, *Pursh.* American centaury. Aug. *Rose coloured.* 1 to 2 f. ⊙ & ♂. Wet meadows, *Bk.* New dry fields. W. Tonic, stomachic. Big. iii. 147.

1122. S. gracilis, *Salis.* July. *r.* ♂. 1 f. Ky. *St.*

1123. Frasera walteri, *Michx.* American columbo. July. *g–y.* 3 to 6 f. ♂. dp. Barrens. W. Excellent bitter, emetic and cathartic if recent. Bart. ii. 103. If I mistake not, it was first introduced to the notice of the medical profession by Dr. Samuel P. Hildreth, of Marietta, O.

CXCVIII. *SPIGELIACEÆ.*—The pink-root tribe.

1124. Spigelia marilandica, *Linn.* Pink-root. June. *Crimson.* 9 to 18 i. ♃. Woods. Vermifuge, cathartic, narcotic. Bart. ii. 80. Big. i. 143.

CXCIX. *CONVOLVULACEÆ.*—The scammony tribe.

The roots of these plants abound in an acrid milky juice, which is strongly purgative. Lind. Nat. Syst. 216.

1125. Convolvulus arvensis, *Linn.* Bind-weed. June–July. *w.* Prostrate and climbing. ♃. Fields. Ky. *St.*

1126. C. nil, *Linn.* Aug. *w & b.* Twining. ⊙. Intr. O.

1127. C. repens, *Linn.* June. *w,* tinged with *r.* Twining, ♃. bt. mh. Swamps, &c. O. Ky.

1128. C. sepium, *Linn.* (June–July. *w.* Twining, climbing. ♃. Hedges and woods.) *Bk.* Aug. dp. cf. Near Middletown, O.

1129. C. panduratus, *Linn.* Man of the earth. Jul. *w & r.* (*p. Beck.*) Climbing, &c. ♃. Sandy fields. Ky. *St.* Root purgative, diuretic. Bart. i. 252.

1130. C. macrorhizus, *Michx.* Jul.–Aug. (*w,* tinged with *p. Ell.*) *w,* centre *p.* Twining. ♃. Sandy soils. dp. cf. &c. O. Ky. Root large, mildly aperient.

1131. C. spithameus, *Linn.* June. *w.* Generally erect, 1 f. ♃. Sandy woods. Ky. *St.* Near Cin. O. *N. Longworth Esq.*

1132. C. purpureus, *Linn.* Morning glory. Jul.–Aug, *b* or *p.*

Climbing. ⊙. Grows west of the Mississippi. *Eat.* Intr. *Bk. Ell.*

1133. C. tenellus, *Linn.* June–Sept. *w.* Prostrate. ♃. Dry sandy soils, *Ell.* rs. On argillaceous cliffs, two miles north of Worthington, O.

1134. C. micranthus, *mihi.* Small-flowered morning glory. *Root* fusiform and branching, 2 to 4 lines in thickness, flavoured like 1130: *stem* prostrate and twining, 3 to 8 feet long, glabrous: *leaves* broad cordate acuminate, either entire or with one or two lateral teeth or projecting lobes; mealy and subpubescent on both sides, commonly less than 2½ inches long by 2 in width: *petioles* glabrous, 2 to 3 inches long: *peduncles* half as long as the petiole, smooth, with 2 minute subulate bracts near the middle: *flowers* axillary solitary or in pairs: *calyx* 5-leaved or 5-parted, divisions lance-ovate membranaceous slightly awned; two outer sepals ciliate: *corolla* funnel-form, white, near half an inch in length and breadth, twice as long as the sepals. Juice milky. Flowers, Aug.–Sept. ⊙. Fertile woods and prairies, cultivated fields, bt. &c. Miami country. (Closely allied to C. lacunosus, *Spreng.?* perhaps identical with it.)

1135. Evolvulus argenteus, *Pursh.* May. *p.* Erect. 4 to 6 i. ♃. Arid gravelly hills, Mo. Ter. *Nutt.*

1136. Cuscuta americana, *Linn.* American dodder. June–Jul. *w.* Twining parasite, 2 to 3 f. ⊙. Low grounds, W. Yellow and leafless.

CC. *POLEMONIACEÆ.*—The Greek valerian tribe.

1137. Polemonium reptans, *Linn.* Greek valerian. Sweat-root, of Howard. May. *b.* 1 f. ♃. sw. W. The root is said to be an excellent sudorific. See Howard's works.

1138. Phlox paniculata, *Linn.* Lichnidia. June–Jul. *p.* 2 to 3 f. ♃. md. bt. W.

1139. P. pyrimidalis, *Smith.* Aug. *p.* 2 to 3 f. ♃. Mountain meadows, *Bk.* Ky. *St.*

1140. P. maculata, *Linn.* June–Nov. *w–p.* 2 to 3 f. ♃. wp. mh. O. Ky. *St.*

1141. P. aristata, *Michx.* June. *w–p*, or *w.* 18 i. ♃. Wet woods. Ky. *St.* Banks of the Ill. Bk. O. Dr. Beck says the P. pilosa is not distinct from this.

1142. P. divaricata, *Linn.* Called *Sweet-William* in Ohio. Ju. *b.* 9 to 12 i. ♃. (*p.* Woods. W.)

1143. P. reptans, *Michx.* June. *b & p.* 6 to 8 i. ♃. Argillaceous hill sides. O. Ky.
1144. P. subulata, *Linn.* Mountain pink. Ap.–May. *r & p.* Procumbent, 3 to 4 i. high. ♃. Rocky hills, Ky. *St.*
1145. P. setacea, *Linn.* May. *r & p.* Procumbent, ♃. O. Ky.
1146. P. nitida, *Pursh.* June–Aug. *p.* 18 to 24 i. ♃. ow. Near Duncan's Plains, O.
1147. P. cordata, *Ell.* Aug. 1 to 2 f. Ky. *St.*
1148. P. glaberrima, *Linn.* June–Jul. *p.* 1 to 2 f. ♃. Pine barrens, *Ell.* Ky. *St.*
1149. P. bifida, *Bk.* Ap. *p.* 4 to 6 i. Near Fort Clark, Mo. *Bk.*
1150. Collomia linearis, *Nutt.* June. *w–p.* 4 to 12 i. ⊙. Banks of the Missouri, near Shian river. *Nutt.*

### CCI. *HYDROLEACEÆ.*

1151. Diapensia cuneifolia, *Salis.* June. *w.* ♄. Near the Platte river. *Jas.*

### CCII. *EBENACEÆ.*—The ebony tribe.

1152. Diospyros virginiana, *Linn.* Persimmon. May. *g–y.* 20 to 40 f. ♄. Woods. W. Bark bitter, astringent, febrifugal. Wood & Bache, 274. Michx. Sylv. Am. ii. 219. Fruit styptic until matured by frost. It is then rather pleasant.

### CCV. *OLEACEÆ.*—The olive tribe.

1153. Ligustrum vulgare, *Linn.* Prim. Privet. May–June. *w.* 4 to 6 f. ♄. Woods, W. *Bk.*
1154. Chianothus virginica, *Linn.* Fringe tree. May–June. *w.* 6 to 10 f. ♄. Mts. &c. Ky. *St.* O. *Frank.*
1155. Fraxinus acuminata, *Lam.* White ash. May. 50 to 60 f. ♄. Woods. W. Timber light and valuable for many purposes. The bark has much vague celebrity for its reputed medicinal virtues. It is said to be an antidote for snake bites, to remove splenic enlargements, &c.
1156. F. sambucifolia, *Willd.* Black ash. Ap. Large tree. Swamps. O. Ky. It makes most durable rails. The Indians used to make brooms from the saplings.
1157. F. quadrangulata, *Willd.* Blue ash. May. Very large tree. W. Timber valuable; used much in making floors. Bark medicinal.?
1158. F. juglandifolia, *Lam.* Swamp ash. May. A small tree. Central Ohio.

### CCVII. *PRIMULACEÆ.*—The primrose tribe.

1159. Primula farinosa var. americana, *Torr.* Bird's-eye primrose. Pale *p.* 6 to 12 i. ♃. Shores of Lake Huron and Sup. *Nutt. Houghton.*

1160. Androsace occidentalis, *Pursh.* Ap. *w.* 1 to 3 i. ☉. Dry elevated plains, Mo. Ter. *Nutt.* Plt. *Jas.*

1161. Dodecatheon integrifolium, *Michx.* Pride of Ohio. May–June. Pale *b.* 18 i. ♃. Borders of a marsh, 7 miles north of Cincinnati. Yellow Springs, O. Mo. Ter. *Lewis.*

1162. D. meadia, *Linn.* False cowslip. May–June. *p.* 8 to 12 i. ♃. rs. Ky. *St.* Ill. Prairies of St. L. *Bk.*

1163. Hottonia inflata, *Linn.* Water feather. Jul. *w.* (6 to 10 i)? ♃. Stagnant waters. Lagoons of the Licking, Ky. *St.*

1164. Lysimachia ciliata, *Linn.* Money-wort. Jul. *y.* 2 to 3 f. ♃. bt. &c. W. Plants of this genus were formerly reputed to be vulnerary, antiscorbutic, &c. See Boerhaave.

1165. L. stricta, *Ait.* L. racemosa, *Michx.* Upright loose-strife. Jul.–Aug. *y.* 12 to 18 i. ♃. Low grounds. Dayton. O. *Eberle.*

1166. L. quadrifolia, *Linn.* June–Jul. *y.* 12 to 18 i. ♃. Low grounds. O. Ky.

1167. L. revoluta, *Nutt.* Prairie money-wort. July–Aug. Golden *y.* 12 to 18 i. ♃. wp. (rs. *Bk.*) Shores of the great lakes. N. W. Ter. *Hough.* Central Ohio.

1168. L. quadriflora, *Sims.* L. longifolia, *Pursh.* June–Jul. *y.* 18 i. ♃. wp. Ky. *St.* Hoffman's wet grassy prairie, near Dayton, O. (2 to 3 f. Wet woods, *Pursh.*)

1169. L. hybrida, *Michx.* July. *y.* Simple, 2 f. ♃. Grassy prairies, Central Ohio. Ky.

1170. L. heterophylla, *Michx.* June–July. *y.* Branching near the bottom. 12 to 18 i. ♃. Hills, ow. near Cincinnati. Hardly distinct from the preceding, except in habit.

1171. L. capitata, *Pursh.* June. *y.* 1 f. ♃. Swamps, &c. Ohio, shores of Lake Erie. *Nutt.*

1172. L. nummularia, *Linn.* Creeping loose-strife. June–July. ♃. Shady places. (*Hooker.*) Calcareous banks of Lake Michigan. (?) *Nutt.*

1173. Anagallis arvensis, *Linn.* Scarlet pimpernel. Jul. *r.* Procumbent, 4 to 6 i. ☉. Fields. Intr? Ohio. Louisville. *M'Murt.* One of the reputed specifics for hydrophobia. Eberle's Pract. Med. 2d ed. ii. 143.

1174. Centunculus minimus, *Nutt.* Bastard pimpernel. Jul. 4 to 6 i. Margins of ponds, Mo. Ter. *Nutt.*
1175. Samolus valerandi, *Linn.* Water pimpernel. July–Sept. *w.* 6 to 12 i. ♃. Wet grounds. rb. W.

CCVIII. *LENTIBULARIÆ.*—THE BLADDER WORT TRIBE.

1176. Utricularia macrorhiza, *Le Conte.* U. vulgaris, *Pursh.* Bladder wort. Aug.–Oct. *y.* Scape 4 to 8 i. Shoots or roots long, floating in stagnant pools. Lockbourne, Dayton, O. Ky.
1177. U. ceratophylla, *Michx.* Jul.–Aug. Scape 8 i. Root long, floating. ♃. pl. O.?
1178. U. cornuta, *Michx.* Aug. *y.* Scape 10 i. ♃. Wet rocks. Lake Sup. *Hough.*
1179. U. purpurea, *Walt.* Aug. *p.* Scape 2 to 3 i. ♃. Ponds on mountains, *Bk.* N. W. Ter. *Hough.*

CCX. *OROBANCHEÆ.*—THE BROOM-RAPE TRIBE.

1180. Orobanche americana, *Linn.* Cancer-root. Jul. Whole plant brownish yellow. 6 to 8 i. ♃. Parasitic. In patches, on the roots of trees, in sw. W. Antiscirrhous?, astringent.
1181. O. uniflora, *Linn.* Squaw-root. May–Jul. *b–w.* 4 to 6 i. ♃. Parasitic, yellowish white. sw. W. Figured in Bart. Fl. N. A.
1182. Epiphegus virginianus, *Eat.* Beech-drops. Jul.–Sept. *w & p.* 8 to 12 i. ♃. A leafless, brownish yellow parasite, on the roots of the beech tree. W. Astringent, &c. Eber. Mat. Med. i. 355. Antiscirrhous.? Bart. ii. 38.

CCXI. *SCROPHULARINEÆ.*—THE FIGWORT TRIBE.

§ I. *Veroniceæ.* Speedwell like.

1183. Veronica officinalis, *Linn.* Speedwell. June. Pale *b.* 6 to 12 i. ♃. Dry woods, &c. Central Ohio. Alterative, antiscorbutic. Wood & Bache, 661.
1184. V. serpyllifolia, *Linn.* Thyme-leaved speedwell. May–Aug. Pale *b.* Procumbent, 3 to 5 i. br. Wet argillaceous soil. Intr. *Bk.* O. Ky.
1185. V. anagallis, *Linn.* Water speedwell. June–Aug. *b.* 1 to 2 f. ♃. mh. Ditches, &c. O. Ky.
1186. V. scutellata, *Linn.* Scull-cap speedwell. May. Pale *r.* 1 to 2 f. (6 to 12 i. Intr. *Bk.*) ♃. wp. Seemingly indigenous, in the wildest marshes and prairies, O.

1187. V. arvensis, *Linn.* Small speedwell. May. Pale *b.* ⊙. Fields. Intr. O. Ky.
1188. V. agrestis, *Linn.* May. Pale *b.* 3 to 9 i. ⊙. Intr. Sandy fields. O. Ky.
1189. V. peregrina, *Linn.* Wandering speedwell. May–Jul. *w*, or *w–b.* ⊙. Argillaceous soil. O. Ky.
1190. V. reniformis, *Pursh.* June. *b.* Creeping. ♃. Banks of the Missouri, *Pursh.*
1191. Leptandra virginica, *Nutt.* Black-root. Culver's physic. Jul.–Aug. *w*, or pale *r.* 2 to 4 f. ♃. Woods, &c. Cathartic, sudorific, alterative. Wood & Bache, 661. Beach's Mat. Med.

§ 2. *Erinaceæ.*

1192. Buchnera americana, *Linn.* Blue hearts. Jul. *b.* 12 to 18 i. ♃. Sandy places. Prairies near St. L. *Bk.*

§ 3. *Scrophularieæ.*—Figwort like.

1193. Scrophularia marilandica, *Linn.* Figwort. June–July. *Greenish brown.* 3 to 6 f. ♃. bt. &c. W. Like the foreign S. nodosa to which it is closely allied, it would doubtless prove a useful application to scrofulous tumours, hæmorrhoids, &c.
1194. Antirrhinum linaria, *Linn.* Toad-flax. June–Oct. *y* and *orange.* 1 to 2 f. ♃. rd. Intr. O. Ky. Diuretic, cathartic, resolvent. Hoop. Dic.
1195. A. canadense, *Linn.* Flax snap-dragon. June–Aug. *b.* 6 to 12 i. ⊙. Low grounds, Louisville. *M'Murt.*
1196. Mimulus ringens, *Linn.* Monkey flower. Aug. Pale *p.* 2 f. ♃. Wet grounds. W.
1197. M. alatus, *Linn.* Aug. Pale *b.* 2 f. ♃. Wet meadows. bt. br. W.
1198. Gratiola aurea, *Muhl.* Hedge hyssop. Jul.–Aug. *y.* 4 to 8 i. *Eat.* 1 to 2 f. *Bk.* ♃. Sandy swamps, &c. Ohio. *Eberle.* Allied to the foreigh G. officinalis, and like it, doubtless, possesses cathartic, emetic and diuretic properties. See Wood & Bache, 326.
1199. G. virginica, *Linn.* Jul.–Aug. *y–w.* 6 i. ♃. Inundated meadows. O. Ky.
1200. G. missouriana, *Bk.* June. *y.* 4 to 6 i. ♃. Near St. L. *Bk.*
1201. Lindernia attenuata, *Muhl.* Aug. *b.* 6 to 8 i. ⊙. rb. &c. Ky. Central Ohio.
1202. L. dilatata, *Muhl.* Jul.–Aug. Pale *p.* 6 i. ⊙. Inundated banks. W. Fig. Bart. Fl. N. A.
1203. Chelone glabra, *Linn.* Snake-head. Balmony. Aug.–

Oct. *w*, or *r–w*. 2 f. ♃. wp. W. The leaves and flowers are used by the Thompsonians as a tonic. I believe they attribute other properties also to this plant.

1204. Pentstemon pubescens, *Linn.* Beard-tongue. June. Pale *p*. 1 to 2 f. ♃. Hill-sides. W.

1205. P. lævigatum, *Linn.* June. Pale *p*. 1 to 2 f. ♃. bt. ow. Ky. Miami country.

1206. P. gracile, *Nutt.* Slender beard-tongue. June. Pale *p*. Depressed soils. Mo. Ter. *Nutt.* Plt. *Jas.*

1207. P. cristatum, *Nutt.* *p*. 6 to 8 i. ♃. Argillaceous hills. Plt. *Jas.*

1208. P. nuttallii, *Bk.* 2 to 3 f. Near St. L. *Bk.*

1209. P. albidum, *Nutt.* *w*. 6 to 8 i. Plt. *Nutt.*

1210. P. grandiflorum, *Nutt.* 3 f. Prairie du Chien. Plt. *Jas.*

1211. Collinsia verna, *Nutt.* Collinsia. June. *b* & *w*. 8 to 15 i. ☉. bt. &c. O. Ky. Figured, Jour. Acad. Nat. Sci. Philad. i. plate 9. Specimens of this beautiful plant were first communicated to Mr. Nuttall, by Dr. Drake of this city.

1212. Capraria multifida, *Michx.* Jul. *g–w*. 2 to 4 i. ☉. Sandy banks of rivers. W.

1213. Herpestris rotundifolia, *Michx.* Aug. *b*. Procumbent. Margins of ponds. *Ell.* Inundated banks of the Illinois river. *Pursh.*

1214. Gerardia flava, *Linn.* False foxglove. Aug.–Sept. *y*. 2 to 3 f. ♃. Woods. O. Ky. W.

1215. G. glauca, *Eddy.* Aug.–Sept. *y*. 3 to 5 f. ♃. Barrens. dp. W.

1216. G. pedicularia, *Linn.* Louse-wort foxglove. Jul.–Aug. *y*. 2 to 3 f. ♃. Sandy woods or barrens, Mar. O. Ky. N. W. Ter.

1217. G. purpurea, *Linn.* Aug.–Oct. *p*. 1 to 2 f. ☉. (Fields and woods, *Bk.*) Wet prairies. O. Ky. Abundant 8 miles E. of Dayton.

1218. G. tenuifolia, *Linn.* Jul.–Sept. *p*. 6 to 18 i. ☉. cf. dp. Woods, &c. O. Ky.

1219. G. maritima, *Raf.* Jul.–Sept. *p*. 6 to 12 i. ☉. Salt marshes usually. Near Lake Mich. *Hough.*

1220. G. auriculata, *Michx.* Ear-leaf gerardia. Aug.–Sept. *p*, with dark red spots. 2 to 4 f. ☉. Prairie half a mile south of Middletown, O. (8 to 12 i. Rocky fields, *Beck* and others.) Illinois, *Pursh.* Louisville, *M'Murt.*

1221. Seymeria macrophylla, *Nutt.* Mullein foxglove. Jul. *y*. 4 to to 5 f. ♃. Shady alluvial soils, O.

CCXII. *RHINANTHACEÆ.*—THE RATTLE TRIBE.

1222. Pedicularis canadensis, *Linn.* Louse-wort. May–July. *y & p.* 8 to 10 i. ♃. Woods, &c. W.

1223. P. pallida, *Pursh.* Pale-flowered louse-wort. Sept. Pale *y.* 1 to 2 f. ♃. wp. mh. W.

1224. P. gladiata, *Michx.* May–June. *y & p.* 1 f. ♃. Wet meadows. Barrens near St. L. *Bk.*

1225. Melampyrum americanum, *Michx.* Cow wheat. June–July. *y.* 8 to 12 i. ⊙. Woods. Louisville. *M'Murt.*

1226. Orthocarpus luteus, *Nutt.* July–Aug. *y.* 12 to 14 i. Humid situations. Mo. Ter. *Nutt.*

1227. Euchroma coccinea, *Nutt.* Painted-cup. May–June. Corol *y.* Bracts *r.* 8 to 12 i. ♃. Wet grounds. W.

1228. E. grandiflora, *Nutt.* Ap.–May. Corol *g–w.* Bracts *g.* ♃. Prairie du Chien. *Nutt.* Mo. Ter. *Jas.*

CCXIII. *SOLANEÆ.*—THE NIGHT-SHADE TRIBE.

1229. Solanum nigrum, *Linn.* Common night-shade. July–Aug. *w.* 1 to 3 f. ⊙. Old fields. W. Narcotic, emetic, diaphoretic, &c. Eberle's Mat. Med. ii. 75.

1230. S. caroliniense, *Linn.* Horse nettle. June. *w.* 1 f. ♃. Sandy banks. rd. W.

1231. S. dulcamara, *Linn.* Bitter-sweet. Woody night-shade. July–Aug. *p.* Climbing. ♄. Low grounds. W. Narcotic, alterative. Eb. Mat. Med. ii. 77. Big. Med. Bot. i. 169.

1232. S. triflorum, *Nutt.* July. *w.* 1 f. Mo. Ter. *Nutt.* The exotic species S. tuberosum, (potato,) and S. lycopersicum (tomatoes) are every where cultivated.

1233. Physalis obscura, *Michx.* Aug. *y & p.* 1 to 2 f. ⊙. Hills, &c. O. Ky.

1234. P. viscosa, *Linn.* Ground cherry. July–Aug. *y.* 2 to 3 f. ♃. rd. W.

1235. P. pennsylvanica, *Linn.* July–Sept. *y.* 1 f. ♃. Berries red. rd. &c. W.

1236. P. lanceolata, *Linn.* Narrow-leaved ground cherry. July. Pale *y.* 1 to 2 f. ♃. bt. rd. Miami country.

1237. P. pubescens, *Willd.* July. *y.* ⊙. Rocky banks of the Miss. *Bk.*

1238. P. philadelphicum, *Lam.* July. *y & p.* ⊙. West Va. *Paddock.*

1239. Nicandra physaloides, *Pers.* July–Aug. Pale *b.* 2 to 3 f. ⊙. cf. Intr. O. Ky.

1240. Nicotiana rustica, *Linn.* Tobacco. July. *g–y.* 12 to 18 i. *Bk.* ⊙. Intr? Cultivated by the Indians of the Mississippi and Missouri. *Nutt.*

1241. N. tabacum, *Linn.* Virginian tobacco. July. *w–r.* 3 f. ⊙. Intr? Naturalized in Tenn. *Nutt.* Narcotic, emetic, diuretic. Big. ii. 171.
1242. N. quadrivalvis, *Pursh.* July. *b–w.* 1 to 2 f. ⊙. Cultivated by the Western Indians. *Nutt.*
1243. Datura strammonium, *Linn.* Thorn-apple. Jamestown-weed. July–Sept. *b & w.* 2 to 5 f. ⊙. rd. &c. Narcotic. Big. i. 17.
*b.* var. tatula, *Torr.* Stem and flowers purple. W.
1244. D. metel, *Linn.* Aug. *w.* 3 to 4 f. ⊙. Intr. Ohio banks. Dr. Locke informs me that the seed has doubtless been transported by the waters of the river, from Pittsburgh, where the plant was some years since cultivated in a garden.
1245. Hyoscyamus niger, *Linn.* Henbane. June. *y & p.* 12 to 18 i. ⊙. or ♂. Waste places. Naturalized near Detroit. *Eat.* Narcotic, diaphoretic, diuretic, laxative. Big. i. 161.
1246. Androcera lobata, *Nutt.* July–Aug. ⊙. Arid soils. Plt. *Nutt.*
1247. Verbascum thapsus, *Linn.* Mullein. June. *y.* 3 to 6 f. ♂. rd. W. Demulcent, emollient. Woodville Med. Bot. 202. Some practitioners in Ohio, regard it as a valuable diuretic in gonorrhœa.
1248. V. blattaria, *Linn.* Moth mullein. June–July. *w, w & r,* or *y.* 2 f. ♂. rd. bt. W.
1249. V. lychnitis, *Linn.* June–July. Pale *y.* ♂. Louisville. *M'Murt.*

### CCXIV. *ACANTHACEÆ.*—The justicia tribe.

1250. Justicia pedunculosa, *Michx.* Justicia. Water-willow. July–Aug. *r & w.* (Pale *p. Bk.*) 2 f. ♃. rb. In water. W.

1251. J. brachiata, *Pursh.* July. *r.* ♃. Ark. riv. *Jas.*

1252. Ruellia strepens, *Linn.* Herb ruel. July. *b & p.* 1 to 2 f. ♃. bt. ow. W. The leaves are subacrid. Ainslie. ii. 153.

1253. R. oblongifolia, *Michx.* July.–Oct. *b & p.* 8 to 12 i. ♃. dp. Central O.

1254. R. ciliosa, *Pursh.* June-Sept. *w & p.* 3 to 24 i. ♃. Banks of the Miss. *Bk.*

### CCXV. *PEDALINEÆ.*—The oil-seed tribe.

1255. Martynia proboscidea, *Linn.* Unicorn plant. Aug.–Sept. *y & br.* 1 to 2 f. ⊙. River banks. W.

CCXVII. *BIGNONIACEÆ.*—THE TRUMPET-FLOWER TRIBE.

1256. Bignonia radicans, *Linn.* Trumpet-flower. July–Aug. *r.* Creeping on trees, &c. 30 to 70 f. ♄. bt. Banks of streams. W.

1257. B. capreolata, *Michx.* Ap. *r & y.* Climbing over small trees. ♄. Dry soils. Ky. *Eat.*

1258. Catalpa cordifolia, *Ell.* July. *w, y & p.* Sometimes 40 to 50 f. ♄. About old Indian encampments. W. Intr.? Wood durable.

CCXX. *VERBENACEÆ.*—THE VERVAIN TRIBE.

1259. Verbena stricta, *Vent.* Branchless vervain. July–Aug. *b.* 2 to 4 f. ♃. rd. bt. Miami country. W.

1260. V. hastata, *Linn.* Vervain. July–Aug. *b.* 3 to 5 f. ♃. Low grounds. W. Some of the Thompsonians regard this a useful medicinal agent, possessing emetic, cathartic, sudorific and tonic qualities.

1261. V. urticifolia, *Linn.* Nettle-leaf vervain. White vervain. July–Aug. *w.* 2 to 3 f. ♃. rd. ow. W. Said to possess virtues similar to the last.

1262. V. spuria, *Linn.* Aug.–Oct. *b.* Decumbent, 1 to 2 feet long. ⊙. Sandy fields. *Bk.* On slate hills and limestone rocks, Ill. Ky. *Pursh.*

1263. V. angustifolia, *Michx.* June–Aug. *b.* 1 f. ♃. ♃. Prairies near St. Louis mine district. *Bk.*

1264. V. bracteosa, *Michx.* July. *p.* ♃. Ky. *St.* St. L. *Jas.*

1265. V. aubletia, *Linn.* May–Sept. *p.* Assurgent. ♃. Rocky banks of the Miss. *Jas.*

1266. V. paniculata, *Lam.* July–Aug. *p.* 4 to 6 f. Louisville. *M'Murt.*

1267. V. caroliniana, *Linn.* May–July. 2 f. ♃. Louisville. *M'Murt.*

1268. V. bipinnatifida, *Nutt.* June. *b.* ♃. Lead mines of Missouri. *Jas.*

1269. Zapania nodiflora, *Lam.* Fog-fruit. July. *w.* Creeping, 6 to 8 inches long. Peduncle 4 to 6 i. ♃. rb. bt. W.

1270. Z. lanceolata, *Pursh.* July. *w.* Creeping, 6 to 8 i. Peduncle 4 to 6 i. ♃. Banks of streams. Plt. *Jas.*

1271. Z. cuneifolia, *Torr.* Procumbent? Peduncles long. Plt. *Jas.*

1272. Phryma leptostachya, *Linn.* Lopseed. July. *w–p.* 2 to 3 f. ♃. sw. W.

CCXXI. *LABIATÆ.*—THE MINT TRIBE.

"Their tonic, cordial, and stomachic qualities, due to the presence of an aromatic volatile oil and a bitter principle, are the universal feature of Labiatæ, which do not contain a single unwholesome or even suspicious species." Lind. Nat. Syst. 238.

§ I. *Menthoideæ.*—Mint like.

1273. Mentha piperita, *Linn.* Peppermint. July. *p.* 12 to 15 i. ♃. mh. &c. Native? near Worthington, O. Generally Intr. W. Aromatic, stimulant. Wood & Bache, 413.
1274. M. viridis, *Linn.* Spearmint. July–Aug. Pale *p.* 12 to 18 i. ♃. Wet grounds. O. Ky. Aromatic, stimulant, &c. Woodville's Med. Bot. 338. Diuretic?
1275. M. borealis, *Michx.* Northern mint. July–Aug. *w*, or pale *p.* 1 to 2 f. ♃. rb. Moist grounds. O. Ky.
1276. M. tenuis, *Michx.* Slender spearmint. June. *w.* 1 to 2 f. ♃. Low grounds. O. Ky.
1277. M. canadensis, *Linn.* Canadian mint. Aug.–Sept. Pale *p.* 1 f. ♃. Sandy soils. Prairies near St. L. *Bk.*
1378. M. arvensis, *Linn.* Field mint. July. Pale *p.* 1 f. ♃. Naturalized on Duncan's plains. O. (Cornfields. Smells like decayed cheese. *Hooker.*)
1279. Isanthus cœruleus, *Michx.* Isanthus. July–Aug. *b.* 1 f. ⊙. rd. bt. dp. W.
1380. Lycopus virginicus, *Linn.* Bugle weed. June–Aug. *w.* 1 to 2 f. ♃. Wet places. rb. O. Ky. Mild narcotic, astringent, &c. Used to allay hæmoptysis. N. York Med. and Phys. Jour. i. 179.
1281. L. europæus, *Linn.* Water horehound. Aug. *w.* 1 to 2 f. ♃. Wet places. Astringent, &c.

§ 2. *Satureineæ.*—Rosemary like.

1282. Pycnanthemum incanum, *Michx.* Wild basil. July–Sept. Pale *r.* 2 to 3 f. ♃. Low fields. Mar. O. Ky.
1283. P. aristatum, *Michx.* July–Aug. *w.* 1 to 2 f. ♃. Woods. Tenn. *Bk.* Louisville. *M'Murt.* O. Ky. *Nutt.*
1284. P. lanceolatum, *Pursh.* July–Aug. *w & p.* 2 f. ♃. dp. O. Ky. Abundant on prairies up the Miami river.
1285. P. linifolium, *Pursh.* Virginian thyme. July–Aug. *w.* 1 to f. ♃. Prairies and woods. O. Ky. This fragrant plant is used medicinally by the inhabitants of Sandusky plains, O.
1286. P. pilosum, *Nutt.* June. 18 to 24 i. Prairies near St. L. *Bk.* Glades of Ky. and Tenn. *Nutt.*
1287. Thymus serpyllum, *Linn.* Wild thyme. July. *p* or *w.* Spreading. ♃. Naturalized in a few localities, O.
1288. Origanum vulgare, *Linn.* Marjoram. July–Sept. *w–p.*

8 to 12 i. ♃. Louisville. *M'Murt.* Tonic, excitant, diaphoretic, emenagogue. Wood & Bache, 473.

1289. Hyssopus nepetoides, *Linn.* Giant hyssop. July. *y–w,* or *w–p.* 3 to 6 f. ♃. ow. W.

1290. H. scrophularifolius, *Linn.* July–Aug. *p.* 2 to 4 f. ♃. O. Ill.

§ 3. *Ajugoideæ.*—Germander like.

1291. Teucrium canadense, *Linn.* Wild germander. July–Aug. *w & r.* 2 to 3 f. ♃. Thickets, low grounds. W.

1292. T. virginicum, *Linn.* Aug. ♃. Low grounds. W. *Bk.*

1293. Trichostemma dichotoma, *Linn.* Blue curls. June–Aug. *b.* 6 to 12 i. ⊙. Dry hills. Ky. *St.* W. *Bk.*

1294. Collinsonia canadensis, *Linn.* Horse-balm. Rich-weed. July–Sept. *y.* 2 to 3 f. ♃. sw. The root is by some regarded as diuretic and antilithic. There is a notion extant that it will relieve after pains. See Raf. Med. Fl. i.

§ 4. *Monardeæ*

1295. Monarda coccinea, *Michx.* Wild balm. Oswego tea. July–Aug. *Crimson.* 2 to 3 f. ♃. Swamps. Cultivated in Ohio, idigenous east of the Allegh. Mts.

1296. M. allophylla, *Michx.* Mountain balm. July. Pale *b.* 3 to 4 f. ♃. Rocky woods. Ky. *St.*

1297. M. fistulosa, *Linn.* July–Aug. Pale *y.* 2 f. ♃. Rocky banks, &c. O. Ky. Nervine, stomachic, deobstruent. Hoop. Dic.

1298. M. clinopodia, *Linn.* July–Sept. Pale *p.* 3 f. ♃. Woods. O.

1299. M. punctata, *Linn.* Horse-mint. Sept. *y* and *brown.* 2 to 3 f. ♂. and ♃. Pine barrens. Mo. Ter. *Jas.* W. *Bk.* Stimulant, carminiative, rubefacient. Ame. Med. Rec. ii. 496. Raf. Med. Bot. ii. 38.

1300. M. hirsuta, *Pursh.* Ohio horse-mint. June–July. Pale *b.* and *p.* 2 to 3 f. ♃. Low woods. bt. W. The properties are said to be similar to the last.

1301. M. Beckii, *Eat.* Near St. L. *Bk.*

1302. M. ciliata, *Linn.* Aug. *p.* ♃. Mich. *Eat.* Ky. *St.* Mo. Ter.

1303. M rugosa, *Ait.* July. *w.* 4 f. ♃. Pittsburgh, *Peter.*

1304. M. scabra, *Bk.* Aug. *p.* 3 f. ♃. Woods on the banks of the Miss. *Bk.*

1305. M. bradburiana, *Bk.* July. *w & p.* 2 to 3 f. ♃. Barrens N. of St. L. *Bk.* Miami country. Duncan's plains, O.

1306. Cunila mariana, *Linn.* Dittany. July–Aug. Pale *r.* 1

to 2 f. ♃. Rocky hills. Mar. O. Ky. Pleasant aromatic. For its medicinal virtues, see Raf. Med. Bot.

1307. C. glabella, *Michx.* Aug. *p.* 8 to 10 i. ♃. Among limestone rocks. Frankfort, Ky. *St.* W. *Bk.*

1308. Tullia pycnanthemoides, *Leavenworth. Eat.* Aug. *r–p.* 2 to 3 f. ♃. Tenn. *Leavenworth.*

§ 5. *Nepeteæ.*—Catmint like.

1309. Galeopsis tetrahit, *Linn.* Hemp nettle. Flowering nettle. July. *r, w & p.* 1 to 2 f. ☉. Waste places. Mich. *Houghton.*

1310. Leonurus cardiaca, *Linn.* Motherwort. July–Aug. *w & r.* 2 to 3 f. ♃. rd. &c. Intr. O. Ky. Said to promote uterine discharges, and allay palpitation of the heart. Hoop. Dic.

1311. Lamium amplexicaule, *Linn.* Dead nettle. Hen-bit. May–Nov. *r.* 6 to 8 i. ☉. rd. O. Ky. Intr?

1312. L. hispidulum, *Michx.* *w.* Shady woods of Tenn. *Michx.* Louisville. *M'Murt.*

1313. Dracocephalum virginianum, *Linn.* Dragon-head. June?–Sept. *p.* 1 to 2 f. ♃. Prairies. W.

1314. D. variegatum, *Vent.* June?–Aug. *p & w.* 3 f. ♃. wp. Duncan's plains, O. Ky.

1315. D. cordatum, *Nutt.* June. *b.* 1 f. ♃. On the shady islands of the Ohio, 40 miles below Pittsburgh.

1316. D. parviflorum, *Nutt.* July. *w.* ♂. Mo. Ter. *Nutt.*

1317. Nepeta cataria, *Linn.* Catmint. June–Sept. *w & r.* 2 to 3 f. rd. &c. Intr. W. Recommended in uterine diseases, dyspepsia, flatulency, &c. Hoop. Dic. A favourite remedy for colds in domestic practice.

1318. Synandra grandiflora, *Nutt.* June. *y–w.* 1 f. ♃? rs. sw. O. Ky. &c.

1319. Stachys aspera, *Michx.* Hedge nettle. July. *p.* 1 f. ♃. Fields, &c. W.

1320. S. sylvatica, *Nutt.* Aug. *p & w.* 1 to 2 f. ♃. sw. W.

1321. S. intermedia, *Ait.* (Aug. *w & r.* 1 to 2 f. ♃. Woods. O.)?

1322. Glechoma hederacea, *Linn.* Ground ivy. May–June. *b.* Decumbent, 1 f. rd. ow. O. Ky. A tonic beverage is often made from it. Lind. Nat. Syst. 238.

1323. Marrubium vulgare, *Linn.* Horehound. July–Aug. *w.* 12 to 18 i. ♃. rd. W. Tonic, deobstruent. Wood & Bache, 409.

1324. Mellissa officinalis, *Linn.* Balm. July. *b.* ♃. rd. Intr. and naturalized near Louisville. *M'Murt.* Formerly used in nervous diseases. Hoop. Dic.

1325. Hedeoma pulegioides, *Pers.* Pennyroyal. July–Aug. *w–p.* 6 to 10 i. ⊙. ow. rd. &c. W. Stimulant, aromatic, emenagogue. Bart. ii. 168.

1326. H. glabra, *Pers.* Aug. (*r–p.*)? 6 to 12 i. ♃. Ohio riv. *Nutt.* Near Wor. O.

1327. H. hispida, *Pursh.* July. 3 to 6 i. ⊙. Banks of the Miss. *Bk.* Mo. Ter. *Nutt.*

1328. Clinopodium vulgare, *Linn.* Field thyme. July–Aug. *p*, or *r.* 12 to 18 i. ♃. Rocky woods. W. *Bk.* Thickets near Mar. O.

1329. Prunella vulgaris, *Linn.* Self-heal. June–Aug. *p.* 8 to 12 i. ♃. md. W. Astringent, &c. Used as a gargle. Hoop. Dic.

*Scutellaria.*—Scull-cap. The western species are probably all good diaphoretics.

1330. Scutellaria galericulata, *Linn.* Aug. *b.* 12 to 18 i. ♃. Near marshes. Ky. *St.* See Hoop. Dic.

1331. S. lateriflora, *Linn.* Mad dog scull-cap. July–Aug. *b.* 1 to 2 f. ♃. md. ow. wp. W. Famous some years since for its supposed efficacy in hydrophobia.

1332. S. parvula, *Michx.* June. *b.* 3 to 6 i. ♃. Ky. *St.*

1333. S. lævigata, *Aikin.* May. *b.* 12 to 18 i. ♃. Mar. O?

1334. S. integrifolia, *Linn.* June. *b.* 18 to 24 i. ♃. Swamps. W. *Bk.*

1335. S. cordifolia, *Michx.* June. *b & w.* 18 to 30 i. ♃. Woods. W.

1336. S. canescens, *Nutt.* July. *b.* 2 to 3 f. ♃. Woods. W. Prairies near St. L. *Bk.* Ky. *St.* O. *Nutt.*

1337. S. gracilis, *Nutt.* June. *b.* 12 to 18 i. ♃. Shady rocks. Ky. *St.*

1338. S. ovalifolia, *Pers.* June. *b.* Assurgent, 1 f. ♃. Woods. W.

1339. S. ambigua, *Nutt.* Pale *b.* 4 to 6 i. ♃. Dry open forrests, O. *Nutt.* Ky. *St.*

1340. Salvia claytoni, *Ell.* Vervain sage. June–Oct. *p.* 8 to 12 i. ♃. Woods. Ky. *Eat.*

1341. S. lyrata, *Linn.* Wild sage. May–June. *b.* 1 f. ♃. Woods. West Va. *Padd.* Ky. *St.*
*b.* var. obovata, *Eat.* rb. Cincinnati.

1342. S. trichostemmoides, *Pursh.* *b.* ⊙. Mo. Ter. *Jas.*

### CCXXII. *BORAGINEÆ.*—The borage tribe.

Generally possess emollient properties. Lind. 239.

1343. Lithospermum arvense, *Linn.* Corn gromwell. Stoneseed. Ap.–May. *w.* 8 to 15 i. Fields, Ky. *St.* Formerly supposed to be diuretic and lithontriptic.

1344. L. latifolium, *Michx.* Broad-leaf gromwell. July. *y.* 2 f. ♃. sw. O. Ky.
1345. L. angustifolium, *Michx.* July. *w.* ⊙. Shady woods on the Ohio river. *Michx.* Council Bluffs, Mo. Ter. *Jas.*
1346. L. apulum, *Willd.* July. *y.* Dry woods, Ohio, Miss., &c. *Pursh.*
1347. Batschia canescens, *Michx.* Puccoon. June–July. *Orange.* 8 to 12 i. ♃. Hills, &c. W. Used by the Indians as a red dye.
1348. B. longiflora, *Nutt.* July. *y.* Open plains. Prairie du Chien. *Nutt.*
1349. Onosmodium hispidum, *Michx.* Hairy gromwell. July–Aug. *w.* 2 to 3 f. ♃. Borders of prairies. W.
1350. O. molle, *Michx.* July–Aug. *w.* 2 f. ♃. W.
1351. Symphitum officinale, *Linn.* Comfrey. June. *y–w.* 1 to 3 f. ♃. Intr. O. Hardly naturalized. Demulcent. Hoop. Dic.
1352. Lycopsis arvensis, *Linn.* Small bugloss. June–July. *b.* 12 to 18 i. ⊙. Sandy fields. Intr? Prairies near St. Charles. Mo. *Bk.*
1353. Myosotis arvensis, *Sibth.* Field scorpion grass. June. *w.* 4 to 10 i. ⊙. Sandy woods. Ohio. *Eberle.* Ky. *St.* St. L. *Bk.*
1354. M. palustris, *Roth.* Marsh scorpion grass. May–July. *b.* 12 to 18 i. ♃. mh. Ditches, &c. Plt. *Jas.* Pittsburgh. *Peter.*
1355. M. suffruticosa, *Torr.* *b?* 8 i. Plt. *Jas.*
1356. Echinospermum virginicum, *Lehm.* Rochelia virginiana, *Torr.* Beggar-lice. July–Sept. Pale *b* or *w.* 2 to 4 f. ♂. ow. W.
1357. Cynoglossum officinale, *Linn.* Hound's tongue. June–July. *r.* 2 to 3 f. ♂. rd. W. Intr. Narcotic and demulcent. Hoop. Dic.
1358. C. amplexicaule, *Michx.* Wild comfrey. May. *b.* 2 to 3 f. ♃. sw. W.
1359. C. pilosum, *Linn?* May. *w.* Arid hills, above Rapid river, Mo. Ter. *Nutt.*
1360. Pulmonaria virginica, *Linn.* Lungwort. May. *b.* 6 to 12, and 24 i. ♃. Low grounds. W. Astringent, demulcent, and pectoral. See Raf. Med. Fl.
1361. P. lanceolata, *Pursh.* June. *b.* ♃. Mo. Ter. *Nutt.*

CCXXIII. *HELIOTROPIACEÆ.*—The heliotrope tribe.

1362. Heliotropum indicum, *Linn.* Turnsole. Heliotrope. July. *b.* 8 to 12 i. ⊙. Banks of streams. Cin. O. Ky.

1363. H. curassavicum, *Linn.* June–July. *w & y.* 6 to 12 i. ⊙? Plt. *Jas.*

### CCXXVI. *HYDROPHYLLEÆ.*—The water-leaf tribe.

1364. Hydrophyllum virginicum, *Linn.* Water-leaf. June. *w & b.* 18 i. ♃. bt. sw. rs. W. Used as a potherb in New York, under the name of John's cabbage.

1365. H. canadense, *Linn.* Burr-flower. June. *b & w.* 18 i. ♃. Argillaceous soil. Woods, &c. O. Ky.

1366. H. lineare, *Pursh.* Ap. ♃. Banks of the Missouri. *Lewis.*

1367. H. hispidum, *mihi.* Perennial?, whole plant hispid or setose with terete, pointed white hairs: *leaves* mostly radical, pinnatifid, lower lobes often isolated; lobes obovate, border divided by shallow gashes into 5 to 8 rounded, ciliate, entire, mucronate lobuli, which together present a palmated appearance; general outline of the leaf oblong or oval, (4 to 8 inches long, 2 to 3 in width,) on petioles of equal length (4 to 8 inches). The single cauline leaf does not vary essentially from the above description. It is broader in proportion to its length, and its petiole is much shorter. *Peduncle* exceeding the leaves, 8 to 12 inches in height. *Flowers* in cymose clusters as in H. virginicum; divisions of the calyx broad subulate, pectinately setose; corol campanulate, border with 5 rounded lobes; filaments densely bearded in the middle, double the length of the corol; style filiform, bifurcate to the extent of a line; capsule 2-valved (1-celled, 2 to 4-seeded.)? This description has been taken from dried specimens. Flowers in June. Woods, O.

1368. Nemophila paniculata, *Spreng.* May. *b.* 1 f. ⚩. Woods. O. Ky.

1369. Phacelia fimbriata, *Michx.* Miami mist. May–June. *b.* 8 to 12 i. ⊙? Fields and bottom lands. W.

1370. P. bipinnatifida, *Michx.* Phacelia. May. *b.* 1 f. ♃? Hill sides. Ky. Cin. O.

1371. P. heterophylla, *Pursh.* Various-leaved phacelia. July. *b.* ⚩. Ky. *St.*

1372. P. integrifolia, *Torr.* Plt. *Jas.*

1373. Ellisia nyctelea, *Linn.* June. *w & b.* Decumbent. 6 to 8 i. ⊙. Mo. Ter. *Jas.*

1374. E. ambigua, *Nutt.* May. *b.* Decumbent. 4 to 6 i. ⊙. Lead mines, Mo. *Jas.*

Tribe II. Gymnospermæ.—*Seeds destitute of a pericarp.*

### CCXXVIII. *CONIFERÆ.*—The fir tribe.

1375. Juniperus communis, *Linn.* Juniper. May. An evergreen shrub. Rocky banks of streams. Sandy shores of Lake Huron. *Nutt.* Berries stimulant and diuretic. Woodville's Med. Bot. 13. Big. iii. 44.

1376. J. prostrata, *Michx.* May. A low evergreen shrub, with creeping branches, 6 feet long. Sandy shores of Lake Huron. *Nutt.*

1377. J. virginiana, *Linn.* Red cedar. May. Middle-sized evergreen tree. Woods, rocky banks of streams, W. Secernant stimulant, emenagogue, diuretic, diaphoretic. Big. ii. 49. Timber durable.

1378. J. sabina, *Willd.* Savin. May. ♄. Grows near Lake Superior. *Pitcher.* Properties like the last. Wood & Bache, 548.

1379. Taxus canadensis, *Willd.* Dwarf yew. March–April. 2 to 6 f. ♄. Evergreen. Fruit red. rs. &c. Louisville. *M'Murt.*

1380. Cupressus thyoides, *Linn.* White cedar. May. An evergreen tree, 30 to 50 f. Swamps. rs. Rocky banks of the Scioto. Wood durable.

1381. C. disticha, *Linn.* Cypress. May. Large evergreen tree. Swamps. Ky. *St.* W. *Bk.* Timber very durable.

*Pinus.*—The pine family.

* *Leaves in fascicles of 2 to 5, persistent, sheathing at base; scales of the cone thickened at the summit.* Pinus.

1382. Pinus inops, *Ait.* Scrub pine. Pitch pine. Jersey pine. May. Common-sized tree. Barrens, &c. Ky. *St.*

1383. P. variabilis, *Lamb.* Yellow pine. May. Large tree. Forests, on hills about Portsmouth, O. (?)

1384. P. banksiana, *Lamb.* Grey pine. Scrub pine. April–May. Small tree. rs. Mar. O. Rare.

1385. P. strobus, *Linn.* White pine. Weymouth pine. May. Large tree, 90 to 140 f. Fertile soils. East Ohio. Timber valuable.

* * *Leaves solitary, persistent, distinct at base; scales of the cone even and attenuated.* Abies.

1386. P. canadensis, *Linn.* Hemlock tree. Hemlock spruce. May. Large tree. Woods. O. Scarce. Bark astrin-detergent; used in tanning leather. A fomentation of the boughs is highly esteemed in domestic practice for its efficacy in subduing colds. Turpentine like that of all other species of Coniferæ stimulant, rubefacient, and diuretic.

1387. P. nigra, *Ait.* Black spruce. May. Large pyramidal tree. Swamps. Mich. *Eat.*

1388. P. alba, *Ait.* White spruce. May. Small tree. Swamps. Ky. *St.*

* * * *Leaves many in a fascicle, deciduous.* LARIX.

1389. P. pendula, *Ait.* Tamarack. Hackmatack. Ap.–May. Middle-sized tree. Swamps. East Ohio. Bark tonic and diuretic, very highly esteemed by some practitioners.

### *CLASS II.—ENDOGENÆ.*

TRIBE I. PETALOIDEÆ.—*Calyx and corolla developed in 3 or 6 divisions; if absent, stamens and pistils naked.*

### CCXXIX. *ALISMACEÆ.*—THE WATER PLANTAIN TRIBE.

1390. Alisma plantago, *Linn.* Water plantain. July–Aug. *w.* 1 to 2 f. ♃. mh. pl. W. One of the reputed remedies for hydrophobia. Lind. 251.

1391. Sagittaria sagittifolia, *Willd.* Arrow-head. Arrow-leaf. July–Aug. *w.* 1 to 2 f. ♃. mh. pl. O. Ky.
*b.* var. latifolia, *Torr.* Leaves broad, rather obtuse. O.
*c.* var. hastata, *Torr.* Leaves subhastate. O.
*d.* var. gracilis, *Torr.* Leaves linear hastate. Dayton, O.

1392. S. obtusa, *Willd.* Blunt arrow-leaf. July. *w.* 1 to 2 f. ♃. mh. wp. Ditches, &c. O.

1393. S. pusilla, *Nutt.* Aug. 2 to 4 i. ♃. Muddy banks. Ky. *St.*

### CCXXXI. *HYDROCHARIDEÆ.*—THE FROG-BIT TRIBE.

1394. Udora canadensis, *Nutt.* Ditch moss. Aug. *w.* Submersed, 1 to 2 f. ♃. pl. Ohio. Ky.

1395. Vallisneria spiralis, var. americana, *Torr.* Tape grass. Aug. *w.* 1 to 4 f. ♃. In the still waters of rivers. W.

### CCXXXII. *COMMELINEÆ.*—THE SPIDER-WORT TRIBE.

1396. Commelina communis, *Linn.* Commelina. Aug. *b.* ☉. Ky. *St.*

1397. C. virginica, *Linn.* July. *b.* 2 f. ♃. Woods. Ky. *St.*

1398. Tradescantia virginica, *Linn.* Spider-wort. May–July. *p.* 1 f. ♃. sw. dp. O. Ky. Fig. Bart. Fl. N. A. ii. 17.

1399. T. rosea, *Michx.* May. *r.* 8 to 12 i. ♃. Moist woods. Ky. *St.*

CCXXXIII. *XYRIDEÆ.*—The yellow-eyed grass tribe.

1400. Xyris caroliniana, *Walt.* Yellow-eyed grass. July. *y.* 1 f. ♃. wp. mh. md. O. Ky.

CCXXXIV. *BROMELIACEÆ.*—The pine-apple tribe.

1401, Agave virginica, *Linn.* False aloe. Sept. *g–y.* 6 f. ♃. Rocky banks. Ky. *St.*

CCXXXV. *HYPOXIDEÆ.*—The star-grass tribe.

1402. Hypoxis erecta, *Linn.* Star-grass. June–July. *y.* 4 to 6 i. ♃. md. rs. ow. O. Ky. Figured, Bart. Fl. N. A. i. 124.

CCXXXVIII. *AMARYLLIDEÆ.*—The narcissus tribe.

1403. Pancratium rotatum, *Ker.* July. *w.* ♃. Ky. *St.*
1404. Amaryllis atamasco, *Linn.* Atamasco lily. June. *w & r.* Scape 6 i. Leaves 1 f. sw. Louisville. *M'Murt.* Said to grow in Indiana.

CCXXXIX. *IRIDEÆ.*—The iris tribe.

1405. Iris versicolor, *Linn.* Blue flag. June. *b,g & y.* 2 to 3 f. ♃. Margins of ponds, &c. O. Ky. Cathartic, emetic, diuretic. Big. i 155. By some said to be alterative.
1406. I. cristata, *Michx.* April. *b & y.* 2 to 4 i. ♃. Ky. *St.*
1407. I. verna, *Willd.* May. *b.* ♃. Ky. *St.*
1408. I. lacustris, *Nutt.* June. Scape 1 i. Leaves 3 to 5 i. ♃. Gravelly shores of Lake Huron. *Nutt.*
1409. Sisyrinchium anceps, *Linn.* Blue-eyed grass. July. *b.* 1 f. ♃. Pastures, &c. O. Ky. The Thompsonians use it as a cathartic, under the name of *physic-grass.*
1410. S. mucronatum, *Michx.* July. *b.* 6 to 10 i. ♃. Wet meadows. Ky. *St.* Plt. *Jas.*
1411. S. bermudianum, *Linn.* June. *b.* 8 to 18 i. ♃. Ill. *Bk.*

CCXL. *ORCHIDEÆ.*—The orchis tribe.

1412. Goodyera pubescens, *Brown.* Rattlesnake plantain. July–Aug. *w.* 6 to 10 i. ♃. sw. O. Ky.
1413. Spiranthes tortilis, *Rich.* Ladies' tresses. Aug.–Oct. (June–July. *Bk.*) *w.* 8 to 12 i. ♃. wp. O. Ky. Fig. Bart. Fl. N. A. i. 124.
1414. S. cernua, *Rich.* July–Aug. *g–w.* 6 i. to 2 f. ♃. Moist grounds. West Va. *Paddock.*
1415. Pogonia ophioglossoides, *Brown.* July. Pale *p.* 8 to 12i. ♃. bg. Sphagnous swamps. Ky. *St.* Fig. Bart. Fl. N. A. iii. 19.

1416. P. verticillata, *Nutt.* June–July. *y–r.* 1 f. ♃. Swamps. Ky. *St.* Mich. *Eat.* Fig. Bart. Fl. N. A. ii. 92.
1417. Callopogon pulchellus, *Brown.* Grass pink. June–July. *p.* 12 to 18 i. ♃. wp. Swamps. Ky. *St.* Pickaway plains, O. Fig. Bart. Fl. N. A. ii. 94.
1418. Corallorhiza odontorhiza, *Nutt.* Dragon's claw. Aug.–Sept. *Purplish.* 10 to 12 i. ♃. Roots of trees. O. Ky.
1419. Aplectrum hiemalis, *Nutt.* Adam and Eve. May–June. *Brownish.* 1 f. ♃. sw. bt. O. Ky. Fig. Bart. Fl. N. A. ii. 42. Root viscid.
1420. Triphora pendula, *Nutt.* Three-bird orchis. Sept. Pale *p.* 3 to 6 i. ♃. sw. Roots of trees. O. Ky.
1421. Orchis spectabilis, *Linn.* Orchis. June. *p & w.* 6 to 8 i. ♃. sw. O. Ky.
1422. Habenaria tridentata, *Hook.* June–July. *w.* 1 to 2 f.
1423. H. ciliaris, *Brown.* June–July. *Orange y.* 1 to 2 f. ♃. Swamps. Ky. *St.*
1424. H. huronensis, *Spreng.* Aug. ♃. Wet grounds. About Lakes Mich. and Huron. *Bk.*
1425. H. fimbriata, *Brown.* July. *p.* 2 f. ♃. md. wp. O. Ky.
1426. H. fissa, *Brown.* July. Dark *p.* 2 to 4 f. ♃. Prairies. O. Ky.
1427. Platanthera orbiculata, *Lind.* July. *g–w.* 12 to 18 i. ♃. sw. Central, O. I have known the fleshy leaves of this plant, to be used and highly esteemed for dressing blisters.
1428. Liparis liliifolia, *Rich.* Malaxis, *Eat.* Twayblade. June–July. *w & y.* 6 to 8 i. ♃. Wet woods. Central, O.
1429. L. correana, *Spreng.* Malaxis longifolia, *Eat.* June. *y–g.* 6 to 8 i. ♃. sw. O. Fig. Bart. Fl. N. A. iii. 12.
1430. Microstylis ophioglossoides, *Nutt.* June. *g–w.* 6 to 9 i. ♃. Roots of trees. O. Ky. Fig. Bart. Fl. N. A. iii. 60.
1431. Bletia aphylla, *Nutt.* Aug. *y & r.* 18 i. ♃. Ky. *St.*
1432. Calypso americana, *Brown.* *p.* 6 to 8 i. Near the outlet of Lake Michigan. *Bk.*
1433. Cypripedium pubescens, *Swartz.* Ladies' slipper. May. *g–y.* 1 to 3 f. ♃. Woods. O. Ky. Fig. Bart. Fl. N. A. iii. 9.
1434. C. parviflorum, *Willd.* Yellow ladies' slipper. May–June. *y.* 1 f. ♃. Dry woods. W. Said to be excellent as a nervine, tonic, and antispasmodic. The powder of the root is administered in tea spoonful doses, for hysterical and other affections. The Thompsonians, by whom it is used, call it *umbil* and *wild valerian.* They use all the species of Cypripedium indiscriminately. See Raf. Med. Fl.

1435. C. candidum, *Willd.* White ladies' slipper. May. *w.* 6 i. ♃. Dayton, O. *R. Buchanan, Esq.*

1436. C. spectabile, *Swartz.* Swamp ladies' slipper. May–June. *w & p.* 2 to 3 f. ♃. mh. bg. W. Ky. *St.* Mich. *Eat.* Seven miles N. from Cin. O. Fig. Bart. Fl. ii. 87.

1437. C. acaule, *Ait.* May–June. *p.* 1 f. ♃. sw. Ky. *St.* Mich. *Eat.* Fig. Bart. Fl. iii. 35.

### CCXLIV. *JUNCEÆ.*—The rush tribe.

### *Juncus.*—Rush.

* *Leaves none, flowers lateral.*

1438. Juncus effusus, *Linn.* Soft rush. June–July. 2 to 3 f. ♃. Wet grounds. Ky. *St.* Central Ohio.

1439. J. setaceus, *Rostk.* Thread-form rush. July. 2 to 3 f. ♃. Swamps. Miami country.?

* * *Leaves all radical, flowers terminal.*

1440. J. nodosus, *Linn.* Knot-leaf rush. July–Aug. 8 to 12 i. ♃. Swamps. Low grounds. St. Mary's river. *Hough.* Cin. O.

1441. J. tenuis, *Willd.* Slender spreading rush. June–July. 10 i. ♃. Low grounds. O. Ky.

* * * *Stem leafy; leaves nearly plane, channelled above.*

1442. J. bufonius, *Linn.* Toad rush. Aug. 3 to 6 i. ☉. Moist places. Miami country.?

1443. J. marginatus, *Rostk.* Aug. 1 to 3 f. ♃. Low grounds. Miami country.?

* * * * *Stems leafy; leaves rounded or subcompressed, nodose-articulate.*

1444. J. polycephalus, *Michx.* Many-headed rush. July–Aug. 2 to 3 f. ♃. mh. &c. Worthington, O. Miami country. N. W. Ter.

1445. J. acuminatus, *Michx.* July. 12 to 18 i. ♃. bg. &c. Cin. *Colby.*

1446. Luzula campestris, *De Cand.* Field wood-rush. April–May. 6 to 12 i. ♃. md. Ky. *St.*

### CCXLV. *MELANTHACEÆ.*—The colchicum tribe.

Acrid and poisonous in every species. Lind. 270.

1447. Melanthium hybridum. Bunch flower. June–July. *w.* 2 f. ♃. Mo. Ter. *Jas.*
1448. M. virginicum, *Linn.* Tall melanthium. July. *g–w.* 3 to 4 f. ♃. wp. md. mh. Pittsburgh. *Peter.* Louisville. *M'Murt.* Prairies of Ohio.
1449. M. glaucum, *Nutt.* July–Aug. *w.* 1 f. ♃. Borders of Lake Erie, &c. in calcareous soil. *Nutt.*
1450. Tofielda glutinosa, *Michx.* ♃. About Detroit. *Nutt.*
1451. Helonias dioica, *Pursh.* Blazing star. June. *w.* 1 to 2 f. ♃. Damp grounds. W. *Bk.* Dry open woods. Mar. O. Ky. Acrid, medicinal. An infusion of the root is anthelmintic; the tincture is tonic. Lind. 270.
1452. Veratrum viride, *Ait.* Poke root. American hellebore. June–July. *g.* 3 to 5 f. ♃. Swamps. md. Louisville. *M'Murt.* Acrid, emetic, powerfully stimulant, followed by sedative effects. Big. ii. 125.
1453. V. angustifolium, *Pursh.* June. *g–w.* 2 to 3 f. ♃. Grassy prairies of Ohio and Tenn. *Nutt.* Ky. *St.*
1454. Zigadenus elegans, *Pursh.* Zigadene. June. *w.* ♃. Ark. *Jas.*

### CCXLVI. *PONTEDEREÆ.*—The pickerel weed tribe.

1455. Heteranthera reniformis, *Michx.* Mud plantain. July–Aug. *w.* Sometimes partly floating. 4 to 8 i. ♃. rb. Mud. Ky. *St.* Lockbourne, O.
1456. H. ovalis, *Michx.* July. *b.* ♃. Ky. *St.*
1457. Schollera graminifolia, *Vahl.* Yellow-eyed water grass. July–Aug. *y.* Submersed. 6 to 30 i. ♃. In flowing streams. O. Ky. Fig. Bart. Fl. N. A. ii. 53.
1458. Pontederia cordata, *Linn.* Pickerel weed. Aug. *b.* 1 to 2 f. ♃. Ponds. Ky. *St.*
1459. P. angustifolia, *Pursh.* July. *b.* Ponds. Ky. *St.*

### CCXLVII. *ASPHODELEÆ.*—The asphodel tribe.

1460. Allium triflorum, *Raf.* Leek. Mountain leeks. May–June. *w.* 6 to 8 i. ♃. Woods. Wor. O. Used as a potherb. The different species of Allium (including the garlic and cultivated onions) are more or less stimulant, diuretic, and expectorant. Lind. Nat. Syst. 272.
1461. A canadense, *Linn.* Meadow garlic. May–June. Pale *r.* 18 i. ♃. md. O. Ky.
1462. A. cernuum, *Roth.* Nodding wild onion. July. Pale *r.* 1 to 2 f. ♃. dp. Hill sides. O. Ky.
1463. A. tricoccum, *Ait.* Three-seed leek. June–July. *w.* 1 f. ♃. Upland woods. Ky. *St.* O.

1464. A. vineale, *Linn.* Field garlic. June–July. Pale *r.* 2 f. ♃. md. Intr. Louisville. *M'Murt.*

1465. A. striatum, *Pursh.* Scentless onion. Ap. *w.* 8 to 12 i. ♃. Pine barrens. *Ell.* Timbered alluvions near St. L. *Bk.*

1466. A. angulosum, *Linn.* *w* or *p.* ♃. Damp. Mo. Ter. *Nutt.*

1467. Aletris farinosa, *Linn.* Star-grass. July. *w.* 2 f. ♃. Sandy woods. Prairies of Mich. *Hough.* Louisville. *M'Murt.* Tonic, stomachic, emetic in large doses. Big. Med. Bot. iii. 92.

1468. Phalangium esculentum, *Nutt.* Quamash. May. *b.* 12 to 18 i. ♃. bt. Hill-sides, &c. W. The root is an article of diet among the Indians of the Rocky Mountains. *Lewis.*

1469. Brodiæa grandiflora, *Smith.* Missouri hyacinth. April. ♃. Plains of the Missouri. *Lewis.*

### CCXLIX. *SMILACEÆ.*—The smilax tribe.

*Smilax.*—Green brier.

The roots of several species (S. sassaparilla, china, pseudo-china et aspera) are much used in medicine, under the name of sassaparilla, for their alterative qualities. It is quite probable that some of our western species may be advantageously substituted for the worm-eaten, time-injured sassaparilla of the shops.

1470. Smilax rotundifolia, *Linn.* Green brier. June. *w–g.* Climbing. ♄. Berries black. Thickets, &c. O. Ky.

1471. S. pseudo-china, *Linn.* May–June. Climbing. ♄. Woods. Wor. O. Medicinal. Ell. Bot. iii. 700.

1472. S. pandurata, *Pursh.* July. Twining. ♄. Berries black. Woods. Ohio.

1473. S. walteri, *Pursh.* July. ♄. Berries red. Woods. Mar. O.?

1474. S. cincidifolia, *Pursh.* ♄. Wor. Mar. O.

1475. S. hastata, *Willd.* July. Twining. ♄. Rich shaded soils. Louisville. *M'Murt.*

1476. S. bona-nox, *Linn.* Vine. ♄. Margins of swamps. Ky. *St.*

1477. S. caduca, *Linn.* June. ♄. Dry fields. Louisville. *M'Murt.*

1478. S. peduncularis, *Muhl.* June. *w–g.* Climbing 3 to 5 f. ♃. Low woods. O. Ky.

1479. S. herbacea, *Linn.* June–July. *g.* 2 to 3 f. ♃. Woods. Ky. *St.* Cin. O. Fig. Bart. Fl. N. A. ii. 99

*Convallaria.*—Solomon's seal.

1480. Convallaria bifolia, *Linn.* May. *w.* 4 to 6 i. ♃. rs. sw. Wor. O.

1481. C. trifolia, *Linn.* June–July. *w.* 6 i. ♃. Alpine swamps. Mich. *Eat.*

1482. C. stellata, *Linn.* May–June. *w.* 1 f. ♃. River banks. Ohio. *Paddock.*

1483. C. racemosa, *Linn.* June–July. *g–w.* 18 to 24 i. ♃. Woods. W. Medicinal. See 1484.

1484. C. multiflora, *Linn.* Solomon's seal. June–July. *g–w.* 2 to 3 f. ♃. ow. bt. W. Astringent, corroborant, *Beach.* Raf. Med. Fl.

1485. C. pubescens, *Pursh.* May–June. *y–w.* 18 i. ♃. rs. Ohio. *Paddock.*

1486. C. majalis, *Willd.* Lily of the valley. June. *w.* ♃. Louisville. *M'Murt.*

1487. Streptopus lanuginosus, *Michx.* May. *g–y.* 18 i. ♃. Woods. Ky. *St.*
1488. Medeola virginica, *Linn.* Cucumber root. May–June. *y.* 12 to 18 i. ♃. Woods. W. Diuretic, hydragogue. Bart. ii. 147. Raf. Med. Fl.
1489. Uvularia perfoliata, *Linn.* Bellwort. May–June. Pale *y.* 8 to 12 i. ♃. Shady hills. W. The root is an empirical remedy for snake bites. Fig. Bart. Fl. N. A. i. 112.
1490. U. grandiflora, *Smith.* Large bellwort. June. *y.* 10 to 20 i. ♃. rs. W. Reputed virtues like the last.
1491. U. sessilifolia, *Linn.* May. Pale *y.* 8 to 10 i. ♃. sw. W. Fig. Bart. Fl. ii. 55.

*Trillium.*—Birthroot. Wake-robin. Trillium.

According to De Candolle, the roots of these plants are violently emetic. Lind. 276. Dr. Beach regards them as astringent, pectoral, tonic, antiseptic and alterative. Beach's Am. Pract. In domestic practice they are administered preparatory to parturition, and hence the name *birthroot.*

1492. Trillium sessile, *Linn.* April–May. Dark *p.* 8 i. ♃. Fertile woods. W.

1493. T. recurvatum, *Bk.* Bot. 300. May. *p.* 8 to 10 i. ♃. sw. On the Miss. &c. *Bk.*

1494. T. viride, *Bk.* Ap. Dark *g.* 8 to 12 i. ♃. sw. W. *Bk.*

1495. T. pendulum, *Willd.* May. *w* with *r* veins. 1 f. ♃. Mts. Ky. *St.*

1496. T. erectum, *Linn.* May. Dark *p.* 12 to 15 i. ♃. sw. W. Ky. *St.* Ill. riv. *Bk.* O.

*b.* var. album, *Pursh.* May. *w.* 12 to 15 i. ♃. sw. O.

1497. T. grandiflorum, *Salisb.* May. *w.* 8 to 12 i. ♃. md. ow. W.

1498. T. cernuum, *Linn.* May. *w.* 12 to 18 i. ♃. sw. Ky. *St.* Fig. Bart. Fl. ii. 15.

1499. T. ovatum, *Pursh.* Ap. *p.* ♃. Northern Andes. *Nutt.*

1500. T. nivale, *mihi.* *Root* perennial, tuberous præmorse, of a sweet and mawkish taste. *Stem* glabrous, 2 to 3 inches high. *Leaves* short petioled, oval, obtuse, 12 to 16 lines long, 8 to 10 lines wide, glabrous, 5-nerved. *Peduncle* erect, near 4 lines in length. *Sepals* lance-ovate, obtuse, 5-nerved. *Petals* spatulate-obovate, obtuse, nerved, white, 6 to 8 lines in length, one third longer than the calyx. Flowers in March. I have met with this interesting plant only on the east bank of the Scioto river, near Dublin, inhabiting a steep declivity, among comminuted fragments of limestone. Its delicate white blossom is one of the earliest of that region.

CCL. *DIOSCOREÆ.*—The yam tribe.

1501. Dioscorea villosa, *Linn.* Yam root. China root. May–June. *y–w.* Climbing vine, 6 to 12 f. ♃. Root woody, tortuous, echinate. ow. bt. W. An infusion of the root is unquestionably a valuable remedy in bilious colic. An ounce of the powdered root is to be boiled in a pint of water, and half of it given at once. It acts with remarkable promptitude. I have been informed that Dr. Miller of Neville, Ohio, values the tincture highly as an expectorant. He says it is also diaphoretic, and in large doses emetic.

CCLI. *LILIACEÆ.*—The lily tribe.

1502. Lilium canadense, *Linn.* Turk's-cap. Nodding lily. July–Aug. *y* and *orange*, with dark spots. 2 to 3 f. ♃. md. wp. W.

1503. L. superbum, *Linn.* Superb lily. July. *Orange* with dark spots. 4 to 6 f. ♃. Wet meadows. Ky. *St.*

1504. L. philadelphicum, *Linn.* July–Aug. Dark *orange*, spotted at the base. 18 i. ♃. Darby Plains, O.

1505. L. catesbæi, *Walt.* Catesby's lily. June–Aug. *r.* Spotted with *y* and *brown.* 18 i. ♃. Sandy meadows. Ky. *St.* Prairies near St. L. *Bk.*

1506. Yucca angustifolia, *Pursh.* Adam's needle. July. *w.* ♃. Banks of the Missouri. Plt. *Nutt.*

1507. Erythronium americanum, *Smith.* Adder-tongue. Dogtooth violet. Ap.–May. *y*. 6 to 8 i. ♃. bt. ow. W. Emetic. Big. Am. Med. Bot. iii. 51.

1508. E. albidum, *Nutt.* White erythronium. April–May. *w.* 6 i. ♃. bt. W.

CCLIII. *RESTIACEÆ.*—The pipewort tribe.

1509. Eriocaulon pellucidum, *Michx.* Pipewort. Aug. *w.* 4 to 8 i. ♃. In ponds. New York. Lake Sup. *Houghton.* Perhaps in the interjacent regions.

CCLV. *TYPHACEÆ.*—The reed-mace tribe.

1510. Typha latifolia, *Linn.* Reed-mace. Cat's-tail. Cat-tail flag. July–Aug. 5 to 6 f. ♃. mh. pl. Ohio. Ky. The leaves are used by coopers, for rendering barrels tight.

1511. T. angustifolia, *Linn.* Lesser reed-mace. July–Aug. 4 to 5 f. ♃. mh. pl. Central O.

1512. Sparganium americanum, *Nutt.* Burr reed. Aug. 1 f. ♃. Ponds and lakes. O. Ky.

1513. S. ramosum, *Smith.* Branched burr reed. July–Aug. 2 f. ♃. pl. Stagnant waters. Louisville, *M'Murt.*

1514. S. angustifolium, *Michx.* Floating burr reed. Aug. ♃. Lakes, &c. Ponds on Darby Plains, O.?

CCLVI. *AROIDEÆ.*—The arum tribe.

1515. Arum triphyllum, *Linn.* Indian turnip. May–June. *g, y & p.* 1 to 2 f. ♃. sw. W. Acrid irritant, secernant stimulant, pectoral. Big. Med. Bot. i. 52.

1516. A. dracontium, *Linn.* Green dragon. June–Jul. 1 f. ♃. sw. bt. W.

1517. Lecontia virginica, *Cooper.* Poison arum. July. 12 to 18 i. ♃. wp. Walnut plains, Picaway Co. O. Louisville. *M'Murt.*

1518. Symplocarpus fœtidus, *Nutt.* Skunk cabbage. March. *p. g & y.* 1 to 2 f. ♃ Wet woods and meadows. W. Stimulant, antispasmodic, narcotic. Bart. Med. Bot. i. 123. Big. Med. Bot. ii. 41.

1519. Orontium aquaticum, *Linn.* Golden club. May. *y.* 12 to 20 i. ♃. In water. Louisville. *M'Murt.* Fig. Bart. Fl. N. A. ii. 1.

1520. Acorus calamus, *Linn.* Sweet flag. June. *g–w.* 2 to 3 f. ♃. Swamps. W. Stimulant, tonic, stomachic. Bart. Med. Bot. ii. 63.

CCLVIII. *FLUVIALES.*—The pond-weed tribe.

1521. Caulinia fragilis, *Willd.* Water knot-grass. Aug. 1 to 3 f. submersed. ⊙. pl. Miami country.

*Potamogeton.* Pond weed.

* *Upper leaves floating.*

1522. Potamogeton natans, *Linn.* Pond-weed. July–Aug. 1 to 4 f. ♃. Ponds and lakes. O. Ky.
1523. P. fluitans, *Linn.* River-weed. July–Aug. 1 to 4. ♃. Running streams. Ohio.
1524. P. heterophyllum, Various-leaved pond-weed. Aug. 2 to 3 f. ♃. pl. A pond 6 miles east from Cin. O.

* * *Leaves all submersed.*

1525. P. perfoliatum, *Linn.* Perfoliate pond-weed. July. ♃. pl. Louisville. *M'Murt.*
1526. P. lucens, *Linn.* Shining pond-weed. Aug. Long and large. ♃. pl. Streams, &c. Ky. St.
1527. P. compressum, *Linn.* Flat-stem pond-weed. July–Aug. Small, branched; leaves 2 i. ♃. In water. Ky. St. Old River, Hamilton, O.
1528. P. pectinatum, *Linn.* Fennel-leaved pond-weed. June. 1 to 2 f. ♃. pl. Rivers. Ohio.
1529. P. pauciflorum, *Pursh.* P. gramineum, *Michx.* Grass-leaved pond-weed. July–Aug. 8 to 18 i. ♃. pl. &c. O. Ky.
1530. P. acutifolium, *Link.* Sharp-leaved pond-weed. July. ♃. pl. Ky. *St.* Leaves linear, acuminate, 3-nerved; spike oval, compact, equal in length to the peduncle. Hook. Brit. Fl. 74.

### CCLIX. *JUNCAGINEÆ.*—The arrow-grass tribe.

1531. Triglochin maritimum, *Linn.* Arrow-grass. July. *g.* 18 i. ♃. Salt marshes usually. West to Mich. *Bk.*

### CCLX. *PISTIACEÆ.*—The duckweed tribe.

1532. Lemna trisulca, *Linn.* Duck's meat. Ivy-leaved duckweed. July. Floating and submersed, detached. 6 lines. ⊙. pl. Ky. *St.* Miami country.
1533. L. polyrhiza, *Linn.* Duckweed. June–July. Floating, detached. 3 to 4 lines. ⊙. pl. Mich. *Eat.* O.
1534. L. minor, *Linn.* Lesser duckweed. Water flax-seed. June–July. Floating, detached. 1 to 2 lines. ⊙. pl. O. Ky.

TRIBE II. GLUMACEÆ. *Flowers enveloped in glumaceous bracts, destitute of the true calyx and corolla.*

### CCLXI. *GRAMINEÆ.*—THE GRASS TRIBE.

### DIV. I. AGROSTIDEÆ.

*Agrostis.* Bent grass.

1535. Agrostis vulgaris, *Smith.* Red-top. July. 18 to 20 i. ♃. md. W. Intr.
1536. A alba, *Linn.* Bonnet grass. Aug. 18 i. ⊙. md. St. L. *Bk.* Cin. O. *Colby.*
 *b.* var. decumbens, *Eat.*Fiorin grass. Dry pastures and woods, Germantown, O. *Frank.*
1537. A. lateriflora, *Michx.* Aug.–Sept. 2 f. ♃. Swamps, wp. W. Ky. *St.* Cin. *Colby.* Germantown. *Frank.* Dayton, O.
1538. A. tenuiflora, *Willd.* July–Aug. 3 f. ♃. Rocky woods. Ky. *St.*
1539. A. clandestina, *Spreng.* Sept. 2 f. ♃. Dry hills. Ky.? *St.*
1540. A. dispar, *Michx.* Ky.? *St.*
1541. A. brevifolia, *Nutt.* 1 f. Arid plains, Mo. Ter. *Nutt.*
1542. A. cæspitosa, *Torr.* 4 i. Plt. *Jas.*
1543. Polypogon racemosum, *Nutt.* Beard grass. Aug. 8 f. ♃. Alluvions. Mo. & Miss. St. L. *Nutt.* 3 to 4 f. bg. W. *Bk.*
1544. Cinna arundinacea, *Willd.* Indian reed. Aug. 2 to 5 f. ♃. Wet grounds, Ky. *St.*
1545. Phleum pratense, *Linn.* Herd's grass. Timothy grass. June–Aug. 2 to 3 f. ♃. md. Intr. W.
1546. Muhlenbergia diffusa, *Schreb.* Drop-seed grass. July. 18 i. ♃. Fields. O. Ky. *Nutt. St.*
1547. M. erecta, *Schreb.* July. 2 to 3 f. ♃. rs. Woods. Germantown, O. *Frank.* Ky. *St.*
1548. Phalaris americana, *Ell.* American canary grass. July –Ang. 2 to 5 f. ♃. Swamps, Louisville. *M'Murt.*
1549. Trichodium laxiflorum, *Michx.* Thin grass. May–June. 18 i. ♃. Dry fields. O.
1550. Alopecurus geniculatus, *Linn.* Wild fox-tail grass. June. 12 to 18 i. ♃. Wet meadows. St. L. *Bk.* N. W. Ter. *Houghton.*
1551. A. pratensis, *Linn.* Fox-tail grass. May–Aug. 2 to 4 f. ♃. Fields and pastures. Intr. O. *Frank.*
1552. Crypsis squarrosa, *Nutt.* ⊙. 3 to 4 i. Arid plains, Mo. Ter. *Nutt.*

### Div. II. Paniceæ.

*Panicum.* Panic grass.

1553. Panicum crus-galli, *Linn.* Cocksfoot grass. Aug.–Sept. 2 to 4 f. ⊙. cf. Intr. W.
1554. P. clandestinum, *Linn.* July–Aug. 2 to 3 f. ♃. Moist woods. Ky. *St.* Cin. *Colby.*
1555. P. latifolium, *Linn.* May–July. 1 f. ♃. ow. Thickets. W.
1556. P. dichotomum, *Linn.* Aug. 8 to 16 i. ♃. Dry woods, &c. Ky. *St.* Mar. O.
1557. P. nitidum, *Lam.* June–July. 18 i. to 2 f. ♃. Barrens, &c. Ky. *St.*
1558. P. agrostoides, *Muhl.* Aug. 2 to 3 f. ♃. Wet meadows. Ky. *St.*
1559. P. proliferum, *Lam.* Sept. 2 to 4 f. f. ♃. Wet meadows. Ky. *St.*
1560. P. virgatum, *Linn.* July–Aug. 3 to 4 f. ♃. Ky. *St.*
1561. P. capillare, *Linn.* Aug. 1 to 2 f. ♃. cf. W.
*b.* var. sylvaticum, *Torr.* Dry woods. O.
1562. P. laxiflorum, *Lam.* ⊙. Fields. Germantown, Ohio. *Frank.*
1563. Setaria glauca, *Beauv.* Bottle grass. July–Aug. 18 i. ⊙. Intr. rd. &c. O.
1564. S. verticillata, *Beauv.* Bristle grass. July. 18 i. ⊙. Sandy grounds. Intr. Cin. O. *Colby.*
1565. Piptatherum nigrum, *Torr.* Black-seeded millet grass. Aug. 2 to 3 f. ♃. Rocky hills, Ky. *St.*
1566. Digitaria sanguinalis, *Scop.* Crab grass. Aug.–Sept. 1 to 2 f. ⊙. cf. &c., O.
1567. D. glabra, *Roem.* Smooth finger grass. Aug.–Sept. 1 f. ⊙. Sandy fields, Cin. O. *Colby.*
1568. D. filiformis, *Ell.* Thread-stem finger grass. Aug. 1 to 2 f. ⊙. Sandy fields, Ky. *St.*
1569. D. serotina, *Michx.* June–Aug. 12 to 18 i. ⊙. Wet places. *Muhl.* Canal banks, O.?
1570. Ceresia fluitans, *Pers.* Floating ceresia. Oct. 1 to 3 f. ⊙.? In river swamps. Ky. *St.*
1571. Beckmannia crucæformis, *Juss.* July. Plt. *Jas.*
1572. Cenchrus tribuloides, *Linn.* Hedge-hog grass. July–Aug. 18 i. ⊙. Sandy soils. Louisville. *M'Murt.*
1573. Paspalum ciliatifolium, *Michx.* Sept. 18 i. ♃. Sandy fields. Ky. *St.*
1574. P. plicatulum, *Michx.* Ky. *St.*
1575. P. læve, *Michx.* Aug. 1 to 2 f. ♃. md. Louisville. *M'Murt.*

1576. Tripsacum dactyloides, *Linn.* Sesame grass. Aug. 5 to 7 f. ♃. md. Louisville. *M'Murt.* W. *Bk.*

DIV. III. AVENACEÆ.

1577. Stipa juncea, *Pursh.* Feather grass. Aug. ♃. Mo. Ter. *Nutt.* N. W. Ter. *Houghton.*
1578. S. parviflora, *Desf.* *Nutt.* 1 to 2 f. ♃. Plains of Missouri. *Nutt.*
1579. S. sericea, *Michx.* Sept. 2 f. ♃. Mo. Ter. Plt. *Jas.*
1580. S. membranacea, *Pursh.* June–July. 2 to 3 f. ♃. Missouri banks. *Bradbury.* Grassy plains, Mo. Ter. *Nutt.* Stony hills, O. *Frank.*
1581. Aristida pallens, *Linn.* Mo. Ter. *Nutt.* *Jas.*
1582. Calamagrostis mexicana, *Nutt.* Aug. 3 to 4 f. ♃. bg. West to Mich. *Bk.*
1583. C. confinis, *Spreng.* Germantown, O. *Frank.*
1584. Anthoxanthum odoratum, *Linn.* Sweet-scented vernal grass. June–Aug. 1 f. ♃. Moist meadows. Intr. Cin. O. *Frank.*
1585. Aira flexuosa, *Linn.* Hair grass. June. 1 to 2 f. ♃. sw. W. *Bk.* Mich. *Houghton.*
1586. A. capillacea, *Lam.* 8 i. Slate hills and sandy pastures. *Pursh.* sw. Germantown, O. *Frank.*
1587. A. mollis, *Muhl.* Soft hair grass. Ap.–May. 1 to 2 f. Banks of the Miami canal, &c. *Frank.* Ky. *St.*
1588. Holcus lanatus, *Linn.* Soft grass. July. 18 i. ♃. Wet meadows. N. W. Ter. *Houghton.* W. *Bk.*
1589. Danthonia spicata, *Beauv.* Wild oats. June–Aug. 1 to 2 f. ♃. Woods and fields. Ky. *St.* Cin. *Colby.* W. to Mich. *Bk.*
1590. Arrhenatherum kentuckiensis, *Torr.* ♃. Ky. *St.*

DIV. IV. FESTUCACEÆ.

*Poa.* Meadow grass.

1591. Poa annua, *Linn.* Ap.–Aug. 6 to 8 i. ⊙. Fields. Ky. *St.* O. *Frank.* St. L. *Bk.*
1592. P. pungens, *Torr.* Ap. 18 i. ♃. Rocky woods. Ky. *St.* O. *Frank.*
1593. P. pratensis, *Linn.* May–July. 2 to 3 f. ♃. Intr. md. Ky. *St.* Cin. O. *Colby.*
1594. P. parviflora, *Pursh.* July. 12 to 18 i. ♃. sw. Ky *St.*
1595. P. trivialis, *Linn.* July. 2 to 3 f. ♃. Wet md. Cin. *Colby.* Ky. *St.*
1596. P. compressa, *Linn.* Blue grass. June–July. 12 to 18 i. ♃. Fields and woods. Ky. *St.* Cin. *Colby.* St. L. *Bk.*

1597. P. serotina, *Ehrh.* One of the grasses called *red-top.* June. 2 to 3 f. ♃. Wet md. *Bk.* Woods and fields, Germantown, O. *Frank.*
1598. P. nervata, *Willd.* June. 3 to 4 f. ♃. Wet. O. Ky.
1599. P. obtusa, *Muhl.* Aug. 3 to 4 f. ♃. Swamps, &c. Germantown, O. *Frank.*
1600. P. canadensis, *Torr.* July. 3 to 4 f. ♃. Swamps. Cin. *Colby.*
1601. P. capillaris, *Linn.* July–Aug. 1 f. ☉. Sandy fields. Ky.? *St.*
1602. P. pectinacea, *Michx.* July. 8 to 12 i. ☉. Sandy fields. Ky. *St.*
1603. P. reptans, *Michx.* July–Aug. 6 to 8 i. ☉. Sandy banks. rb. W.
*b.* var. cæspitosa, *Torr.* St. L. *Bk.*
1604. P. eragrostis, *Linn.* July–Aug. 12 to 18 i. ☉. Sandy fields, pastures, &c. O. Ky.
1605. P. airoides, *Nutt.* 4 to 5 f. Depressed situations. Mo. Ter. *Nutt.*
1606. P. multicaulis, *Raf.* Louisville. *M'Murt.* Ky. *St.*

*Festuca.* Fescue grass.

1607. Festuca tenella, *Willd.* June. 8 to 12 i. ☉. Sandy fields. Ky. *St.*
1608. F. elatior, *Linn.* June. 3 to 4 f. Wet md. Ky. *St.*
1609. F. pratensis, *Huds.* June–July. 1 to 2 f. ♃. md. Ky. *St.*
1610. F. nutans, *Willd.* June. 3 f. ♃. Woods and hills. W. *Bk.*
1611. F. spicata, *Pursh.* June. 1 f. Banks of the Missouri. *Nutt.*
1612. F. clandestina, *Pursh.* 8 i. Cin. O. *Colby.*
1613. Dactylis glomerata, *Linn.* Orchard grass. June. 2 to 3 f. ♃. md, &c. Ky. *St.* Cin. O.
1614. Kœleria nitida, *Nutt.* 8 i. ☉? Plains of the Missouri. *Nutt. Jas.*
1615. K. truncata, *Torr.* June. 2 f. ♃. Dry woods. Ky. *St.*

*Bromus.* Brome grass.

1616. Bromus secalinus, *Linn.* Chess. June. 2 to 3 f. ☉. cf. Intr. O. Ky.

1617. B. purgans, *Linn.* Aug. 2 to 4 f. ♃. Wet md. Germantown, and Cin. O. *Frank.* Dayton, O.

1618. B. ciliatus, *Linn.* June. 3 f. ♃. River banks. Cin. O. *Colby.* Ky. *St.*

1619. B. pubescens, *Muhl.* June. 4 f. ♃. Woods. Ky. *St.*

1620. B. mollis, *Linn.* June. 2 f. ♂. Fields. Ky.? *St.*
1621. B. altissimus, *Pursh.* June. 7 f. ♃. Banks of the Missouri. *Pursh.* Mo. Ter. *Nutt.* Cin. O. *Colby.*
1622. B. arvensis, *Linn.* June. 2 to 3 f. ⊙. cf. Hook. Bri. Fl. p. 49. Meadows, Germantown, O. *Frank.* Intr.?

1623. Uniola latifolia, *Michx.* Aug. 2 to 3 f. ♃. Ky. *St.* Cin. O.
1624. U. gracilis, *Michx.* Aug. 3 to 4 f. ♃. Sandy swamps. Ky. *St.*
1625. U. spicata, *Linn.* Aug.–Sept. 18 i. ♃. Salt marshes, Mo. Ter. *Jas.*
1626. Glyceria fluitans, *Brown.* June–July. 3 to 5 f. ♃. Wet grounds. Ky. *St.* Cin. *Colby.* N. W. Ter. *Houghton.*
1627. Melica speciosa, *Muhl.* Melic grass. June. 3 to 4 f. ♃. Ky. *St.* Cin. *Colby.*
1628. M. diffusa, *Pursh.* June. ♃. Ky. *St.*
1629. Tricuspis seslerioides, *Torr.* Larger red-top. Aug. 4 to 5 f. ♃. Sandy fields. dp. Louisville. *M'Murt.* Ky. *St.* Middletown, O.
1630. Sesleria dactyloides, *Nutt.* Moor grass. May–June. 4 to 5 i. ♃. Grassy plains of the Missouri. *Nutt.* Plt. *Jas.*
1631. Diarrhena americana, *Torr.* *Beauv.* 3 to 4 f. ♃. Banks of the Ohio. *Raf.* Miami country. Festuca diandra, *Michx.*

Div. V. Chlorideæ.

1632. Chloris secundus, *Pursh.* ♃. Upper Louisiana. *Bradbury.*
1633. Eleusine indica, *Lam.* Wire grass. July.–Nov. 1 to 2 f. ⊙. cf. Cin. *Colby.* Germantown, O. *Frank.*
1634. Oxydenia attenuata, *Nutt.* Aug. 2 to 3 f. ⊙. River banks. Ky. *St.*
1635. Atheropogon apludoides, *Muhl.* Aug. 18 i. ♃. Rocky hills. W. *Nutt.*
1636. A. oligostachyum, *Nutt.* 8 to 12 i. ♃. Plains of the Missouri. *Nutt.*

Div. VI. Hordeaceæ.

1637. Hordeum jubatum, *Linn.* Squirrel-tail grass. June. 2 f. ♂. mh. Mo. Ter. Plt. *Jas.* Calcareous islands of Lake Michigan. *Nutt.*
1638. H. pusillum, *Nutt.* Dwarf barley grass. 4 to 6 i. Saline plains of the Missouri. *Nutt.* Ky. *St.*
1639. Triticum repens, *Linn.* Couch grass. Quack. July. 2 f. ♃. Fields, W. *Bk.*

1640. Elymus virginicus, *Linn.* Lime grass. Wild rye. July–Aug. 3 to 4 f. ♃. River banks, &c. W.
1641. E. canadensis, *Linn.* Canadian lime grass. Aug. 3 to 4 f. ♃. River banks, &c. Mar. O.
*b.* var. glaucifolius, *Torr.* July. St. L. *Bk.*
1642. E. villosus, *Muhl.* July. 2 to 3 f. ♃. Hills. Germantown. O. *Frank.*
1643. E. hystrix, *Linn.* July. 3 f. ♃. rs. Cin. *Colby.*
1644. E. striatus, *Willd.* June. 8 i. ♃. sw. *Bk.* July. 3 to 4 f. ♃. *Eat.* Cin. *Colby.* S. Croix river, N. W. Ter. *Hough.*
1645. Spartina cynosuroides, *Willd.* Marsh grass. Aug. 4 to 10 f. ♃. wp. md. Ky. *St.* Plt. *Jas.* Wet prairie, Hamilton, O.
1646. S. glabra, *Muhl.* Smooth cord grass. Aug.–Sept. 3 to 5 f. ♃. mh. W. *Bk.*
1647. Lepturus paniculatus, *Nutt.* June. 10 i. ⊙. Saline plains, Mo. Ter. *Nutt.* Plt. *Jas.*
1648. Ægilops hystrix, *Nutt.* 4 to 6 i. ♃. Arid plains of the Missouri. *Nutt.* Plt. *Jas.*

### Div. VII. Saccharineæ.

*Andropogon.* Beard grass.

1649. Andropogon scoparius, *Michx.* Aug. 3 to 4 f. ♃. dp. O. Ky.
1650. A. virginicus, *Linn.* Sept. 3 f. ♃. Swamps. Ky. *St.*
1651. A. nutans, *Linn.* Aug.–Oct. 4 to 8 f. ♃. dp. wp. O.
1652. A. furcatus, *Muhl.* Aug.–Sept. 2 to 3 f. ♃. Rocky banks of streams. Ky. *St.* N. W. Ter. *Hough.*
1653. A. ciliatus, *Ell.* Sept. 3 f. ♃. Pine barrens. *Ell.* Ky. *St.*

### Div. VIII. Oryzeæ.

1654. Zizania aquatica, *Lamb.* Wild rice. Water oats. Aug. 4 to 6 f. ♃. In Water. Abundant in the Great Lakes. *Schoolcraft.* Dayton, O. *Dr. Locke.* The seed is an article of diet among the Western Indians.
1655. Z. miliacea, *Michx.* Aug. 6 to 10 f. ♃. In water. Near Dayton, O., on Hoffman's prairie.
1656. Oryzopsis asperifolia, *Michx.* Mountain rice. April–May. 18 i. ♃. Ditches, &c. Miami country. *Frank.*
1657. Leersia virginica, *Willd.* Rice grass. Aug. 2 to 4 f. ♃. Wet woods. O.
1658. L. oryzoides, *Swartz.* White grass. Aug.–Sept. 3 to 5 f. ♃. mh. wp. &c. Ky. *St.* St. L. *Bk.* Miami country.
1659. L. lenticularis, *Michx.* Catch-fly grass. July. 2 to 4 f. ♃. Wet places, Germantown and Cin. *Frank.*

1660. Zea mays, *Linn.* Indian corn.
*b.* var. præcox, *Nutt.* Early Mandan corn. Cultivated among the Missouri Indians. *Nutt.*

DIV. IX. BAMBUSINÆ.

1661. Arundinaria macrosperma, *Michx.* Cane. April. 3 to 15f. ♄. Louisville. *M'Murt.* Ky. *St.* Ark. *Jas.* var. gigantea, 30 to 40 f.

CCLXII. *CYPERACEÆ.*—THE SEDGE TRIBE.

§ 1. *Cyperaceæ veræ.*

1662. Cyperus inflexus, *Muhl.* Aug.–Sept. 2 to 3 i. ♂. Banks of streams, margins of ponds. Ky. *St.* St. L. *Bk.*
1663. C. flavescens, *Linn.* Aug.–Sept. 8 to 12 i. ♃. Wet soils. Ky. *St.* O.
1664. C. nuttallii, *Torr.* Aug.–Sept. 5 to 12 i. ♃. Salt marshes. *Bk.* Ky. *St.*
1665. C. dentatus, *Torr.* Sept. 10 to 12 i. ♃. mh. Louisville, *M'Murt.*
1666. C. strigosus, *Linn.* Cyperus. Aug.–Sept. 2 to 3 f. ♃. Low wet grounds. mh. W.
1667. C. phymatodes, *Muhl.* Aug. 12 to 18 i. ♃. Moist grounds. Ky. *St.* Central O.
1668. C. mariscoides, *Ell.* Aug. 8 to 10 i. ♃. Rocky grounds. Cin. *Colby.* W. *Bk.*
1669. C. flavicomis, *Michx.* July. ♃. Boggy woods. *Bk.* Wet sandy places, Miami country. *Frank.*
1670. C. poæformis, *Pursh.* July. 8 i. Banks of rivulets, Germantown, O. *Frank.*
1671. C. alterniflorus, *Schw.* 1 f. ♃. River St. Clair. Mich. *Houghton.*
1672. Dulichium spathaceum, *Pers.* Galingale. Aug. 18 i. ♃. mh. &c. O.
1673. Kyllingia pumila, *Michx.* July. 3 to 6 i. ♃. Damp gravelly situations. Ky. *St.* Miami country.

§ 2. *Scirpeæ.*

*Scirpus.* Club-rush.

1674. Scirpus palustris, *Linn.* June. 1 to 2 f. ♃. mh. &c. Germantown, O. *Frank.* Louisville. *M'Murt.*
1675. S. capitatus, *Linn.* July. 8 to 14 i. ♃. bg. md. Ky. *St.* Cin. *Colby.* Central O.
1676. S. acicularis, *Linn.* June–July. 3 to 6 i. ♃. Muddy margins of ponds. W.
1677. S. intermedius, *Muhl.* Sept. 3 to 4 i. ♃. mh. Cin. O. *Colby.*

1678. S. tenuis, *Willd.* June–July. 8 to 12 i. ♃. mh. Swamps. Ky. *St.* O.
1679. S. lacustris, *Linn.* Bull-rush. June. 4 to 8 f. ♃. pl. mh. O. Ky.
1680. S. brunneus, *Muhl.* Aug.–Sept. 2 to 3 f. ♃. Margins of ponds. O. Ky.
1681. S. atrovirens, *Muhl.* June. 2 f. ♃. Wet md. Ky. *St.*
1682. S. capillaris, *Linn.* Aug. 8 i. ☉.? Wet grounds.—Lovisville. *M'Murt.* O.
1683. S. autumnalis, *Linn.* July–Oct. 8 to 12 i. ♃. Low woods. Ky. *St.*
1684. S. simplex, *Ell.* June. 8 to 13 i. ♃. bg. &c. Banks of creeks. Germ. O. *Frank.*
1685. S. americanus, *Pers.* Aug. 3 to 5 f. *Eat.* 2 to 3 f. *Ell.* ♃. Damp soils. Ky. *St.*
1686. S. maritimus, *Linn.* July–Aug. 3 to 4 f. ♃. Salt marshes, usually. Mo. Ter. *Jas.*
1687. Tricophorum cyperinum, *Pers.* Red cotton grass. Aug. 3 to 5 f. ♃. Wet grounds. Ky. *St.*
1688. T. lineatum, *Pers.* Aug. 2 to 3 f. ♃. Swamps. Borders of ponds, Missouri. *Bk.* Cin. *Colby.* Mo. Ter. *Jas.*
1689. Eriophorum polystachyon, *Linn.* Cotton grass. July. 1 to 2 f. ♃. Swamps. Louisville. *M'Murt.*
1690. Mariscus ovularis, *Vahl.* July–Aug. 6 to 18 i. ♃. bg. and low grounds. Louisville. *M'Murt.*
1691. Fuinera squarrosa, *Michx.* Umbrella grass. Aug.–Sept. 1 to 2 f. ♃. bg. Ark. *Jas.*
1692. Ryhnchospora alba, *Vahl.* Swamp rush. July–Sept. 12 to 18 i. ♃. Swamps and bogs. Cin. O. *Colby.*
1693. R. glomerata, *Vahl.* July–Aug. 12 to 18 i. ♃. bg. mh. O. Ky.
1694. R. laxa, *Vahl.* July. 3 to 6 f. ♃. Swamps. Ky. *St.*

§ 3. *Caricinæ.*

*Carex.* Sedge. Sedge grass. Carex.

1695. Carex wildenovii, *Schk.* May–June. 8 to 12 i. ♃. Rocky woods. Ky. *St.* Hills, Cin. O.
1696. C. squarrosa, *Linn.* May–June. 2 f. ♃. bg. W. to Miss. *Bk.*
1697. C. muhlenbergii, *Schk.* May. 1 to 2 f. ♃. Rocky woods. ow. Cin. O. Ky. *St.*
1698. C. multiflora, *Muhl.* May. 2 f. ♃. Wet md. Ky. *St.*
1799. C. setacea, *Dew.* June–July. 18 to 30 i. ♃. Wet. md. Ky. ?*St.*
1700. C. arida, *Torr.* June. 2 to 3 f. ♃. md. Ohio and farther west. *Bk.*

1701. C. stellulata, *Good.* May. 8 to 18 i. ♃. Wet grounds. Ky.? *St.*
1702. C. scirpoides, *Schk.* May. 6 to 12 i. ♃ Wet md. Mich. Ter. *Houghton.*
1703. C. hirsuta, *Willd.* May. 12 to 18 i. ♃. md. rs. West to Mich. *Bk.* Ky. *St.*
1704. C. buxbaumii, *Wahl.* June. 2 f. ♃. Swamps. West to Michigan. *Bk.*
1705. C. digitalis, *Muhl.* May. 18 i. ♃. Wet md. West to Mich. *Bk.* Ky. *St.*
1706. C. aurea, *Nutt.* May–June. 4 to 10 i. ♃. Wet rocks. West to Mich. *Bk.* Mich. *Nutt.*
1707. C. varia, *Muhl.* April. 8 to 12 i. ♃. Dry woods. O. Ky.
1708. C. pubescens, *Muhl.* May. 12 to 18 i. ♃. Woods. Ky.? *St.*
1709. C. tentaculata, *Muhl.* May–June. 12 to 18 i. ♃. Wet md. Central O.
1710. C. lupulina, *Muhl.* June. 2 to 3 f. ♃. Swamps. Ky. *St.*
*b.* var. pedunculata, *Gray.* July. Shores of Lake Erie. *Gray.*
1711. C. folliculata, *Linn.* June. 18 i. ♃. Swamps. Ky. *St.* Miami country.
1712. C. plantaginea, *Lam.* Ap.–May. 8 to 12 i. ♃. Beech woods. O.
1713. C. anceps, *Muhl.* Ap.–May. 12 to 14 i. Dry woods. Ky. *St.* O.
1714. C. alba, *Hænke.* June. 4 to 10 i. Limestone hills. Ky. *St.*
1715. C. granularis, *Muhl.* May. 1 f. Wet grounds. Ky.? *St.*
1716. C. hystericina, *Willd.* May. 18 i. Wet grounds. Ky. *St.*
1717. C. limosa, *Linn.* June. 9 to 24 i. Swamps. Sault de Marie, Mich. Ter. *Houghton.*
1718. C. acuta, *Linn.* May. 2 f. Wet grounds. Ky. *St.*
1719. C. filiformis, *Linn.* 2 to 3 f. bg. West to Mich. *Bk.*
1720. C. vesicaria, *Linn.* May. 2 f. md. West to Mich. *Bk.*
1721. C. retrorsa, *Torr.* May. 2 f. About ponds. Ky. *St.*
1722. C. pellita, *Muhl.* May. 24 to 30 i. Wet grounds. Ky.? *St.*
1723. C. longirostris, *Torr.* 2 f. Wet md. West to Mich.—*Gray.*
1724. C. lacustris, *Willd.* June. 3 to 5 f. Swamps, &c. Near Cin. O.?

### CELLULARES.

### Tribe i. Filicoideæ. Fern-like plants.

### CCLXIII. *EQUISETACEÆ.*—The horse-tail tribe.

The equisetums are slightly astringent and stimulating, diuretic and emenagogue. Lind. Nat. Syst. 308.

1725. Equisetum hyemale, *Linn.* Scouring rush. June–July. 1 to 2 f. ♃. ow. mh. rb. W. The infusion is said to be an efficacious remedy for strangury.

1726. E. palustre, *Linn.* Marsh horse-tail. May–June. 12 to 18 i. ♃. mh. Swamps. Near Lake Sup. *Houghton.* Virginia. *Bk.*

1727. E. sylvaticum, *Linn.* Wood horse-tail. May. 12 to 18 i. ♃. Low woods. Central O.

1728. E. limosum, *Linn.* Swamp horse-tail. Naked horse-tail. July. 2 to 3 f. ♃. Borders of swamps. mh. Lake Sup. *Houghton.* Hoffman's prairie, Dayton, O.

1729. E fluviatile, *Linn.* Great water horse-tail. Fertile stems 1 f. Sterile stems 2 to 5 f. ♃. Shores of Lake Sup. Buffalo. *Torr.*

1730. E. arvense, *Linn.* Corn horse-tail. April–May. Fertile stems 6 to 8 i. Sterile stems 12 to 18 i. Fields, &c. W.

1731. E. variegatum, *Smith.* Variegated horse-tail. July. 3 to 6 i. ♃. Woods on high grounds. Near Lake Mich. *Houghton.*

### CCLXIV. *FILICES.*—The fern tribe.

The leaves of ferns generally contain a thick astringent mucilage, on which account they are considered pectoral and lenitive. The stem is bitter and astringent, anthelmintic, emenagogue and purgative. Lind. Nat. Syst. 311.

*Poloypdium.* Polypod.

1732. Polypodium vulgare, *Linn.* July. 6 to 10 i. ♃. Rocky woods. Ky. *St.* Mar. O. W. *Bk.*

1733. P. incanum, *Pursh.* July–Aug. ♃. Frond 3 to 6 i. Parasitic on the inclined moss-clad trunks of living trees. Ky. *St.* On rocks and trunks of old trees. Ky. Tenn. *Pursh.* Fl. ii. 659. I once met with this fern in a damp forest near Monroe, O., where it had crept to the height of 10 or 12 feet upon a large sycamore.

1734. P. hexagonopterum, *Michx.* July. 12 to 18 i. ♃. sw O. Ky.

1735. P. phlegopteris, *Linn.* *Bk.* P. connectile, *Willd.* Pale mountain polypod. July–Aug. 8 to 12 i. ♃. sw. O. Ky.

1736. Onoclea sensibilis, *Linn.* Sensitive polypod. July. 12 to 18 i. ♃. wp. Moist woods. O. Ky.

*Aspidium.* Shield fern.

1737. Aspidium acrostichoides. *Willd.* June–Aug. 12 to 18 i. ♃. rs. sw. W. Used by some as a vermifuge.

1738. A. thelypteris, *Willd.* Marsh shield-fern. July. 12 to 18 i. mh. wp. &c. O.

1739. A. cristatum, *Willd.* Crested shield-fern. July. 12 to 18 i. Marshy woods. Central O.

1740. A. lancastriense, *Spreng.* July. 18 to 24 i. ♃. Woods. Central O.?

1741. A goldianum, *Hook.* Goldie's shield-fern. July. 18 to 36 i. ♃. Woods. Central O.

1742. A fragile, *Willd.* A tenue, *Pursh.* Slender shield-fern. June–July. 6 to 14 i. ♃. sw. rs. &c. W.

1743. A marginale, *Willd.* Marginal shield-fern. July–Sept. 1 to 2 f. ♃. Rocky woods. Ky. *St.* Shady argillaceous ravines, Wor. O.

1744. A. spinulosum, *Willd.* Prickly toothed shield-fern. July. ♃. sw. (12 to 18 i. Central O.)?

1745. A. bulbiferum, *Willd.* July. 12 to 18 i. ♃. Wet rocks. Ky. *St.* Limestone ledges, Central O.

1746. A. filix-mas, *Willd.* Male fern. July. 2 to 3 f. Shady banks. *Hook.* Ky. *St.* Vermifuge, tonic, astringent. Wood & Bache.

1747. A. asplenoides, *Wild.* July. 1 to 2 f. ♃. sw. Mar. O.

*Asplenium.* Spleenwort.

1748. Asplenium rhizophyllum, *Willd.* Walking leaf. July. 4 to 10 i. ♃. Rocks. O. Ky.

1749. A. angustifolium, *Michx.* July. 12 to 18 i. ♃. Moist woods. sw. O. Ky.

1750. A. ebeneum, *Willd.* Ebony spleenwort. July. 6 to 10 i. ♃. Argillaceous hill-sides. rs. O. Ky.

1751. A. trichomanes, *Linn.* July. 4 to 8 i. ♃. Shady rocks. Ky. *St.* Near Cin. O. Resembles the preceding closely.

1752. A. thelypteroides, *Michx.* July. 1 to 2 f. ♃. Shady banks of streams. O. Ky.

1753. A. ruta-muraria, *Linn.* Rock-rue. July. 2 to 4 i. ♃. Rocks. Ky. *St.* Near Cin. O.

1754. A. montanum, *Willd.* Mountain spleenwort. July. 4 to 8 i. ♃. Mountain rocks. Ky. *St.*

1755. Scolopendrium officinarum, *Willd.* Hart's tongue. July. 8 to 15 i. ♃. sw. rs. Louisville. *M'Murt.* (?)
1656. Pteris aquilina, *Linn.* Brake. July. 2 to 6 f. ♃. Dry woods. ow. O. Ky. The stem is anthelmintic, emenagogue, and purgative. Lind. 311.
1757. P. atropurpurea, *Linn.* Rock brake. July. 6 to 10 i. ♃. Rocks. O. Ky.
1758. P. pedata, *Willd.* July. 6 i. ♃. Louisville. *M'Murt.*
1759. Adiantum pedatum, *Linn.* Maiden-hair. July. 1 to 2 f. ♃. sw. W. Pectoral, demulcent. Hoop. Dic. Raf. Med. Fl.
1760. Cheilanthes vestita, *Willd.* Lip-fern. July. 6 to 8 i. ♃. Rocks. West to Rocky Mts. *Bk.*
1761. C. dealbata, *Pursh.* July. 2 to 3 i. ♃. Crevices of rocks on the banks of the Missouri, 50 miles above its confluence. Rare. *Nutt.*
1762. Hypopeltis obtusa, *Willd.* July. 4 to 5 i. ♃. Rocks. Ky. *St.*

*Osmunda.* Flowering fern.

1763. Osmunda interrupta, *Michx.* June. 1 to 2 f. ♃. Low grounds. *Bk.* Louisville. *M'Murt.* Cin. O.
1764. O. cinnamonea, *Linn.* Aug. 2 to 5 f. ♃. Low grounds. Swamps. sw. Central O.
1765. O. regalis, *Linn.* July. 3 to 5 f. ♃. Wet woods, swamps. O. Ky. This fern has been given in 3 drachm doses for the rickets. Lind. 311.

1766. Ophioglossum vulgatum, *Linn.* Adder's tongue. June. 6 to 8 i. ♃. Low woods. Ky. *St.* Fig. Bart. Fl. N. A. ii. 55.
1767. O. bulbosum, *Michx.* May. 6 i. ♃. Low grounds. Ky. *St.* Fig. Bart. Fl. ii. 59.

*Botrychium.* Grape fern.

1768. Botrychium fumarioides, *Bk.* *Willd.* B. obliquum, *Muhl.* June. 9 to 12 i. ♃. sw. O. Ky.

1769. B. virginicum, *Swartz.* *Bk.* June–July. 12 to 20 i. ♃. sw. O. Ky.

CCLXV. *LYCOPODIACEÆ.*—The club-moss tribe.

*Lycopodium.* Club-moss. Ground-pine.

1770. Lycopodium carolinianum, *Linn.* July. Creeping. Peduncles 3 to 4 i. ♃. Low grounds. Northern O.?
1771. L. clavatum, *Linn.* July. Trailing. Branches 4 to 6 i. high. ♃. Pine woods. West to Mich. *Bk.* This plant excites vomiting. Woollens when boiled with this, or with other species, acquire the property of becoming blue, when passed through a bath of Brazil wood. *M. Vastring*. Lind. 314.

1772. L. complanatum, *Linn*. July. Trailing 2 to 10 f. ♃. Woods. Ky. *St*.

1773. L. dendroideum, *Michx*. July. Erect, 6 to 8 i. sw. Ky. *St*.

1774. L. annotinum, *Linn*. July. Creeping. (Branches 6 to 8 i. high. *Bk*.) Branches 3 to 5 i. high. ♃. Slaty ravines. rs. Central O.

CCLXVI. *MARSILEACEÆ*.—The pepperwort tribe.

1775. Azolla caroliniana, *Willd*. Floating on water. Leaves radical. ⊙. Louisville. *M'Murt*. Western states. *Nutt*.

Tribe II. Muscoideæ. Moss-like plants.

CCLXVII. *MUSCI*—The moss tribe.

In the fall and winter of 1832, I collected several species of moss, mostly from the damp slaty ravines near Worthington, Ohio. They were determined by Dr. Torrey of New York, (with the exception of Fontinalis,) and though quite limited in number, they are here subjoined.

1776. Bryum punctatum, *Schreb*. Erect. In damp shady places. Central O.

1777. Dicranum glaucum, *Hedwig*. Nov.–Dec. ½ to 1 i. Cæspitose. On the earth, logs, &c. Damp woods, ravines, &c. Central O.

1778. D. scoparium, *Hedwig*. Autumn and winter. 1 to 2 i. On rocks, the earth. &c. sw. O.

1779. Climacium dendroides, *Mohr*. 2 to 3 i. Damp shady places. O.

1780. Fontinalis capillacea, *Dicks*. Water moss. (Adhering to stones in running waters. 3 to 4 i. Mar. O. Miami country.)?

1781. F. antipyretica, *Spreng*. Syst. iv. 187. Pools near Dayton, O.?

1782. Neckera cladorhizans, *Hedwig*. Autumn. Creeping, on the trunks of trees. O.

1783. Polytrichum juniperinum, *Hedwig*. Hair-cap moss. May. 2 i. ♃. Dry woods. O.

1784. P. undulatum, *Hedwig*. 1 to 2 i. Central O.

1785. Arrhenopterum heterostichum, *Hedwig*. Autumn. Damp situations. Central O.

CCLXVIII. *HEPATICEÆ.*—The liver-wort tribe.

1786. Jungermannia multifida, *Linn.* 1 to 2 i. Swampy woods, Monroe, O.

1787. Marchantia polymorpha, *Linn.* Brook liver-wort. July. *g–y.* 2 i. ♃. Wet shady rocks, in wells, &c. O. Tonic, demulcent, nutrient. *De Cand.* Lind. 322.

1788. M. hemispherica, *Linn.* 1½ i. Rocky grounds. O. (?) Valuable diuretic, and local diaphoretic. *Eberle.*

1789. Riccia fluitans, *Linn.* Fork-stems. June. Floating, 2 to 3 i. Plenty from Lake Superior to Boston, in water among lemnæ. *Eat.*

CCLXIX. *CHARACEÆ.*—The chara tribe.

This order consists of a few obscure aquatic plants, found generally in stagnant pools. In Sprengel's Systema Vegetabilium, vol. iv. p. 345, (Ed. of 1827,) there are sixteen species of Chara enumerated, most of which occur in the temperate parts of the globe. I have not at present the means of comparing the plants which are described below, with foreign specimens, nor even with satisfactory descriptions. The specific names are therefore given more with a reference to present convenience, than with the expectation of their being ultimately retained.

1790. Chara vulgaris, *Willd.* Feather-beds. *Branhchlets* or *leaves* 7 to 9 in a whorl, bearing verticilli of still smaller branchlets. *Stem* 12 to 18 inches high, branching. Odour fetid, colour dirty green above, orange or yellow below. This species forms dense beds in stagnant pools and ditches. Like the other species it is encrusted with carbonate of lime. July–Aug. ⊙. O.

1791. C. flexilis, *Willd.* *Stems* seldom cæspitose, 18 to 24 inches high, moderately branching, smooth, semipellucid, effervesces slightly in muriatic acid; colour nearly brown; *branchlets* or *leaves* 4 to 7 in a whorl, from ½ to 2 inches in length, often branching; *internodes* 2 to 4 inches. July–Aug. ⊙. Grows in clear still water, 2 miles south of Dayton, and 7 miles north of Cincinnati.

1792. C. squamosa, *Desfontaines.* (?) foliosa, *Willd.* (?) *Stem* solitary, calcareous and very brittle, sending off at different heights 2 or 3 branches at an angle near 35°; scabrous, striate, subsulcate, the younger portions armed with numerous whorls of minute foliaceous scales. *Branchlets* generally 13 in a whorl, from one half to three fourths of an inch long, involucred at their origin by a dense whorl of subulate scales. *Thecæ* 2, 3 or

4, on the inner side and lower half of each branchlet. On each branchlet there are from 4 to 7 whorls of nearly obsolete scales. Aug. 8 to 10 i. ☉. In shallow, slow running water. Old River, Hamilton, O. Oolentangy River, Worthington, O.

1793. C. humilis, *mihi*. *Stem* calcareous, solitary and branching, scabrous, (not sulcate,) 2 to 3 inches high.—*Branchlets* 8 to 10 in a whorl, without an involucre, generally equalling the internodes which do not exceed three-fourths of an inch in length. Towards the summit of the stem, each branchlet bears 1 or 2 thecæ. Colour deep green. Aug. ☉. Growing in the shallow water of Old River, with the last, which it resembles.

1794. C. sabulosa, *mihi*. Stone-wort. *Stem* with a few erect branches, subsulcate, papillose; *branchlets* generally 10, sometimes 8 in a whorl, without an involucre, near half an inch long, each with 2 to 4 verticilli of scales, in the axils of which, on the inner side of the branchlet, are contained the thecæ. *Internodes* near an inch long. Aug. ☉. 18 inches high. Colour light pea green. Four or five plants generally grow in company in clear pools where the water is two or three feet deep. Dayton, O. The calcareous incrustation is unusually thick, and the stems remarkably brittle.

*b*. var. spiralis, *mihi*. *Stem* spirally sulcate; compared with the last, the branches are more divaricate, and the internodes and branchlets twice as long in proportion to the size of the stem. Pools near Dayton.

### Tribe III. Aphyllæ. Leafless flowerless plants.

### CCLXX. *LICHENES*.—The lichen tribe.

1795. Cenomyce pyxidata. Autumn and winter. Cinereous g. ½ to 1 i. On the earth. Wor. O. (The names accord with the Synopsis Methodica Lichenum of Acharius.)

1796. C. bacillaris. Nov. *r*. ½ i. On the earth. Central O.

1797. C. coccifera. Autumn. *r*. ½ i. Earth. O.

1798. C. rangiferina. Rein-deer moss. March. Lichen cinereous. 2 to 4 i. Shady, rocky ravines. Wor. O. Earth. In colder regions this lichen grows much more luxuriantly. The rein-deer subsists upon it during the Greenland winter.

1799. Peltidea canina. In fruit at all seasons. Lichen bluish cinereous, or greyish green. 1 to 2 i. On the earth, among mosses, in damp rocky situations. O.

Qualities like the Iceland moss, to which it is pretty closely allied.

1800. Sticta pulmonacea. Lungwort. Yellowish cinereous. 1 to 2 i. On the bark of trees. W. Tonic, demulcent, nutrient, like the Iceland moss. (Cetraria islandica. Lind. Nat. Syst. 329.)

1801. Usnea florida. Greenish grey. 2 to 3 i. Winter. On the branches of trees. W.

1802. U. strigosa. Dirty cinereous. 2 to 3 i. On the trunks and branches of dead trees. Dayton, O.

### CCLXXI. *FUNGI.*—THE MUSHROOM TRIBE.

A most extensive natural order, embracing an almost indefinite number of species, that hold their rank in the lowest grades of the vegetable kingdom. They occur plentifully in the Western States; but (were it possible to do it) it would not comport with the design and limits of this publication to give a synopsis of them.

### CCLXXII. *ALGÆ.*—THE SEA-WEED TRIBE.

This order embraces those obscure productions of the aqueous element, which are known under the names of *sea-weed*, *frog-spittle*, *river-hair*, and *green scum*. Our fresh water algæ have received very little attention, though they are met with in every fountain and stagnant pool. They make the most delicate and beautiful specimens, when properly preserved. In fitting them for the herbarium, the following method may be pursued.

Cut a sheet of writing paper into 8, 16 or 32 equal pieces, according to the proposed size of the specimens. Wash the algæ by gently moving or shaking them inwater;—detach enough for a specimen, carry a piece of paper underneath, and raise it slowly from the water. By a short exposure to the air or to sunshine, the algæ will become so dry as to be in no danger of adhering to the leaves of a port-folio. They may then be pressed and thoroughly dried, between folds of bibulous paper, like other plants. (See West. Jour. Med. Phys. Sci. viii. 29.) If these manipulations are skilfully accomplished, the minutest filaments are elegantly displayed upon the paper, and remain thereunto permanently attached. The green scum that is often seen on stagnant waters, may be fished up at once by paper without previous washing. In my botanical rambles through the Miami country, last season, I collected and preserved in this manner near a dozen submersed, and two or three floating species.

### REFERENCE TO AUTHORITIES.

WISHING to present this synopsis in a small compass, I remarked in the preface, that the authority for specific names, when not quoted, might be found in Eaton's Manual of Botany. After the first two sheets were printed, I came to the determination of making the usual references to authors. The previous omissions are here supplied, excepting the references to Linnæus. The figures refer to the species.

Species number 1, 2, and 3, Willd. 9, Michx. 16, 17, 18, De Cand. 20, 21, 24, Michx. 25, Willd. 26, Nutt. 27, Walt, 29, Nutt. 30, Big. 33, Pursh. 35, 37, Willd. 38, Walt. 40, Michx. 42, Nutt. 45, De Cand. 46, Rose. 49, 52, 53, De Cand. 57, Muhl. 58, Lam. 59, Willd. 60, Pursh. 61, Michx. 64, Brown. 67, Raf. 68, Willd. 70, Salis. 73, 74, Michx. 75, Willd. 76, Nutt. 79, Eat. 80, Big. 81, L'Herit. 82, Willd. 83, Michx. 84, Nutt. 86, Willd. 87, Ait. 90, Willd. 91, Michx. 92, Muhl. 94, Bart. 100, Pers. 101, Hook. 103, 104, 105, 106, Willd. 107, 108, 109, Michx. 110, Willd. 111, 112, 113, Nutt. 114, Walt. 115, 120, Michx. 123, Nutt. 124, Smith. 125, Nutt. 131, Willd. 132, Ait. 134, Pursh. 135, De Cand. 137, Willd. 138, 139, 140, Michx. 141, 142, 149, Willd. 150, Pursh, 154, Michx. 155, Nutt. 157, Willd. 159, Michx. 161, 162, 163, Vent. 164, 165, 166, Willd. 167, Lam. 169, 170, Willd. 171, Pursh. 175, Michx. 176, 177, Vent. 178, 179, Michx. 180, Willd. 184, Michx. 186, 187, Pursh. 188, Raf. 190, Willd. 191, Lam. 193, Muhl. 194, 195, Willd. 196, Nutt. 197, 199, Michx. 200, Willd. 201, Michx. 203, Nutt. 204, Pursh. 206, Lam. 207, Muhl. 208, Willd. 209, Muhl. 211, Nutt. 214, Ait. 215, Roth. 216, Willd. 217, 218, Nutt. 219, Pursh. 220, Nutt, 221, Pursh. 222, Raf. 223, Willd. 224, Nutt. 225, Pursh. 227, Walt. 228, 230, Willd. 235, 236, 238, Nutt. 239, Pursh. 240, Willd. 242, 244, Michx. 245, Pursh. 247, Nutt. 248, L'Herit. 251, Walt. 253, Michx. 255, Willd. 258, Michx. 265, Willd. 267, Michx. 268, Lam. 269, Michx. 271, Ait. 273, 275, Willd. 276, Pursh. 278, Michx. 281, Ait. 283, Nutt. 284, Michx. 286, Brown. 287, 289, Willd. 291, Ehrh. 292, Ait. 295, Pallas. 296, Willd. 298, Mœnch. 299, Bart, 301, Pers. 304, Nutt. 306, Willd. 307, Walt. 308, Ait. 309, 310, Willd. 311, 312, Michx. 313, Willd. 316, Ait. 318, Pursh. 319, Marsh. 320, Michx. 321, Willd. 322, Michx. 324, Muhl. 325, Nutt. 328, 229, Pursh. 330, Nutt. 331, Pursh. 332, Nutt. 333, Michx. 334, Torr. 336, Willd. 337, Muhl. 340, Michx. 341, Willd. 342, Michx. 343, 344, Pursh. 345, Michx. 346, Muhl. 349, Nutt. 350, Michx. 351, Willd. 352, Ell, 353, Michx. 357, Willd. 358, Nutt. 360, Michx. 361, Willd. 362, Pursh. 363, Willd. 365, Michx. 366, Willd. 368, 371, 372, Nutt. 374, 375, Willd. 376, Torr. 377, Nutt. 378, Pursh. 379, Torr. 380, Willd. 381, Michx. 382, Pursh. 383, Nutt. 385, Pursh. 386, 387, Nutt. 388, Pursh. 390, 391, Nutt. 392, Michx. 396, Ell. 397, Eat. 398, Willd. 399, Mœnch. 400, Walt. 401, Ell. 402, Pers. 403, Nutt. 405, 406, Pursh. 407, Willd. 410, Walt. 413, 414, De Cand. 419, Willd. 420, Muhl. 421, 432, 434, Willd. 436, 437, 438, Michx. 439, 440, Willd. 441, Pursh. 442, Walt. 443, Nutt. 444, Willd. 445, Michx. 446, Ait. 447, Willd. 448, 449, 450, Michx. 452, Willd. 454, Bart. 456, Michx. 457, Willd. 458, Walt. 559, Michx. 461, Willd. 462, Wang. 463, Michx. 466, Walt. 467, Ait. 469, 472, Michx. 473, Willd. 474, Ait. 475, 478, 479, Willd. 481, Wangh. 482, 483, Pursh. 484, Muhl. 485, Willd. 486, Muhl. 487, Willd. 489, Michx. 491, Willd. 492, Ait. 493, 494, Michx. 498, Willd. 499, Ait. 502, 503, 504, 505, 506, 507, Nutt. 508, Willd. 509, Pursh. 510, 512, Willd. 513, Jacq. 515, Michx. 516, Pursh. 517, Michx. 519, Willd. 521, Michx. 522, Nutt. 523, 527, Michx. 528, 529, Willd. 530, Juss. 531, Willd. 532, Nutt. 523, Willd. 534, 535, 537, Pursh.

### INDEX TO THE GENERA.

| Genus | No. |
|---|---|
| Abies | 1386 |
| Acalypha | 524 |
| Acer | 546 |
| Acerates | 1096 |
| Achillea | 1027 |
| Acnida | 683 |
| Aconitum | 78 |
| Acorus | 1520 |
| Actæa | 80 |
| Actinella | 976 |
| Actinomeris | 984 |
| Adiantum | 1759 |
| Ægilops | 1648 |
| Æsculus | 540 |
| Agave | 1401 |
| Agathyrsus | 816 |
| Agrimonia | 270 |
| Agrostemma | 629 |
| Agrostis | 1535 |
| Aira | 1585 |
| Aletris | 1467 |
| Alisma | 1390 |
| Alliona | 726 |
| Allium | 1460 |
| Alnus | 471 |
| Alopecurus | 1550 |
| Alyssum | 112 |
| Amarantus | 666 |
| Amaryllis | 1404 |
| Ambrosia | 1014 |
| Ammannia | 242 |
| Amorpha | 389 |
| Ampelopsis | 556 |
| Amphicarpa | 396 |
| Amsonia | 1106 |
| Anagallis | 1171 |
| Androcera | 1246 |
| Andromeda | 741 |
| Andropogon | 1649 |
| Androsace | 1158 |
| Anemone | 39 |
| Angelica | 21 |
| Anthemis | 1025 |
| Anthoxanthum | 1584 |
| Antirrhinum | 1194 |
| Apios | 399 |
| Aplectrum | 1419 |
| Apocynum | 1102 |
| Aquilegia | 72 |
| Arabis | 100 |
| Aralia | 1 |
| Arbutus | 738 |
| Archemora | 14 |
| Arctium | 822 |
| Arenaria | 648 |
| Argemone | 68 |
| Aristida | 1581 |
| Aristolochia | 248 |
| Arnica | 848 |
| Aronia | 300 |
| Arrhenatherum | 1590 |
| Arrhenopterum | 1785 |
| Artemisia | 1030 |
| Arum | 1515 |
| Arundinaria | 1661 |
| Asarum | 250 |
| Asclepias | 1086 |
| Ascyrum | 180 |
| Asimina | 135 |
| Aspidium | 1737 |
| Asplenium | 1748 |
| Ater | 857 |
| Astragalus | 366 |
| Atheropogon | 1635 |
| Atriplex | 679 |
| Azolla | 1775 |
| Azalea | 750 |
| Baptisia | 231 |
| Barbarea | 99 |
| Bartonia | 234 |
| Batschia | 1347 |
| Beckmannia | 1571 |
| Bellis | 924 |
| Berberis | 146 |
| Betula | 498 |
| Bignonia | 1256 |
| Bletia | 1431 |
| Blitnm | 685 |
| Bœhmeria | 431 |
| Bœrbera | 954 |
| Botrychium | 1768 |
| Brodiæa | 1469 |
| Bromus | 1616 |
| Brunnichia | 712 |
| Bryum | 1776 |
| Buchnera | 1192 |
| Bumelia | 737 |
| Cacalia | 940 |
| Cactus | 202 |
| Cælestina | 936 |
| Cakile | 119 |
| Calamagrostis | 1582 |
| Calligonum | 714 |
| Callitriche | 230 |
| Callopogon | 1417 |
| Caltha | 68 |
| Calypso | 1432 |
| Campanula | 765 |
| Cannabis | 434 |
| Capraria | 1212 |
| Cardamine | 106 |
| Cardiospermum | 545 |
| Carduus | 823 |
| Carex | 1695 |
| Carpinus | 473 |
| Carya | 502 |
| Cassia | 416 |
| Castanea | 464 |
| Catalpa | 1258 |
| Caulinia | 1521 |
| Ceanothus | 536 |
| Celastrus | 529 |
| Celtis | 439 |
| Cenchrus | 1572 |
| Cenomyce | 1794 |
| Centunculus | 1172 |
| Cephalanthus | 1054 |
| Cerastium | 641 |
| Ceratophyllum | 731 |
| Cercis | 423 |
| Ceresia | 1570 |
| Cineraria | 952 |
| Chara | 1790 |
| Chærophyllum | 32 |
| Cheilanthes | 1760 |
| Cheiranthes | 95 |
| Chelidonium | 82 |
| Chelone | 1203 |
| Chenopodium | 672 |
| Chianothus | 1152 |
| Chimaphila | 761 |
| Chloris | 1632 |
| Chrysanthemum | 1013 |
| Chrysocoma | 922 |
| Chrysopsis | 849 |
| Chrysosplenium | 189 |
| Cicuta | 10 |
| Cinna | 1544 |
| Circæa | 232 |
| Claytonia | 656 |
| Clematis | 36 |
| Cleome | 134 |
| Climacium | 1779 |
| Cochlearia | 128 |
| Clinopodium | 1328 |
| Clitoria | 394 |
| Collinsia | 1211 |
| Collinsonia | 1294 |
| Collomia | 1148 |
| Comarum | 265 |
| Commelina | 1396 |
| Comptonia | 499 |
| Conium | 34 |
| Convallaria | 1480 |
| Convolvulus | 1124 |

Conyza 846
Coptis 70
Corallorhiza 1418
Coreopsis 988
Corispermum 684
Cornus 1075
Coronopus 124
Corydalis 131
Corylus 466
Crategus 305
Croton 521
Crotonopsis 523
Crypsis 1552
Crypta 654
Cucubalus 631
Cunila 1306
Cuphea 237
Cupressus 1380
Cuscuta 1134
Cynoglossum 1357
Cyperus 1662
Cypripedium 1434
Dactylis 1613
Dalea 377
Dalibarda 268
Danthonia 1589
Darlingtonia 413
Datura 1243
Daucus 35
Decodon 241
Delphinium 73
Dentaria 109
Diapensia 1149
Diarrhena 1631
Diclytra 129
Dicranum 1777
Diervilla 1059
Digitaria 1566
Dioscorea 1501
Diospyros 1150
Diotis 690
Dipsacus 784
Dirca 255
Dodecatheon 1159
Donia 939
Dracocephalum 1313
Draba 114
Drosera 618
Dulichium 1672
Echinospermum 1356
Eclipta 977
Elæagnus 245
Elephantopus 831
Eleusine 1633
Ellisia 1373
Elymus 1640
Enslenia 1098
Epigæa 752
Epilobium 206
Epiphegus 1182
Equisetum 1725
Erigenia 6
Erigeron 894
Eriocaulon 1509
Eriogonum 713
Eriophorum 1689
Eryngium 8
Erythronium 1507
Euchroma 1227
Euonymus 530
Eupatorium 925
Euphorbia 508
Evolvulus 1133
Fagus 467
Ferula 26
Festuca 1607
Flœrkea 92
Fontinalis 1780
Fragaria 266
Frasera 1122
Fraxinus 1153
Fuinera 1691
Galardia 979
Galax 659
Galega 364
Galeopsis 1309
Galium 1042
Gaultheria 739
Gaura 210
Gentiana 1107
Geranium 567
Gerardia 1214
Geum 273
Gillenia 298
Glechoma 1322
Gleditschia 422
Glyceria 1626
Glycyrrhiza 387
Gnaphalium 852
Goodyera 1412
Gonolobus 1099
Gratiola 1198
Gymnocladus 421
Habenaria 1422
Hamamelis 195
Hedeoma 1325
Hedysarum 348
Helenium 986
Helianthemum 616
Helianthus 955
Heliopsis 987
Heliotropum 1362
Helonias 1451
Hepatica 52
Heracleum 24
Herpestris 1213
Hesperis 120
Heteranthera 1455
Heuchera 185
Hibiscus 156
Hieracium 788
Hippuris 229
Holcus 1588
Hordeum 1637
Hottonia 1161
Houstonia 1112
Hudsonia 614
Humulus 435
Hydrangea 1083
Hydrastis 51
Hydropeltis 91
Hydrophyllum 1364
Hyoscyamus 1245
Hypericum 164
Hypopeltis 1762
Hypoxis 1402
Hyssopus 1289
Ilex 733
Impatiens 574
Inula 847
Iresine 671
Iris 1405
Isanthus 1279
Isnardia 228
Isopyrum 71
Itea 194
Iva 1021
Jeffersonia 94
Juglans 500
Juncus 1438
Jungermannia 1786
Juniperus 1375
Jussieua 224
Justicia 1250
Kalmia 747
Kochia 688
Kœleria 1614
Krigia 806
Kuhnia 937
Kyllingia 1673
Lactuca 810
Lamium 1311
Larix 1389
Lathyrus 406
Laurus 144

| | | | | | |
|---|---|---|---|---|---|
| Lechea | 611 | Mitella | 190 | Petalostemon | 374 |
| Lecontia | 1517 | Mollugo | 635 | Phaca | 381 |
| Leersia | 1658 | Momordica | 774 | Phacelia | 1369 |
| Lemna | 1532 | Monarda | 1295 | Phalangium | 1468 |
| Leontice | 147 | Monotropa | 763 | Phalaris | 1548 |
| Leontodon | 805 | Morus | 444 | Phaseolus | 400 |
| Leonurus | 1310 | Muhlenbergia | 1546 | Philadelphus | 196 |
| Lepidium | 126 | Myosotis | 1353 | Phleum | 1545 |
| Leptandra | 1191 | Myosurus | 67 | Phlox | 1136 |
| Lepturus | 1647 | Myrica | 497 | Phryma | 1272 |
| Lespedeza | 340 | Myriophyllum | 231 | Physalis | 1233 |
| Liatris | 838 | Myrrhis | 31 | Phytolacca | 692 |
| Ligusticum | 20 | Nasturtium | 96 | Pinus | 1382 |
| Ligustrum | 1151 | Neckera | 1782 | Piptatherum | 1565 |
| Lilium | 1502 | Nelumbium | 90 | Pisum | 412 |
| Lindernia | 1201 | Nemophila | 1368 | Planera | 442 |
| Linnæa | 1060 | Nepeta | 1317 | Plantago | 776 |
| Linum | 620 | Nicandra | 1239 | Platanthera | 1427 |
| Liparis | 1428 | Nicotiana | 1240 | Poa | 1591 |
| Liriodendron | 143 | Nuphar | 88 | Podophyllum | 93 |
| Lithospermum | 1343 | Nuttallia | 155 | Podostemum | 730 |
| Lobelia | 758 | Nymphæa | 87 | Podostigma | 1097 |
| Lonicera | 1057 | Nyssa | 251 | Pogonia | 1415 |
| Ludwigia | 226 | Obolaria | 1120 | Polanisia | 133 |
| Lupinus | 404 | Œnothera | 212 | Polemonium | 1135 |
| Luzula | 1446 | Onoclea | 1736 | Polycnemum | 691 |
| Lycopodium | 1770 | Onosmodium | 1349 | Polygala | 576 |
| Lycopsis | 1352 | Ophioglossum | 1766 | Polygonum | 693 |
| Lycopus | 1281 | Orchis | 1421 | Polymnia | 1004 |
| Lysimachia | 1162 | Origanum | 1288 | Polypodium | 1732 |
| Lythrum | 238 | Orobanche | 1178 | Polypogon | 1543 |
| Maclura | 443 | Orobus | 383 | Polytrichum | 1783 |
| Macrotys | 79 | Orontium | 1519 | Pontederia | 1458 |
| Magnolia | 136 | Orthocarpus | 1226 | Populus | 489 |
| Malva | 160 | Oryzopsis | 1656 | Portulacca | 655 |
| Marchantia | 1787 | Osmunda | 1763 | Potamogeton | 1522 |
| Mariscus | 1690 | Ostrya | 474 | Potentilla | 257 |
| Marrubium | 1323 | Oxalis | 569 | Prenanthes | 793 |
| Marshallia | 982 | Oxycoccus | 757 | Primula | 1157 |
| Martynia | 1255 | Oxydenia | 1634 | Prinos | 734 |
| Meconopsis | 83 | Oxytropis | 385 | Prunella | 1329 |
| Medeola | 1488 | Panax | 4 | Prunus | 315 |
| Medicago | 326 | Pachysandra | 527 | Psoralea | 327 |
| Melampyrum | 1225 | Pancratium | 1403 | Ptelea | 566 |
| Melanthium | 1447 | Panicum | 1553 | Pteris | 1756 |
| Mellissa | 1324 | Parietaria | 433 | Pulmonaria | 1360 |
| Melica | 1627 | Parnassia | 193 | Pycnanthemum | 1282 |
| Menispermum | 148 | Parthenium | 1024 | Pyrola | 758 |
| Mentha | 1273 | Paspalum | 1573 | Pyrus | 314 |
| Mentzelia | 236 | Passiflora | 609 | Quercus | 445 |
| Menyanthes | 1119 | Pastinaca | 28 | Queria | 663 |
| Microstylis | 1430 | Pedicularis | 1222 | Ranunculus | 54 |
| Mikania | 938 | Peltidea | 1797 | Rhamnus | 533 |
| Mimulus | 1196 | Penthorum | 662 | Rhexia | 243 |
| Mitchella | 1056 | Pentstemon | 1204 | Rhododendron | 746 |

| | | | | | |
|---|---|---|---|---|---|
| Rhus | 559 | Solea | 608 | Triglochin | 1531 |
| Rhynchospora | 1692 | Solidago | 904 | Trigonella | 388 |
| Ribes | 197 | Sonchus | 819 | Trillium | 1492 |
| Riccia | 1789 | Sophora | 235 | Triosteum | 1061 |
| Robinia | 393 | Sorbus | 313 | Triphora | 1420 |
| Rosa | 286 | Sparganium | 1512 | Tripsacum | 1576 |
| Rubus | 278 | Spartina | 1645 | Triticum | 1639 |
| Rudbeckia | 967 | Spermacoce | 1055 | Trollius | 69 |
| Ruellia | 1252 | Spergula | 633 | Troximon | 808 |
| Rumex | 715 | Spigelia | 1123 | Tullia | 1308 |
| Sabbatia | 1121 | Spiræa | 292 | Tussilago | 944 |
| Sagina | 632 | Spiranthes | 1413 | Typha | 1510 |
| Sagittaria | 1391 | Stachys | 1319 | Udora | 1394 |
| Salix | 475 | Stanleya | 125 | Ulmus | 436 |
| Salvia | 1340 | Staphylea | 539 | Uniola | 1623 |
| Sambucus | 1073 | Starkea | 980 | Uraspermum | 29 |
| Samolus | 1173 | Stellaria | 636 | Urtica | 424 |
| Sanguinaria | 85 | Sticta | 1798 | Usnea | 1799 |
| Sanguisorba | 256 | Stipa | 1577 | Utricularia | 1174 |
| Sanicula | 7 | Streptopus | 1487 | Uvularia | 1489 |
| Saponaria | 630 | Strophostyles | 401 | Vaccinium | 753 |
| Sarothra | 182 | Stylosanthes | 373 | Valeriana | 785 |
| Sarracenia | 617 | Symphitum | 1351 | Valerianella | 786 |
| Saururus | 729 | Symphoria | 1063 | Vallisneria | 1395 |
| Saxifraga | 183 | Symplocarpus | 1518 | Veratrum | 1452 |
| Schollera | 1457 | Synandra | 1318 | Verbascum | 1247 |
| Schrankia | 415 | Sysimbrium | 122 | Verbena | 1259 |
| Scirpus | 1674 | Talinum | 658 | Verbesina | 1003 |
| Scolopendrium | 1755 | Tanacetum | 1028 | Vernonia | 832 |
| Scrophularia | 1193 | Taxus | 1379 | Veronica | 1183 |
| Scutellaria | 1330 | Teucrium | 1291 | Viburnum | 1064 |
| Sedum | 660 | Thalictrum | 44 | Vicia | 407 |
| Senecio | 945 | Thapsia | 19 | Viola | 588 |
| Seseli | 33 | Thermia | 386 | Virgilia | 392 |
| Sesleria | 1630 | Thesium | 254 | Viscum | 1085 |
| Setaria | 1563 | Thlaspi | 116 | Vitis | 552 |
| Seymeria | 1221 | Thymus | 1287 | Wistaria | 403 |
| Shepherdia | 246 | Tiarella | 192 | Xanthium | 1020 |
| Sibbaldia | 285 | Tilia | 161 | Xanthoriza | 81 |
| Sicyos | 775 | Tofielda | 1450 | Xanthoxylum | 565 |
| Sida | 151 | Tradescantia | 1398 | Xyris | 1400 |
| Silene | 623 | Tragia | 528 | Yucca | 1506 |
| Silphium | 1006 | Trichodium | 1549 | Zapania | 1269 |
| Sinapis | 127 | Tricophorum | 1687 | Zea | 1660 |
| Sisyrinchium | 1409 | Trichophyllum | 981 | Zigadenus | 1454 |
| Sium | 12 | Trichostemma | 1293 | Zinnia | 851 |
| Smilax | 1470 | Tricuspis | 1629 | Zizania | 1654 |
| Solanum | 1229 | Trifolium | 335 | Zizia | 16 |

